# Supplementary material for: Ethylene electrosynthesis from low-concentrated acetylene via concave-surface enriched reactant and improved mass transfer
Source: Nat Commun. 2024 Jul 13;15:5914. doi: 10.1038/s41467-024-50335-8 (PMC11246534; doi:10.1038/s41467-024-50335-8)
Supplement: Supplementary file 1 — Supplementary Information [file 41467_2024_50335_MOESM1_ESM.pdf]

Supplementary Information

Ethylene electrosynthesis from low-concentrated acetylene via concave-surface enriched reactant and improved mass transfer

Chen *et al*.

Contents

Supplementary Figure 1. Theoretical prediction of C_2H_2 adsorption over the Cu.
Supplementary Figure 2. Theoretical simulation of C_2H_2 diffusion over the PCC.
Supplementary Figure 3. Theoretical simulation of C_2H_2 diffusion over C.
Supplementary Figure 4. Theoretical length distribution of the C-H bond.
Supplementary Figure 5. Initial morphology characterization of the Cu-MOF precursors.
Supplementary Figure 6. Optimization of the TA exchange process.
Supplementary Figure 7. Morphological characterization of Cu-TA.
Supplementary Figure 8. SEM, TEM, and HRTEM images of Cu-PCC and the corresponding energy-dispersive X-ray spectroscopy (EDS) elemental mapping images.
Supplementary Figure 9. SEM, TEM, and HRTEM images of Cu-C and the corresponding energy-dispersive X-ray spectroscopy (EDS) elemental mapping images.
Supplementary Figure 10. The statistical size distributions.
Supplementary Figure 11. AFM characterization.
Supplementary Figure 12. The sequential conversion process from Cu-MOF precursors to Cu-PCC.
Supplementary Figure 13. Characterization of the samples calcinated under a H_2 atmosphere.
Supplementary Figure 14. Surface functional group characterization.
Supplementary Figure 15. The valence state and coordinate environment of Cu sites.
Supplementary Figure 16. Contact angle analysis.
Supplementary Figure 17. Specific surface area analysis.
Supplementary Figure 18. Illustrations of the electrochemical setups.
Supplementary Figure 19. The LSV curve and the corresponding Tafel slopes of Cu-PCC and Cu-C.
Supplementary Figure 20. Enlarged DEMS in LSV mode of Cu-PCC and Cu-C.
Supplementary Figure 21. The calibration curves used for product quantification.
Supplementary Figure 22. Electrochemical capacitance measurements of Cu-PCC and Cu-C.
Supplementary Figure 23. ICP−OES analysis of the two catalysts.
Supplementary Figure 24. Normalized performance comparison.
Supplementary Figure 25. The stability of Cu-PCC after 12 h of continuous testing.
Supplementary Figure 26. Kinetic isotope effect comparison.
Supplementary Figure 27. The recognition of characteristic peaks of H_2O and C_2H_2.
Supplementary Figure 28. Further recognition of C_2H_2 peaks was performed using the isotope effect.
Supplementary Figure 29. Peak-differentiating and fitting analysis of the ATR-FTIR data of Cu-PCC and Cu-C under a C_2H_2 atmosphere.
Supplementary Figure 30. The results of ATR−FTIR.

Supplementary Figure 31. Peak differentiation and fitting analysis.
Supplementary Figure 32. Recognition of the Raman characteristic peaks of C_2H_2.
Supplementary Figure 33. The optimized structures used for theoretical calculations.
Supplementary Figure 34. Energy profiles and optimized adsorption structures from C_2H_2 to C_2H_3 with high C_2H_2 coverage.
Supplementary Figure 35. Energy profiles and optimized adsorption structures from C_2H_3 to C_2H_4 with high C_2H_2 coverage.
Supplementary Figure 36. Energy profiles and optimized adsorption structures from C_2H_2 to C_2H_3 with low C_2H_2 coverage.
Supplementary Figure 37. Energy profiles and optimized adsorption structures from C_2H_3 to C_2H_4 with low C_2H_2 coverage.
Supplementary Table 1. Comparison of the performance of the Cu-PCC and the state-of-the-art reports on ESAE.
Supplementary Notes 1−13.
Supplementary References (1−6).

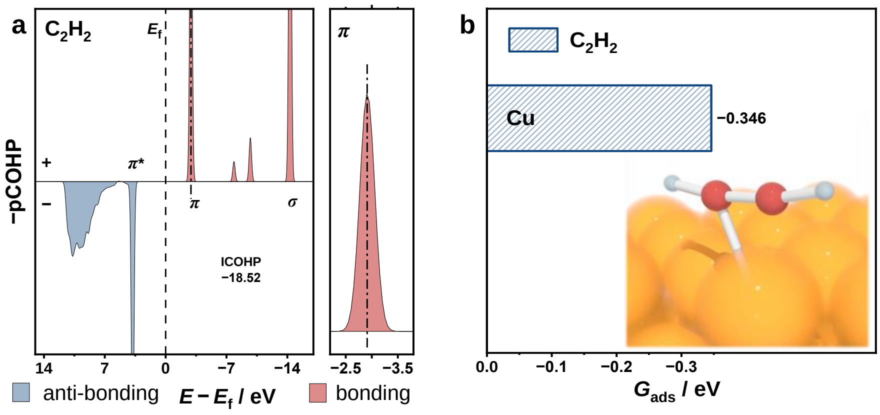

Supplementary Figure 1. Theoretical prediction of C_2H_2 adsorption over the Cu (a) Projected crystal orbital Hamilton population (−pCOHP) for the C−C interaction of gaseous C_2H_2. (b) Adsorption of C_2H_2 on pure Cu.

Supplementary Note 1. As shown in Supplementary Figure 1, compared with those of PCC and C (Fig. 2c), the adsorption of C_2H_2 on Cu is greater than that on carbon supports, indicating that hydrogenation is prone to occur at Cu sites.

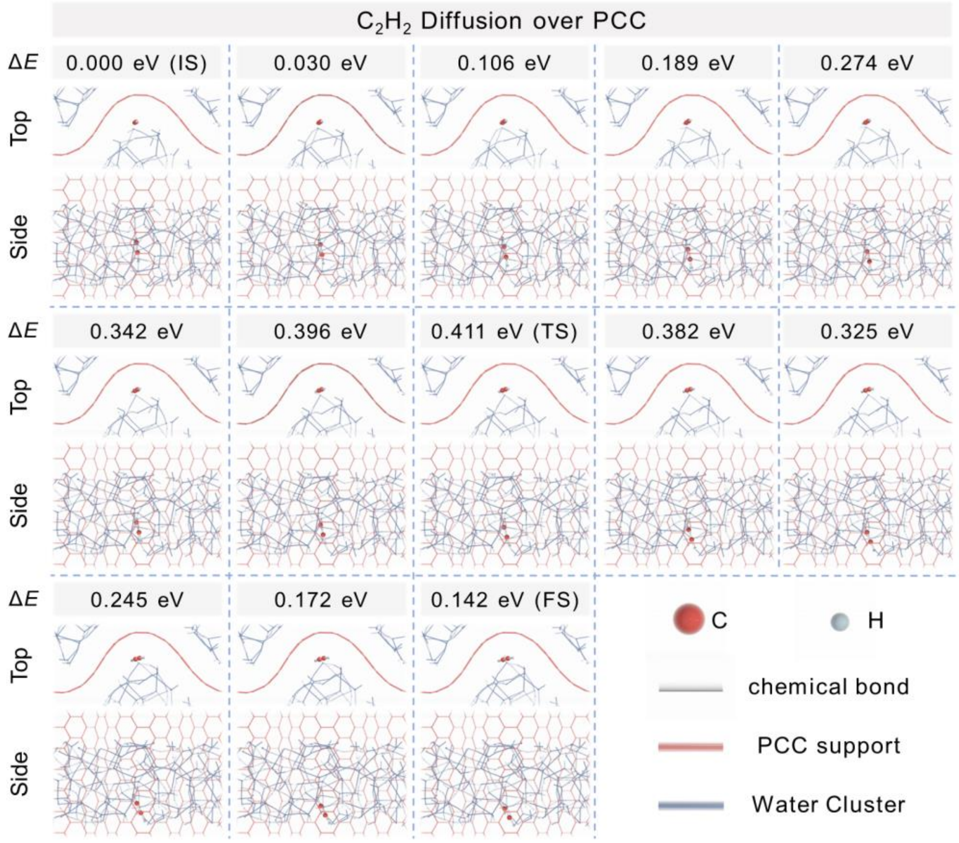

Supplementary Figure 2. Theoretical simulation of C_2H_2 diffusion over PCC. The energy change profiles and theoretical adsorption models of the transition states for C_2H_2 migration over carbon with a concave surface.

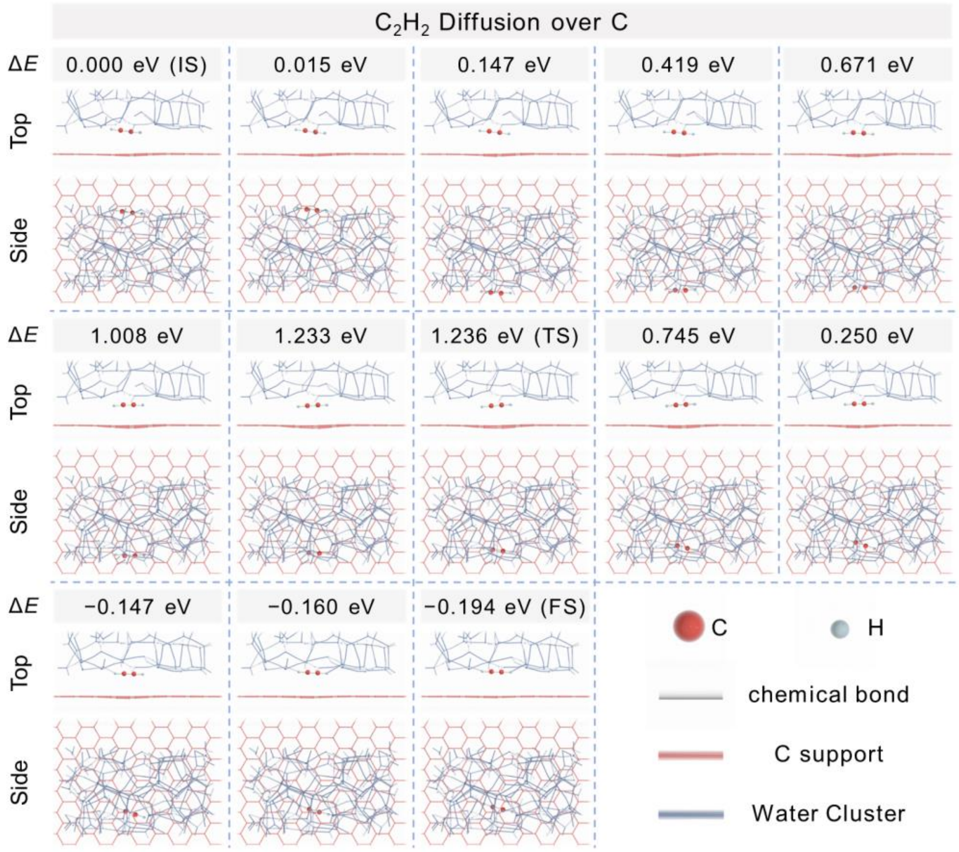

Supplementary Figure 3. Theoretical simulation of C_2H_2 diffusion over C. The energy change profiles and theoretical adsorption models of the transition states for C_2H_2 migration over carbon with a plane surface.

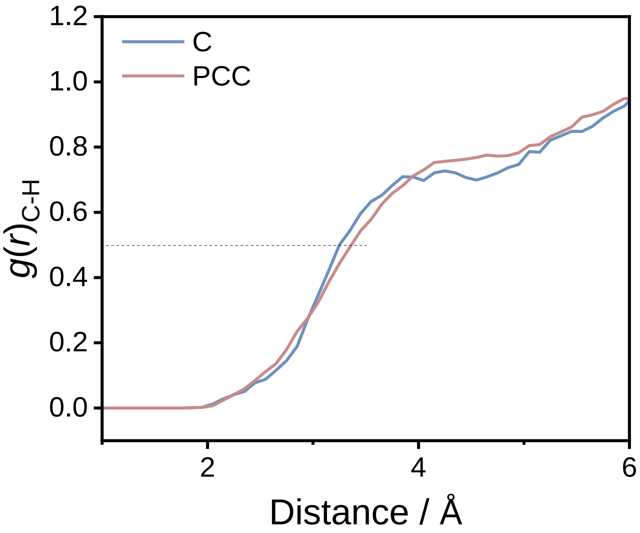

Supplementary Figure 4. Theoretical length distribution of the C−H bond. The calculated RDFs of C−H in the PCC and C.

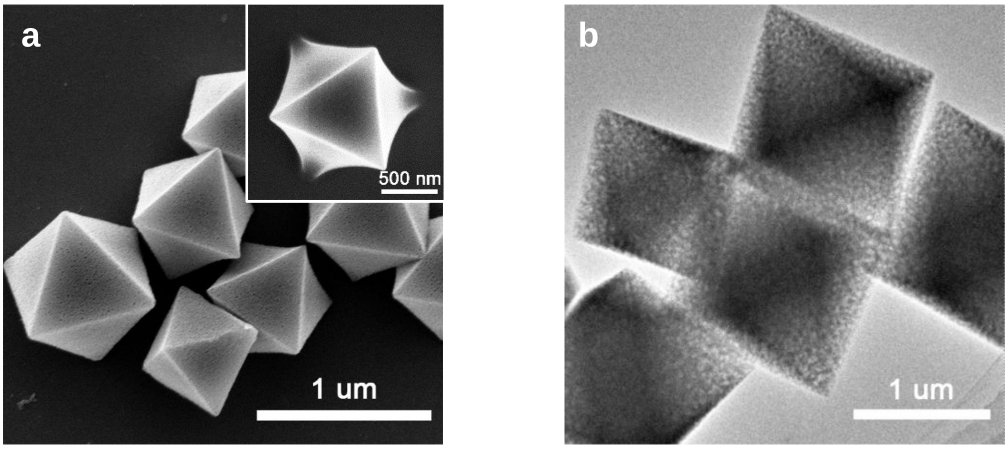

Supplementary Figure 5. The initial morphology characterization of the Cu-MOF precursors. SEM (a) and TEM (b) images of the Cu-MOF precursors.

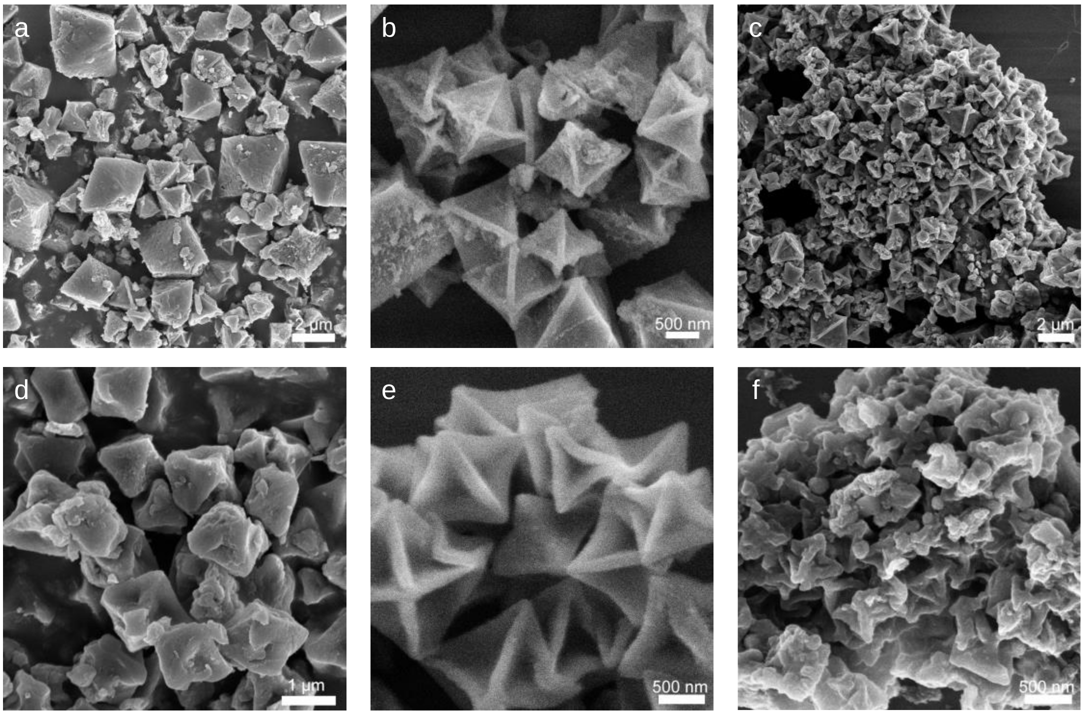

Supplementary Figure 6. Optimization of the TA exchange process. (a-c) Cu-TA obtained using 0.5, 1.0, and 2.0 mg ml^{-1} TA solutions, respectively. (d-f) Cu-TA obtained after etching times of 5, 7, and 9 h, respectively.

Supplementary Note 2. The optimization process is shown in Supplementary Figure 6. As a result, the Cu-MOF precursor could not be etched under a low TA concentration and short exchange time, while the parent octahedral morphology was broken under a high TA concentration and long exchange time. Thus, 1 mg/ml TA solution and a 7 h exchange time were chosen as the optimum conditions.

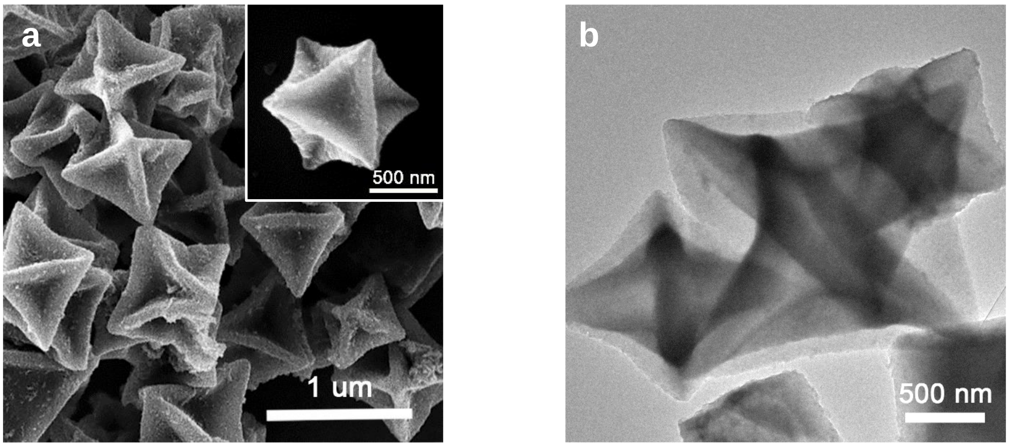

Supplementary Figure 7. Morphological characterization of Cu-TA. SEM (a) and TEM (b) images of the as-prepared Cu-TA (b).

Supplementary Note 3. As shown in the corresponding SEM and TEM images, the plate surface of the Cu-MOF precursors collapsed after etching by TA for 7 h due to the Kirkendall effect.

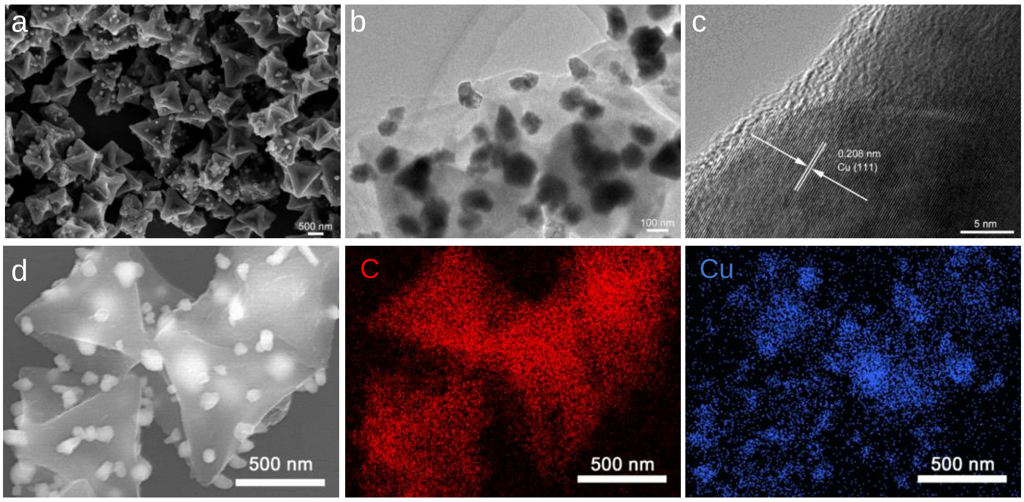

Supplementary Figure 8. SEM, TEM, and HRTEM images of Cu-PCC and the corresponding energy−dispersive X−ray spectroscopy (EDS) elemental mapping images. (a) SEM image. (b) TEM. (c) HRTEM image. (d) The corresponding image of Cu-PCC from scanning transmission electron microscopy under high-angle annular dark-field mode and energy−dispersive X−ray spectroscopy (EDS) elemental mapping images of Cu-PCC.

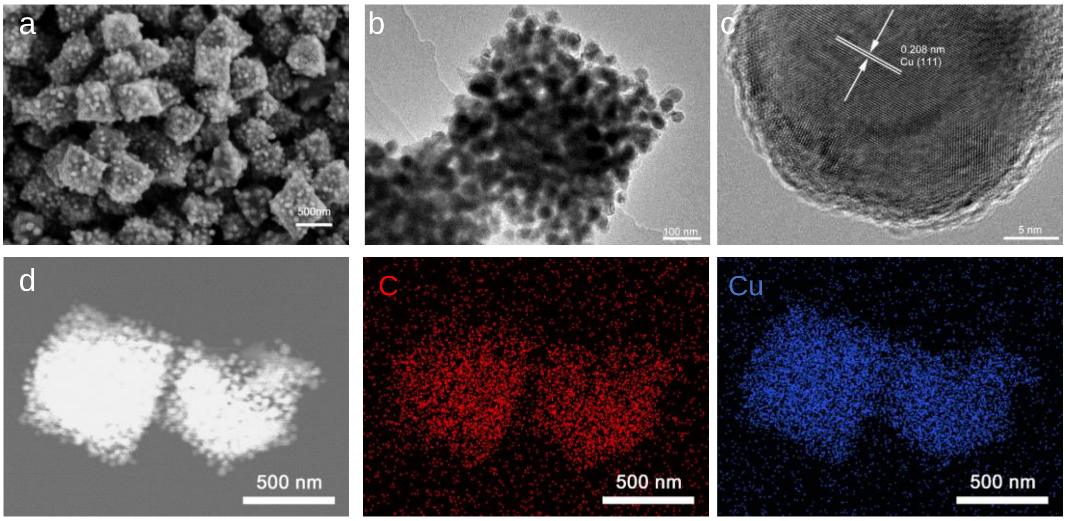

Supplementary Figure 9. SEM, TEM, and HRTEM images of Cu-C and the corresponding energy-dispersive X−ray spectroscopy (EDS) elemental mapping images. (a) SEM. (b) TEM. (c) HRTEM image. (d) The corresponding image of Cu-C from scanning transmission electron microscopy under high-angle annular dark-field mode and energy-dispersive X−ray spectroscopy (EDS) elemental mapping images of Cu-C.

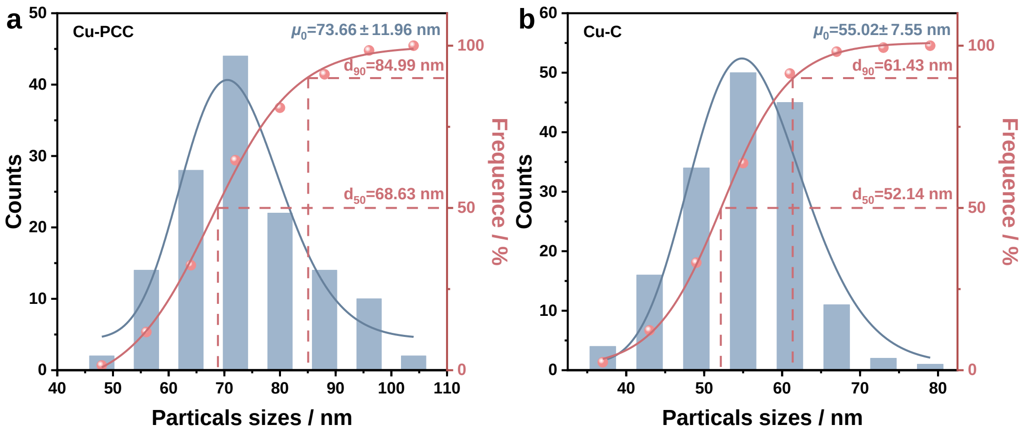

Supplementary Figure 10. The statistical size distributions of Cu-PCC (a) and Cu-C (b).

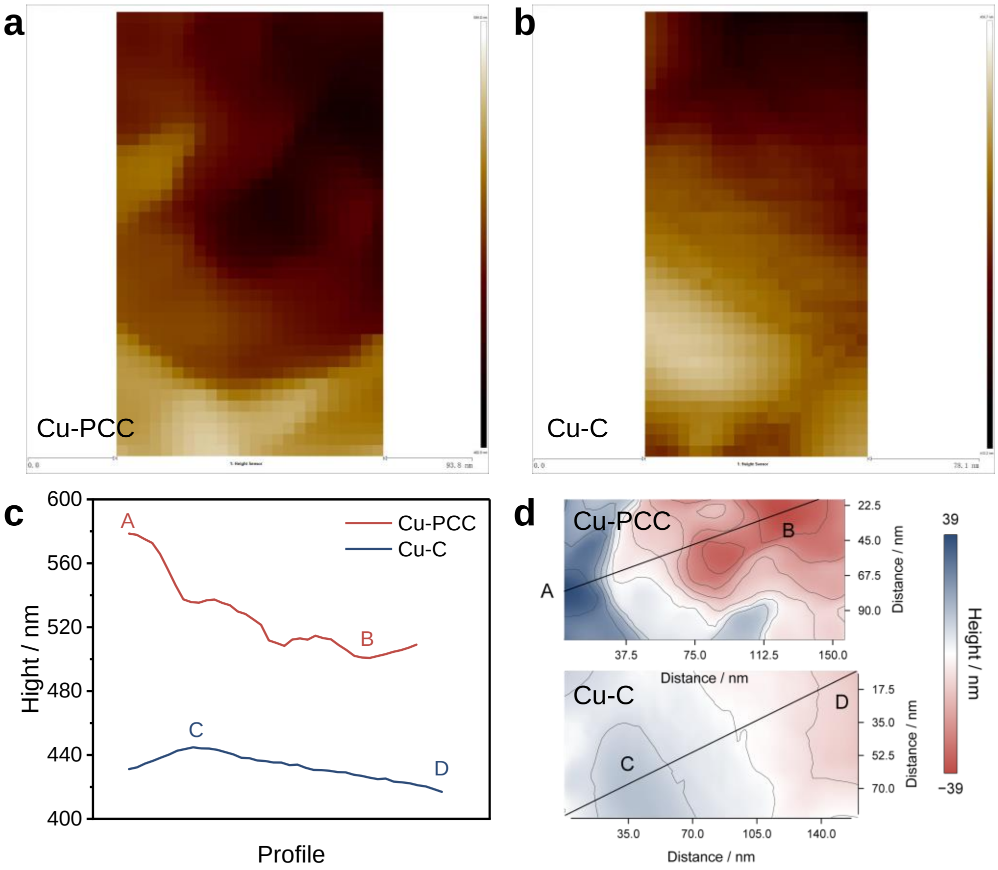

Supplementary Figure 11. AFM characterization. The original AFM images of Cu-PCC (a) and Cu-C (b); c, d) The height distribution of the cross profile between the lowest and highest points.

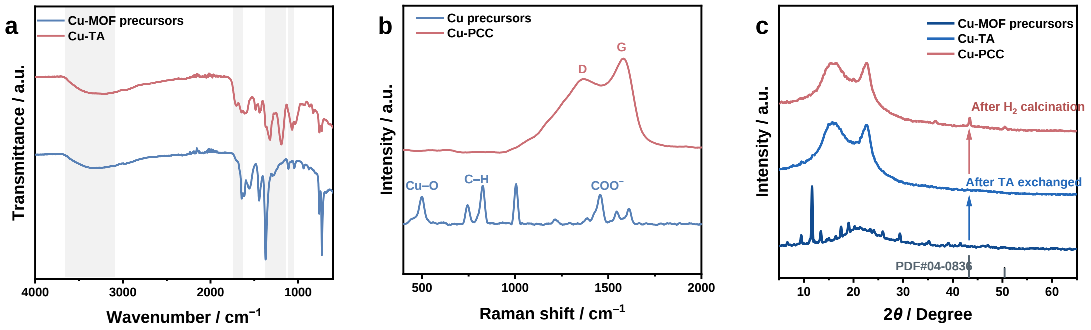

Supplementary Figure 12. The sequential conversion process from Cu-MOF precursors to Cu-PCC. (a) FTIR spectra of Cu-TA and Cu-MOF precursors. (b) Raman spectra of Cu-MOF and the as-prepared Cu-PCC. (c) XRD patterns of Cu-MOF, Cu-TA, and Cu-PCC.

Supplementary Note 4. According to the literature [1–3], the IR data suggest that all the peaks in Supplementary Figure 12a are attributed to the stretching vibration of functional groups from TA, indicating the substitution of H_3BTC by TA and the formation of Cu-TA complexes. As shown in Supplementary Figure 12c, the crystalline structure of the as-prepared Cu-MOF is similar to that reported in the literature [2], and these diffraction peaks disappear after the reaction of Cu-MOF with TA due to the amorphous character of the TA-metal complex. Furthermore, diffraction peaks attributed to metallic Cu appear after H_2 calcination, confirming the existence of metallic Cu.

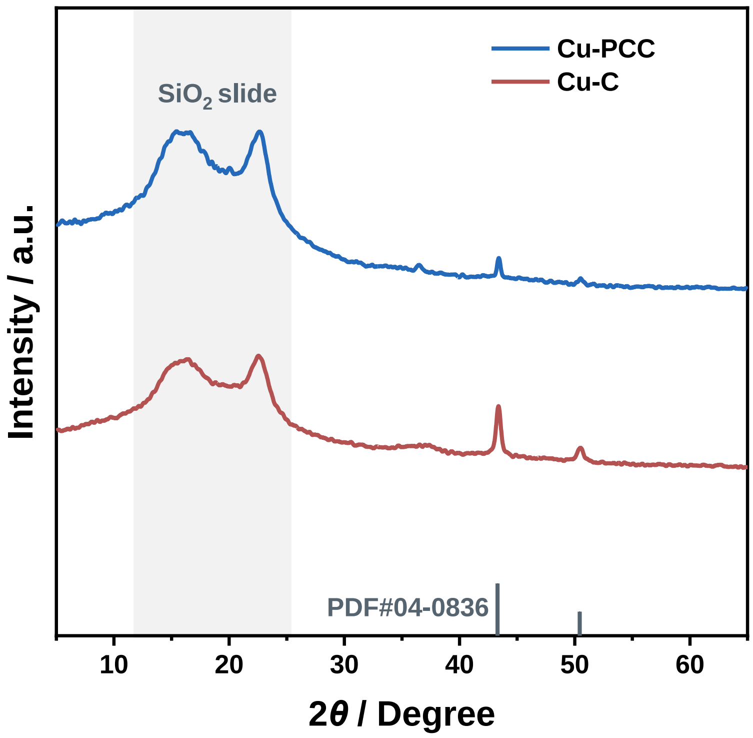

Supplementary Figure 13. Characterization of the samples calcinated under a H_2 atmosphere. XRD patterns of Cu-PCC and Cu-C.

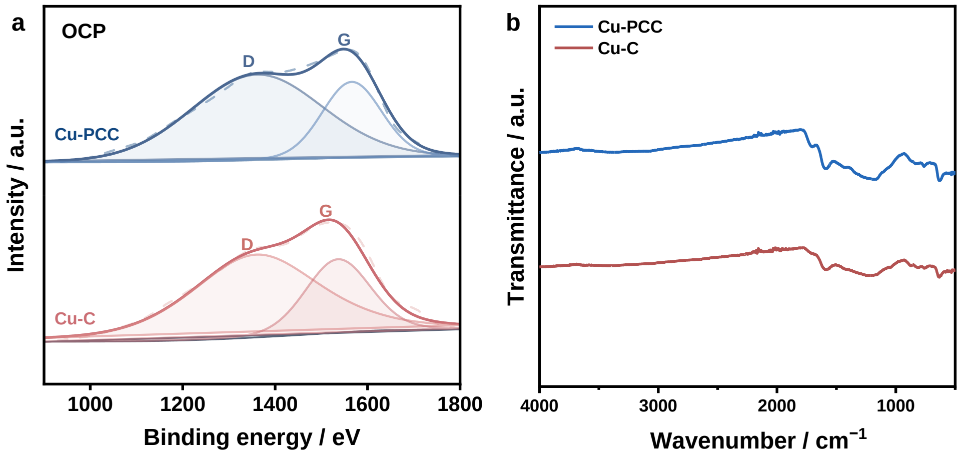

Supplementary Figure 14. Surface functional group characterization. Raman (a) and IR (b) spectra of Cu-PCC and Cu-C at OCP.

Supplementary Note 5. Generally, the relative ratio of the D to G band (I_D/I_G) is a descriptor of carbon defects, as shown in Supplementary Figure 14a. A similar I_D/I_G indicates that there is no obvious difference in the defect concentration between the PCC and C[4]. In addition, there is no difference in the oxygen-containing group content between PCC and C (Supplementary Figure 14b).

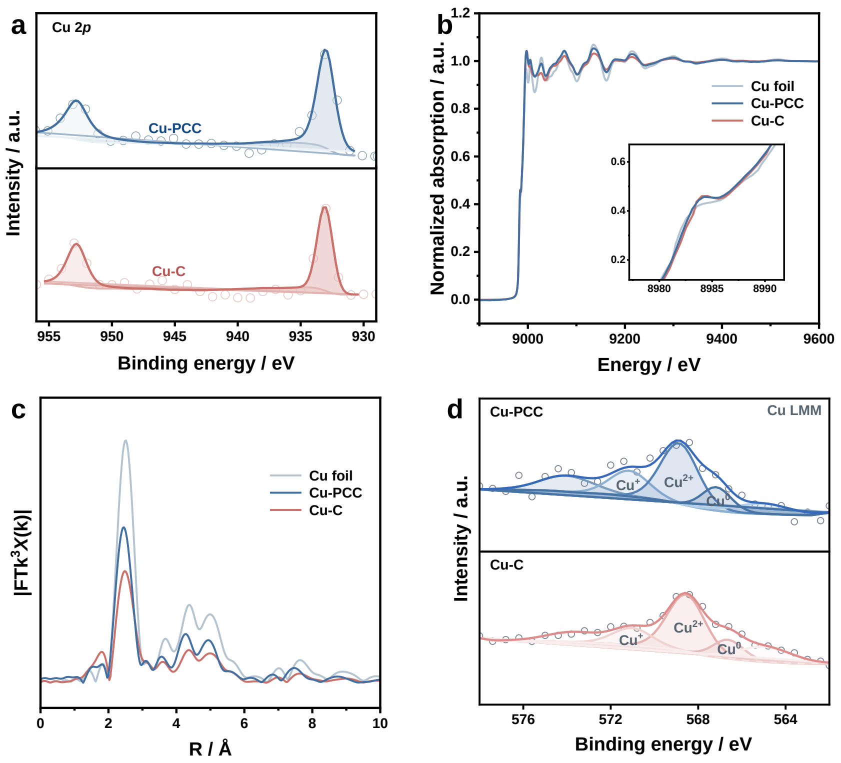

Supplementary Figure 15. The valence state and coordinate environment of Cu. (a) Cu 2*p* XPS, (b) X-ray absorption near-edge structure spectra, (c) EXAFS, and Cu Auger LMM spectra of Cu-PCC and Cu-C.

Supplementary Note 6. As shown in Supplementary Figure 15, the valence states and coordination environments of Cu in Cu-PCC and Cu-C are almost the same.

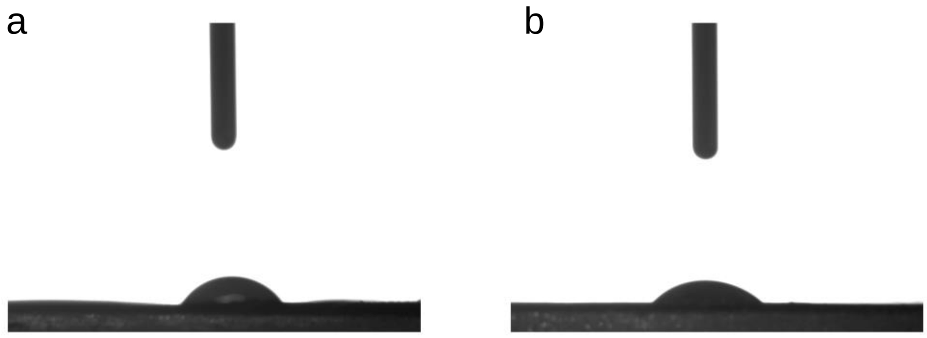

Supplementary Figure 16. Contact angle analysis of Cu-PCC (a) and Cu-C (b).

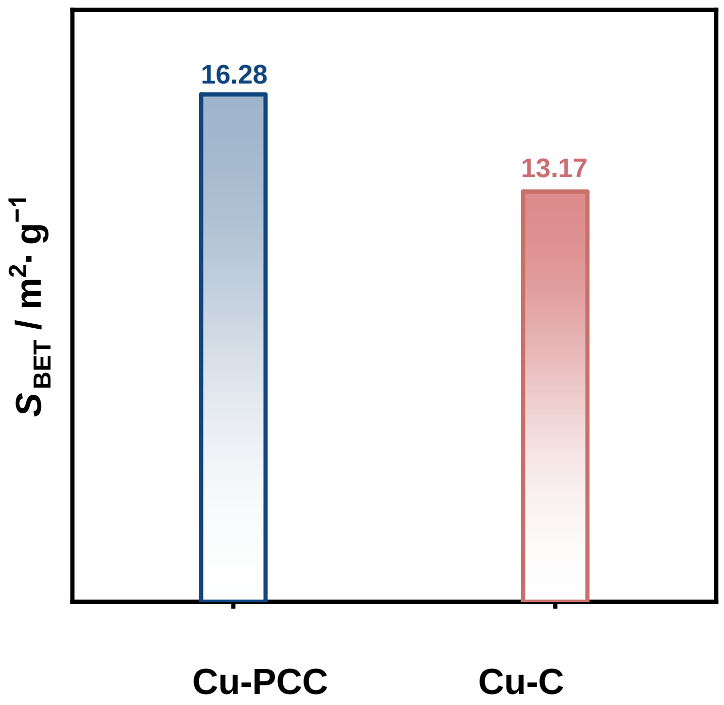

Supplementary Figure 17. Specific surface area analysis. Brunauer–Emmett–Teller surface areas of Cu-PCC and Cu-C.

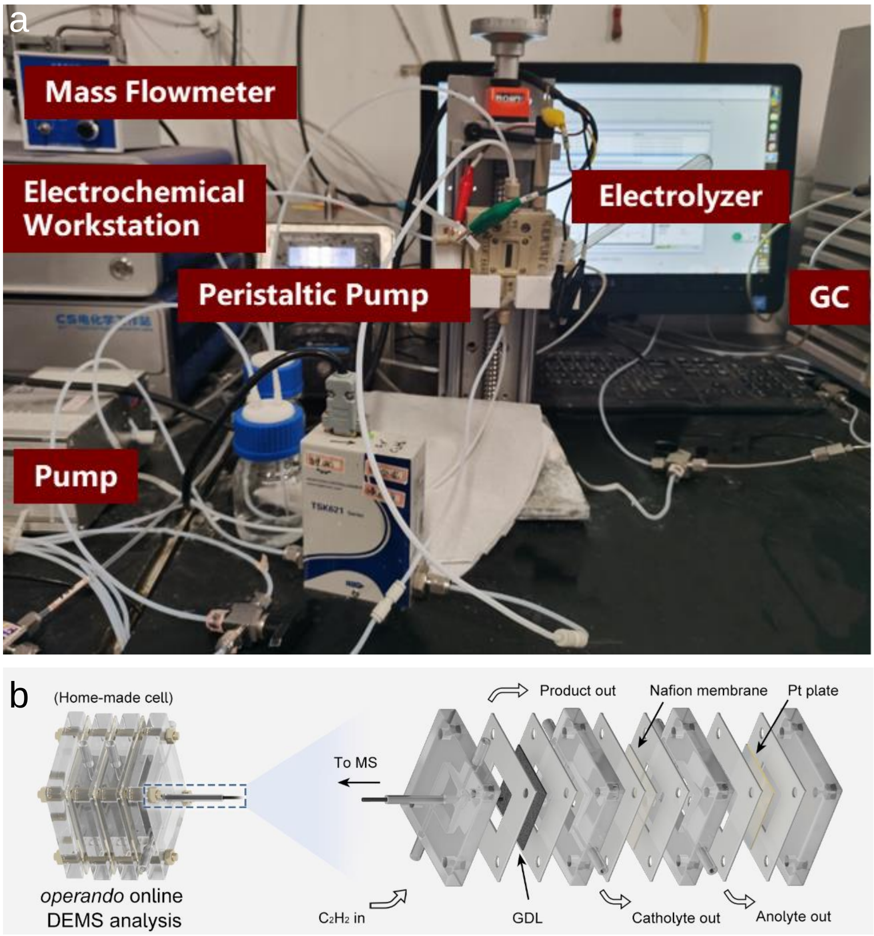

Supplementary Figure 18. Illustrations of the electrochemical setups. (a) Real picture of the ESAE process performance evaluation system. (b) Schematic illustration of the electrochemical cell used for DEMS.

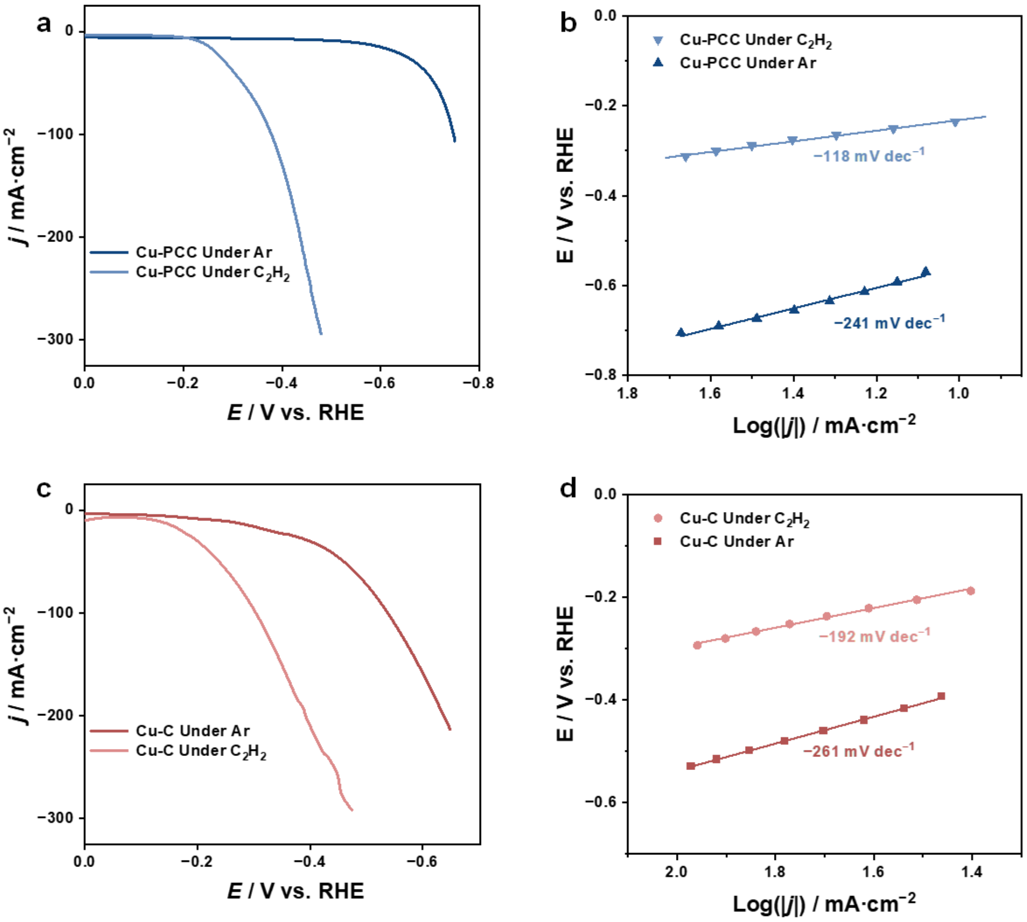

Supplementary Figure 19. The LSV curve and the corresponding Tafel slopes of Cu-PCC and Cu-C. The LSV curves of Cu-PCC under Ar and C_2H_2 atmospheres (a) and the corresponding Tafel slopes (b); the LSV curves of Cu-C under Ar and C_2H_2 atmospheres (c) and the corresponding Tafel slopes (d).

Supplementary Note 7. Supplementary Figure 19a,c clearly shows that the onset potential and current density across the whole potential range under a C_2H_2 atmosphere are much greater than those under an Ar atmosphere both over Cu-PCC and Cu-C. In addition, the difference in the absolute value of the Tafel slope of Cu-PCC (241-118=123 mV dec^{-1}) is much greater than that of Cu-C (261-192=69 mV dec^{-1}) (Supplementary Figure 19b,d), suggesting that the reaction kinetics of C_2H_2 semihydrogenation are enhanced over those of Cu-PCC due to the enrichment effect caused by the concave surface-induced field.

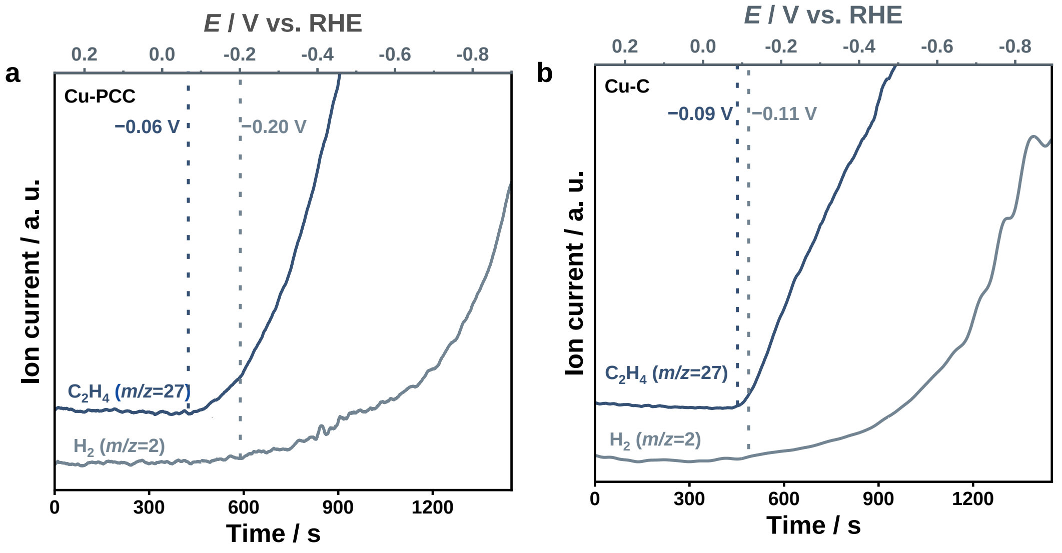

Supplementary Figure 20. Enlarged DEMS in LSV mode of Cu-PCC (a) and Cu-C (b).

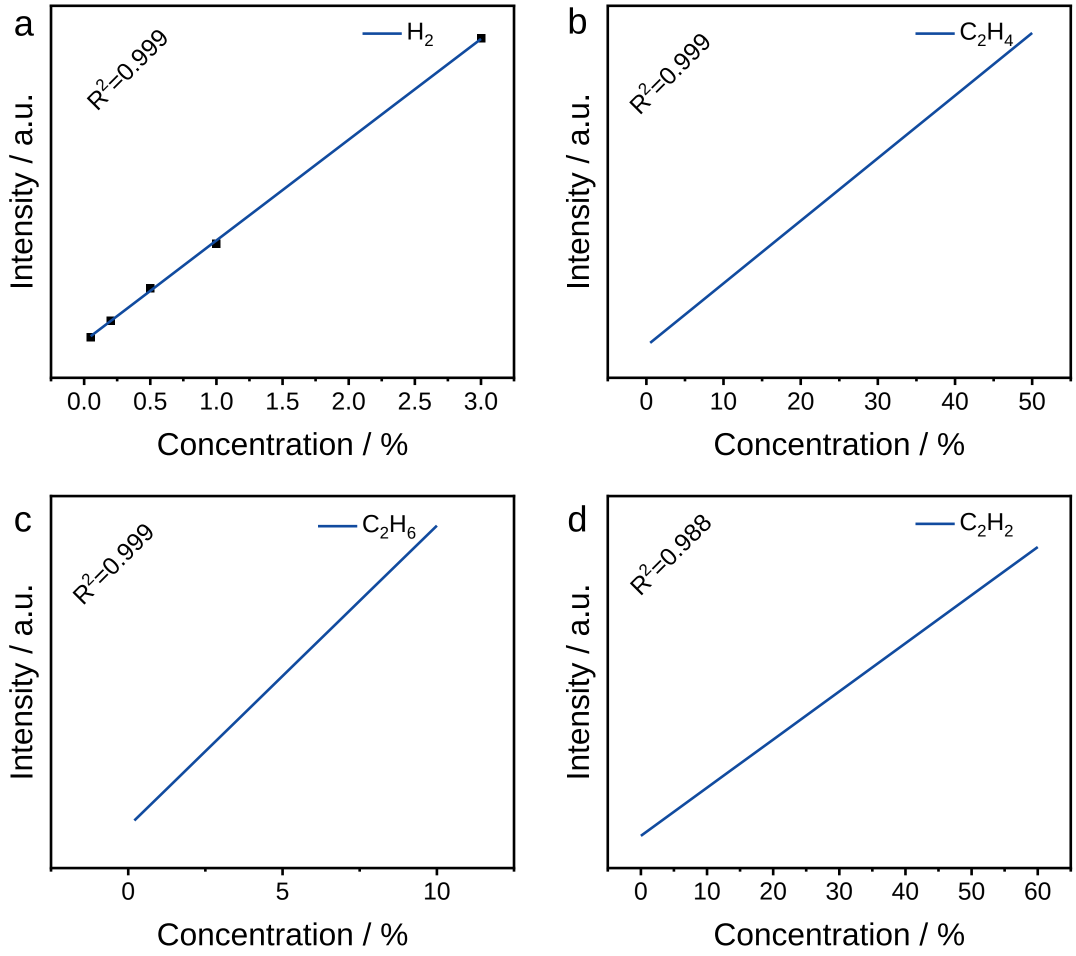

Supplementary Figure 21. The calibration curves used for product quantification. The corresponding standard curves of H_2 (a), C_2H_4 (b), C_2H_6 (c), and C_2H_2 (d) with different volume fractions for quantitation.

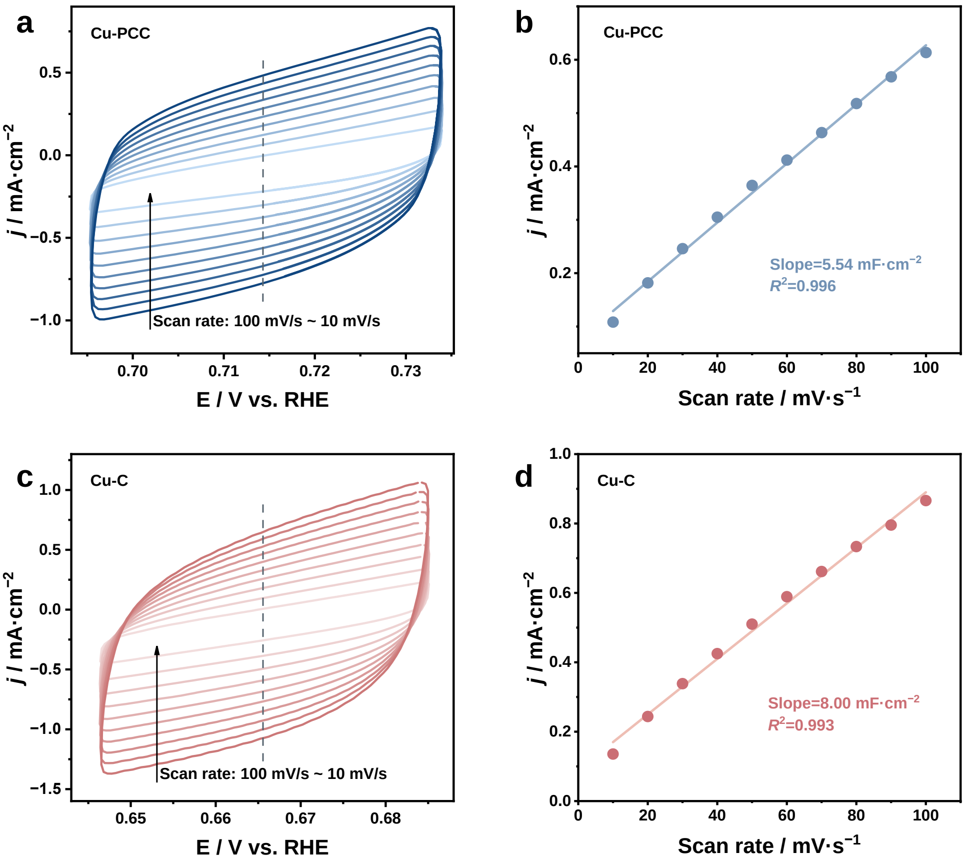

Supplementary Figure 22. Electrochemical capacitance measurements of Cu-PCC and Cu-C. Cyclic voltammogram curves of Cu-PCC at various scan rates (a) and the corresponding fitting (b); cyclic voltammogram curves of Cu-C at various scan rates (c) and the corresponding fitting (d).

Supplementary Note 8. Electrochemical capacitance measurements were used to determine the ECSA of the catalysts. To measure the electrochemical capacitance, the potential was swept at different scan rates ranging from 10-100 mV/s. The specific capacitance for a flat surface is generally found to be in the range of 20-60 μF cm^{-2}. In the calculations of the ECSA, we assumed a specific capacitance of 40 μF cm^{-2}.

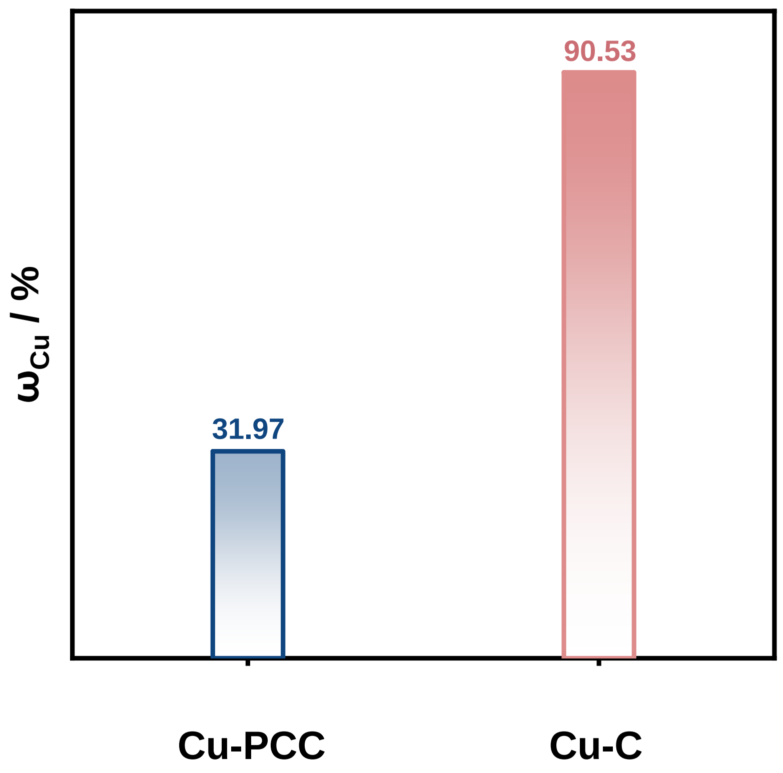

Supplementary Figure 23. ICP─OES analysis of the two catalysts. Mass fraction of Cu in Cu-PCC and Cu-C from the ICP─OES results.

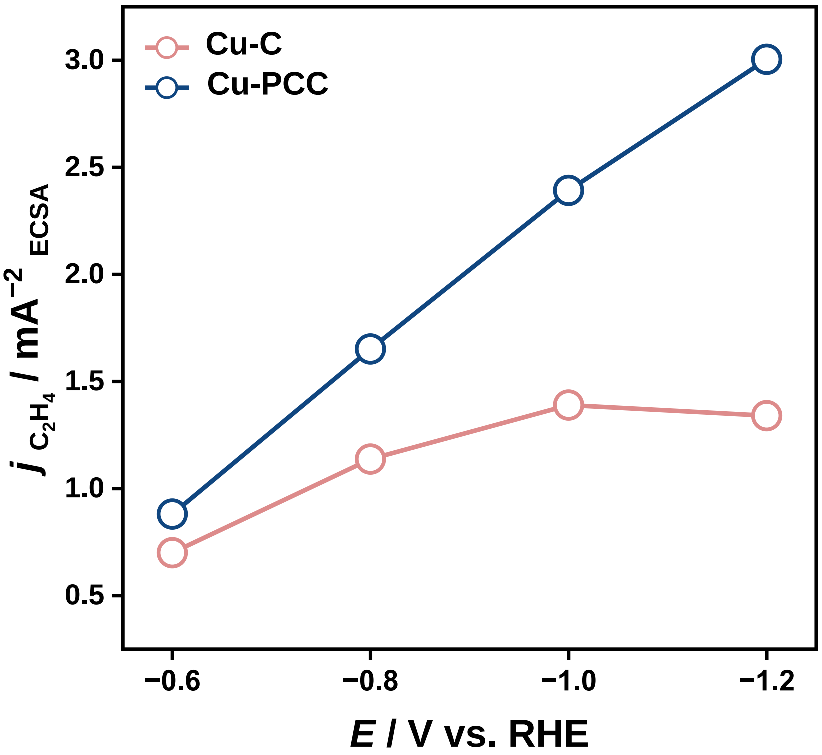

Supplementary Figure 24. Normalized performance comparison. The C_2H_4 partial current densities of Cu-PCC and Cu-C after normalization to the ECSA.

Supplementary Table 1 Comparison of the performance of the Cu-PCC and the state-of-the-art reports on ESAE.

Catalysis	c_{C2H2} / %	Electrolyte	j_{C2H4} / mA cm^{-2}	Potential	FE_{C2H4} / %	Specific selectivity / %	Reference
**Cu-PCC**	**15**	**1 M KOH**	**-420**	**-1.2 V vs. RHE**	**91.53**	**100**	**This work**
Ag NWs	1	1 M KOH	-2.2	/	/	100	*CCS Chem.* 2023, 5, 200-208
LD-Cu	5	1 M KOH	-61.9	-0.6 V vs. RHE	74.9	~92	*Nat. Catal.* 2021, 4,565-574
ED-Cu NPs	100	1 M KOH	-488.7	-1.93 V vs. RHE	97.7	100	*Nat. Sustain.* 2023, 6, 827-837
Cu-MPs	/	1 M KOH	~-26	-0.9 V vs. RHE	~40	~80	*Nat. Commun.* 2021, 12, 7072
Cu dendrites	100	1 M KOH	-150	-0.8 V vs. RHE	~93	~98	*Nat. Catal.* 2021, 4, 557-564
NHC-Cu	100	1 M KOH	-158.8	-0.9 V vs. RHE	98	96	*Nat. Commun.* 2021, 12, 6574
2TIm		1 M KOH	-225	-0.9 V vs. RHE	98	98	*Nat. Chem.* 2024, 10.1038/s41557-024-01480-6

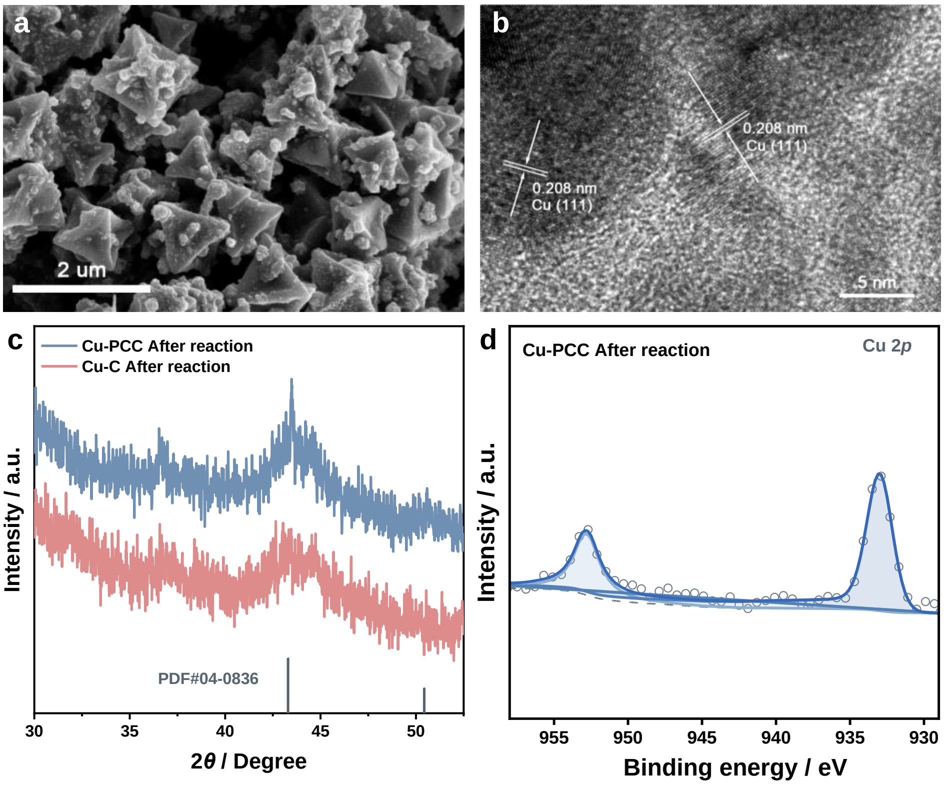

Supplementary Figure 25. Stability of Cu-PCC after 12 h of continuous testing. (a) SEM image, (b) HRTEM image, (c) XRD pattern, and Cu 2p XPS spectrum (d).

Supplementary Note 9. The SEM images after 12 h of continuous testing indicated that the concave surface and particle size of the Cu-PCC could be maintained. The HRTEM images and XRD patterns confirmed that the crystal phase was unchanged, suggesting the robust stability of the as-prepared catalysts. In addition, the binding energy of Cu remains unchanged after the stability test, indicating that the metallic Cu site is the true active site of the ESAE process.

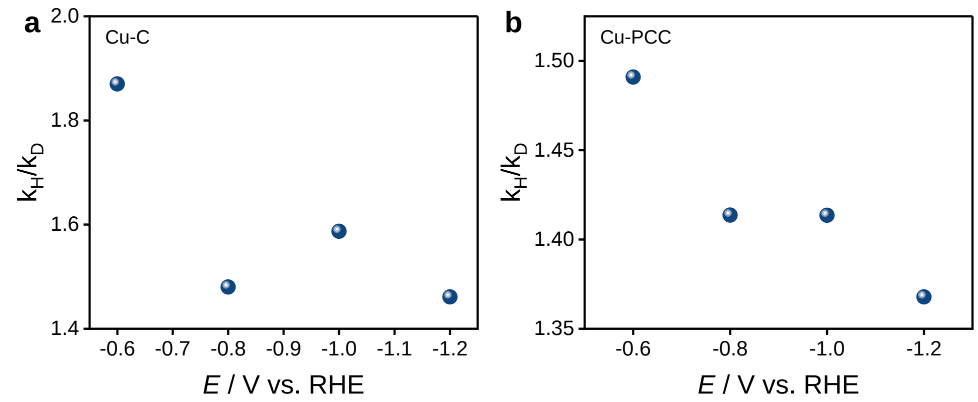

Supplementary Figure 26. Kinetic isotope effect comparison. The KIE values of Cu-PCC (a) and Cu-C (b) under different applied potentials.

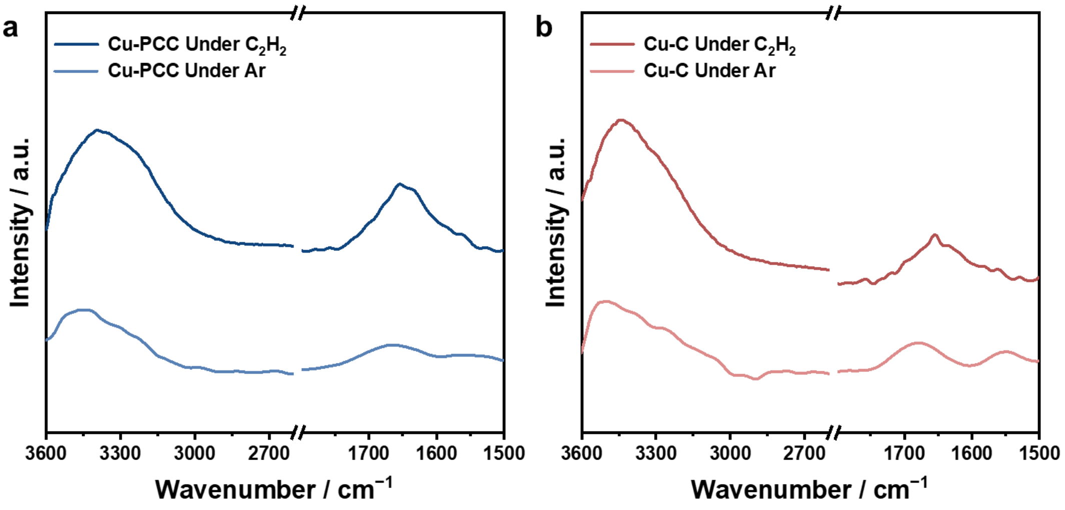

Supplementary Figure 27. The recognition of characteristic peaks of H_2O and C_2H_2. The ATR-FTIR spectra of Cu-PCC (a) and Cu-C (b) at OCP under Ar and C_2H_2 atmospheres, respectively.

Supplementary Note 10. As shown in Supplementary Figure 27, the peaks located at 3600~3000 cm^{-1} and 1750~1550 cm^{-1} under C_2H_2 are much wider than the corresponding characteristic water peaks under Ar over both Cu-PCC and Cu-C, indicating that all the peaks under C_2H_2 are attributed to water and C_2H_2. Thus, the relative coverage of water and C_2H_2 could be obtained through peak differentiation and fitting analysis.

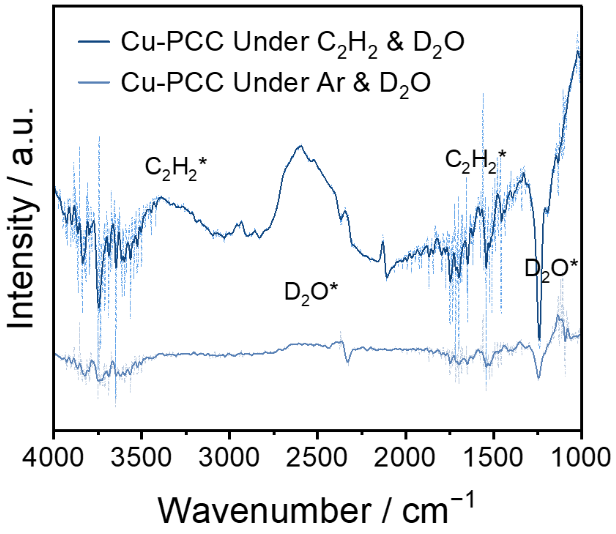

Supplementary Figure 28. Further recognition of C_2H_2 peaks was performed using the isotope effect. IR results obtained using D_2O under C_2H_2 and Ar atmospheres.

Supplementary Note 11. As shown in Supplementary Figure 28, to eliminate the interference of the O-H bond in H_2O when identifying the location of C_2H_2*, D_2O was used to replace H_2O. The peaks located at approximately 3200 to 3300 cm^{-1} and approximately 1600 cm^{-1} could only be detected under a C_2H_2 atmosphere. Thus, these peaks were attributed to ν(C−H) and ν(C≡C), respectively.

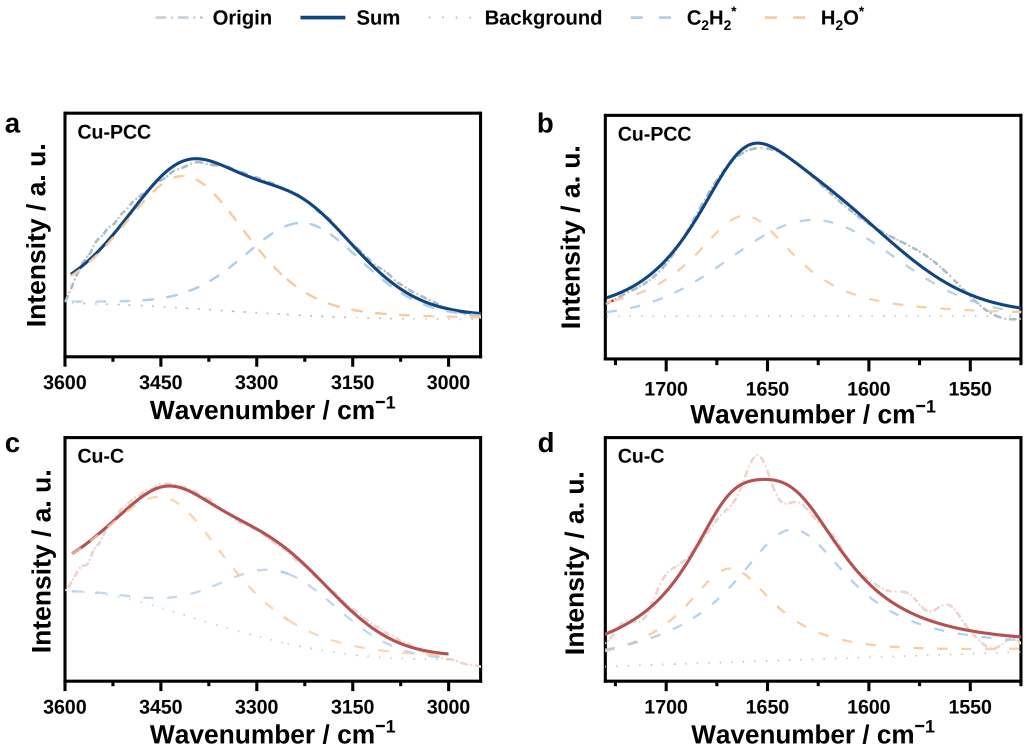

Supplementary Figure 29. Peak-differentiating and fitting analysis of the ATR-FTIR data The corresponding results of Cu-PCC (a, b) and Cu-C (c, d) under a C_2H_2 atmosphere.

Supplementary Note 12. As shown in Supplementary Figure 29, the peak located at 3600~3000 cm^{-1} at the OCP under a C_2H_2 atmosphere could be divided into two peaks located at ~3410 and ~3200 cm^{-1}, which could be attributed to the *v*(O-H) of water and the *v*(C-H) of C_2H_2, respectively, both over Cu-PCC and Cu-C. In addition, the peaks in the range of 1750~1550 cm^{-1} could also be attributed to δ (H-O-H) (~1660 cm^{-1}) and *v*(C≡C) (~1620 cm^{-1}) [5,6]. Note that both the characteristic peaks of *v*(C-H) and *v*(C≡C) exhibit a redshift over Cu-PCC compared to their Cu-C counterparts, indicating that the carbon–carbon bonding of C_2H_2 over Cu-PCC has been weakened due to the delocalization of π electrons.

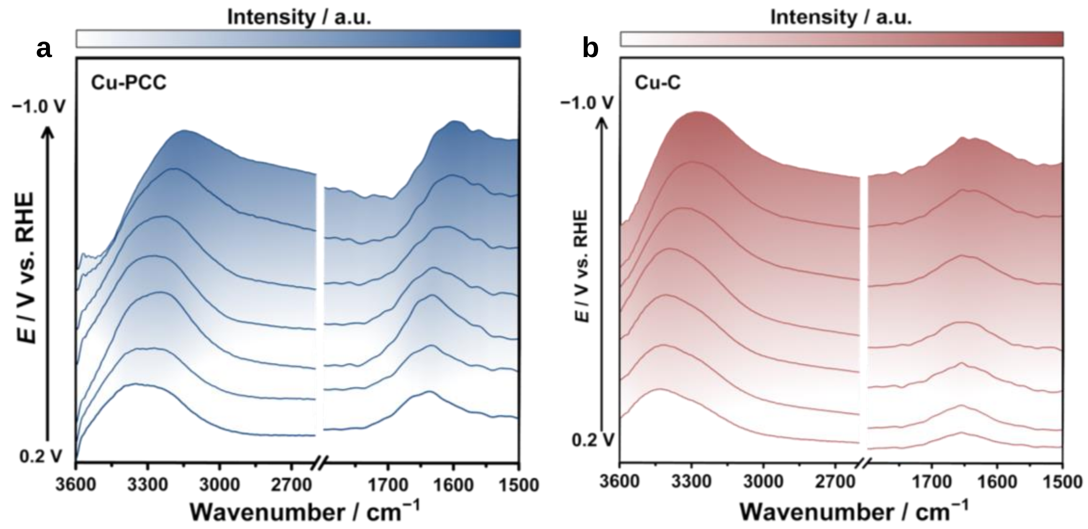

Supplementary Figure 30. The results of ATR−FTIR. Potential-dependent *in situ* ATR-FTIR results under a C_2H_2 atmosphere over Cu-PCC (a) and Cu-C (b).

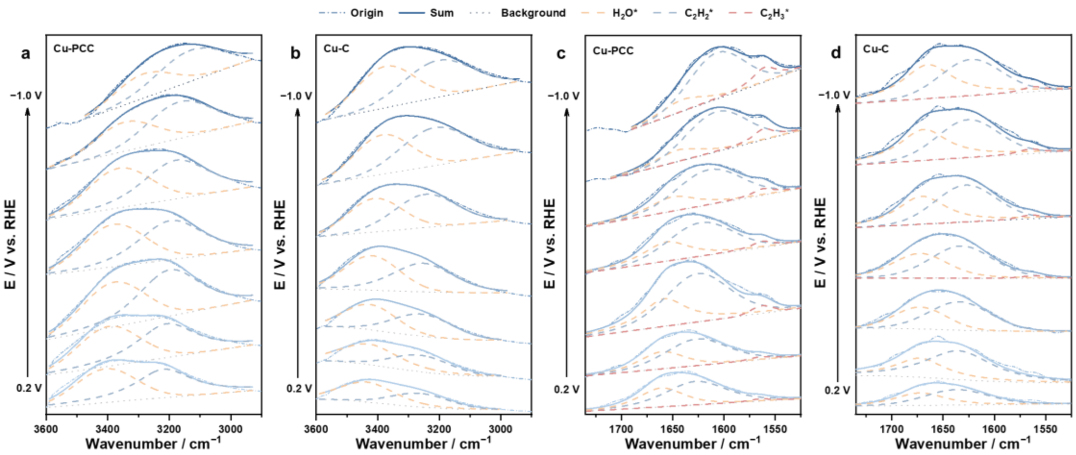

Supplementary Figure 31. Peak-differentiating and fitting analysis. The potential-dependent *in situ* ATR-FTIR data of Cu-PCC (a,c) and Cu-C (b,d) under a C_2H_2 atmosphere.

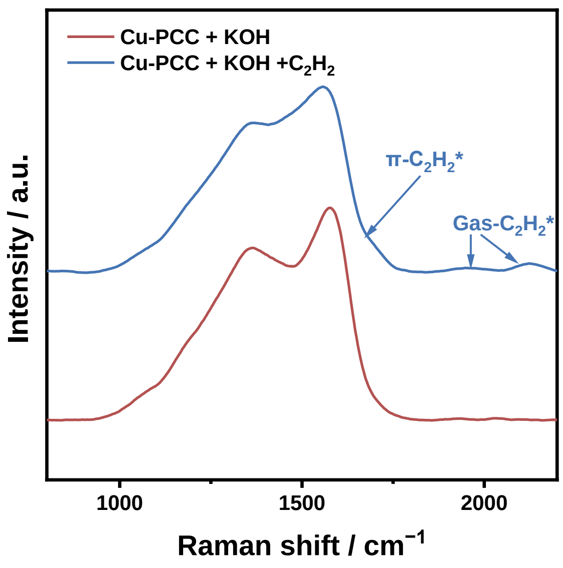

Supplementary Figure 32. Recognition of the Raman characteristic peaks of C_2H_2. Raman spectra of Cu-PCC and Cu-C at OCP under Ar and C_2H_2 atmospheres, respectively.

Supplementary Note 13. As shown in Supplementary Figure 32, the peak located in the range of 1650~1750 cm^{-1} corresponds to gaseous C_2H_2 at approximately 1800 to 2100 cm^{-1} under a C_2H_2 atmosphere, whereas no peak appears under an Ar atmosphere. The above results verify that the peak located at approximately 1650~1750 cm^{-1} is attributed to adsorbed C_2H_2.

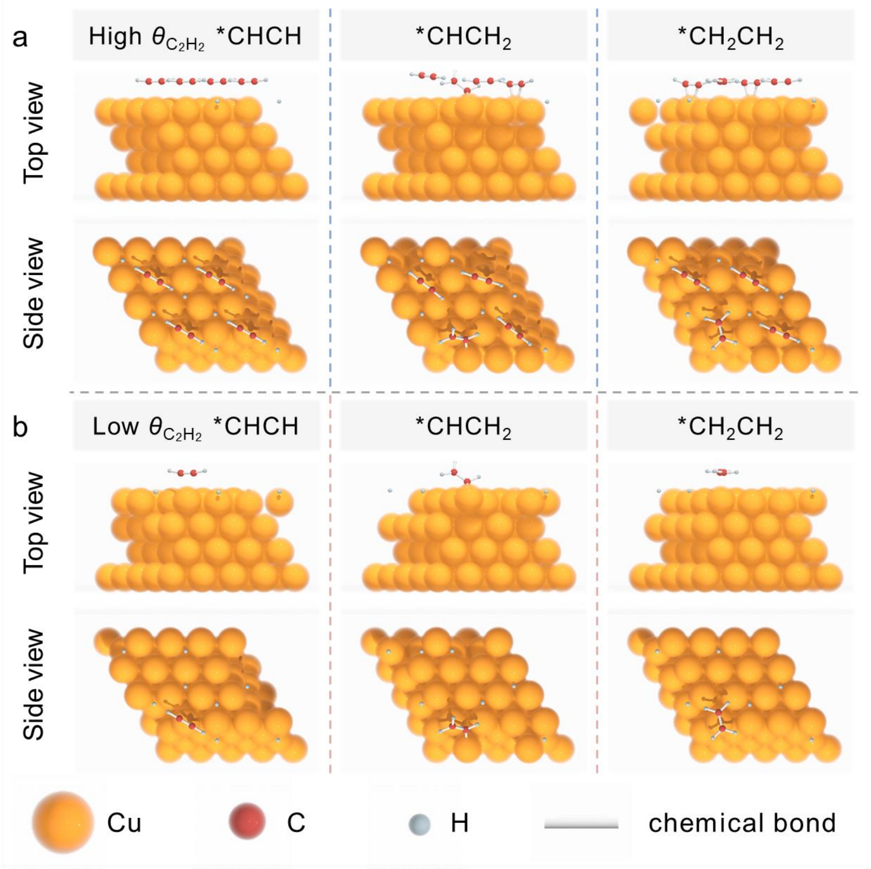

Supplementary Figure 33. The optimized structures used for theoretical calculations. Theoretical adsorption models of the hydrogenation process of C_2H_2 over high C_2H_2 coverage and low coverage.

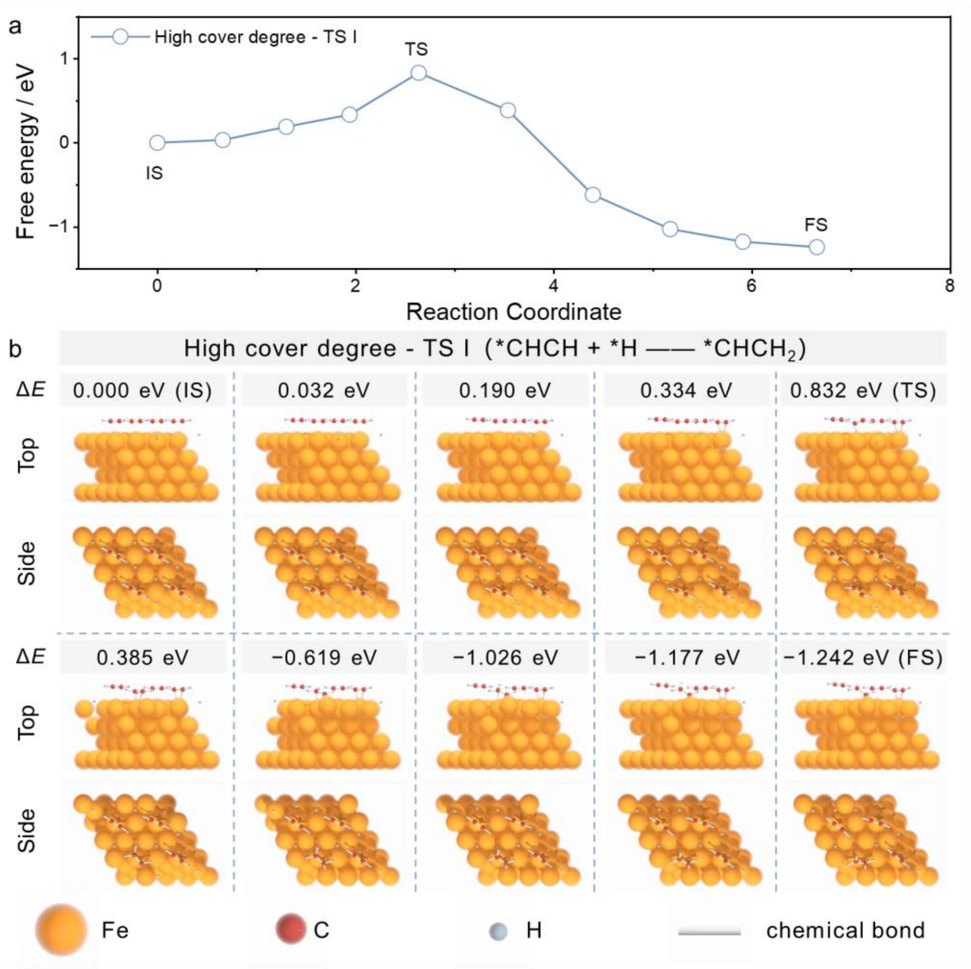

Supplementary Figure 34. Energy profiles and optimized adsorption structures from C_2H_2 to C_2H_3 with high C_2H_2 coverage. The energy change profiles (a) and theoretical adsorption models (b) of the transition states for C_2H_2 hydrogenation to C_2H_3 with high C_2H_2 coverage.

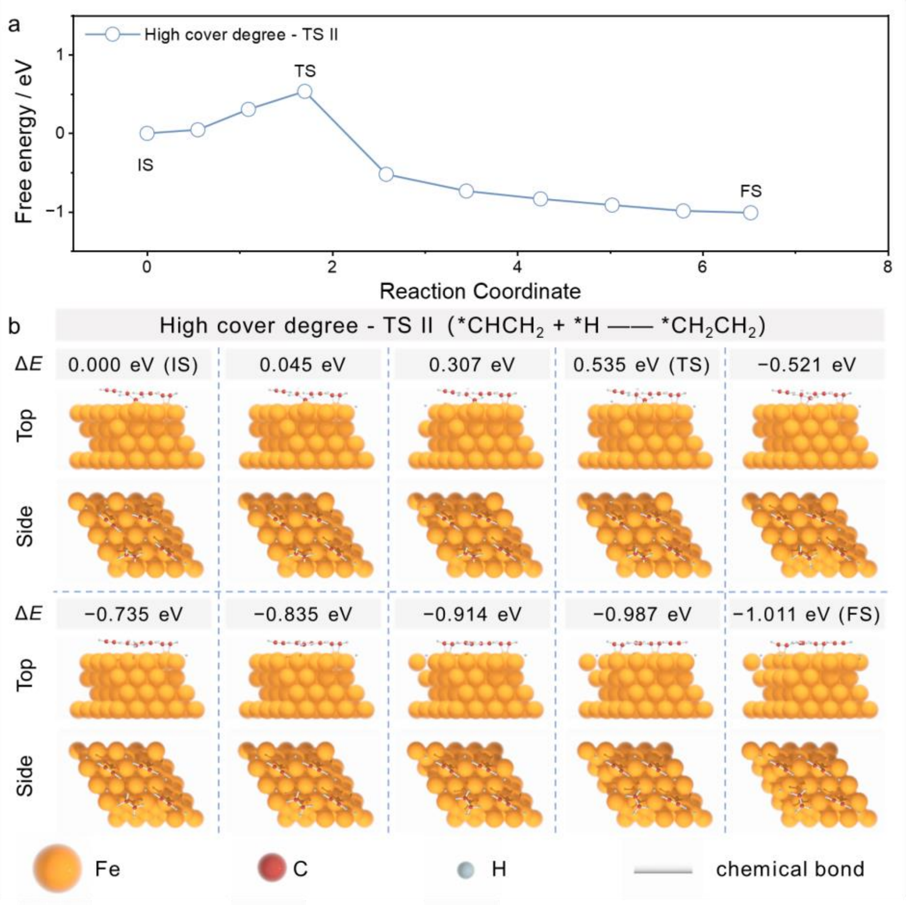

Supplementary Figure 35. Energy profiles and optimized adsorption structures from C_2H_3 to C_2H_4 with high C_2H_2 coverage. The energy change profiles (a) and theoretical adsorption models (b) of the transition states for C_2H_3 hydrogenation to C_2H_4 with high C_2H_2 coverage.

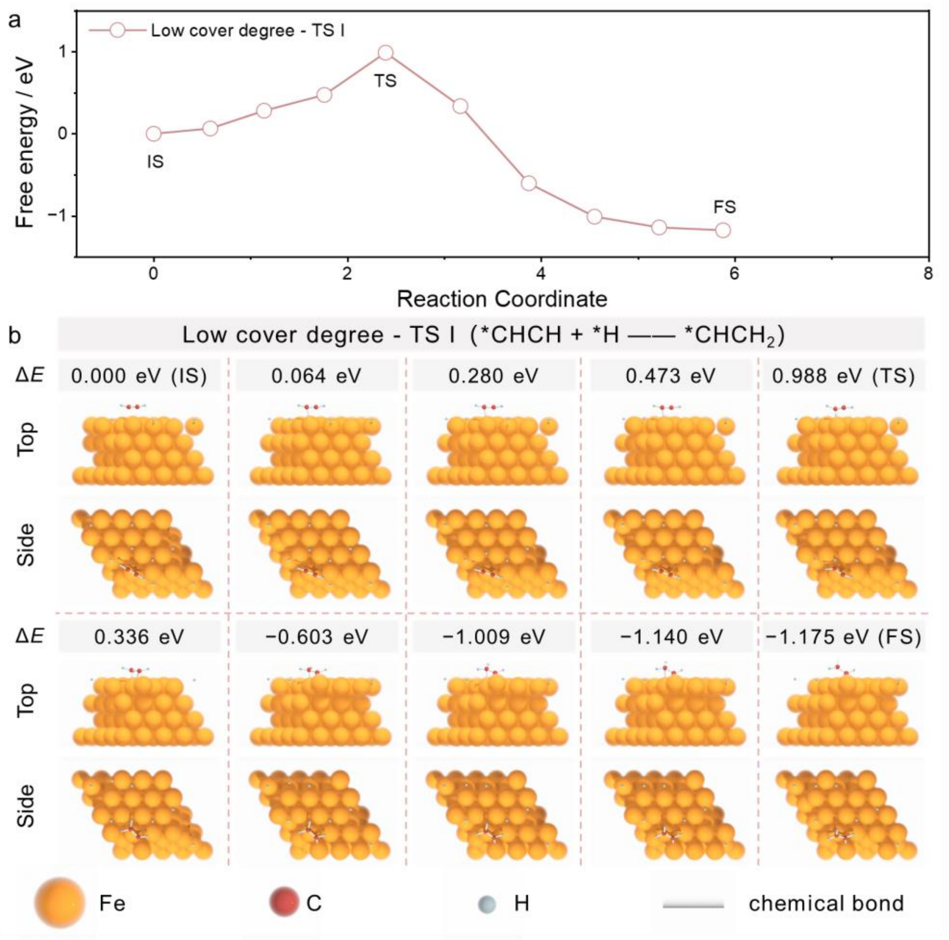

Supplementary Figure 36. Energy profiles and optimized adsorption structures from C_2H_2 to C_2H_3 with low C_2H_2 coverage. The energy change profiles (a) and theoretical adsorption models (b) of the transition states for C_2H_2 hydrogenation to C_2H_3 with low C_2H_2 coverage.

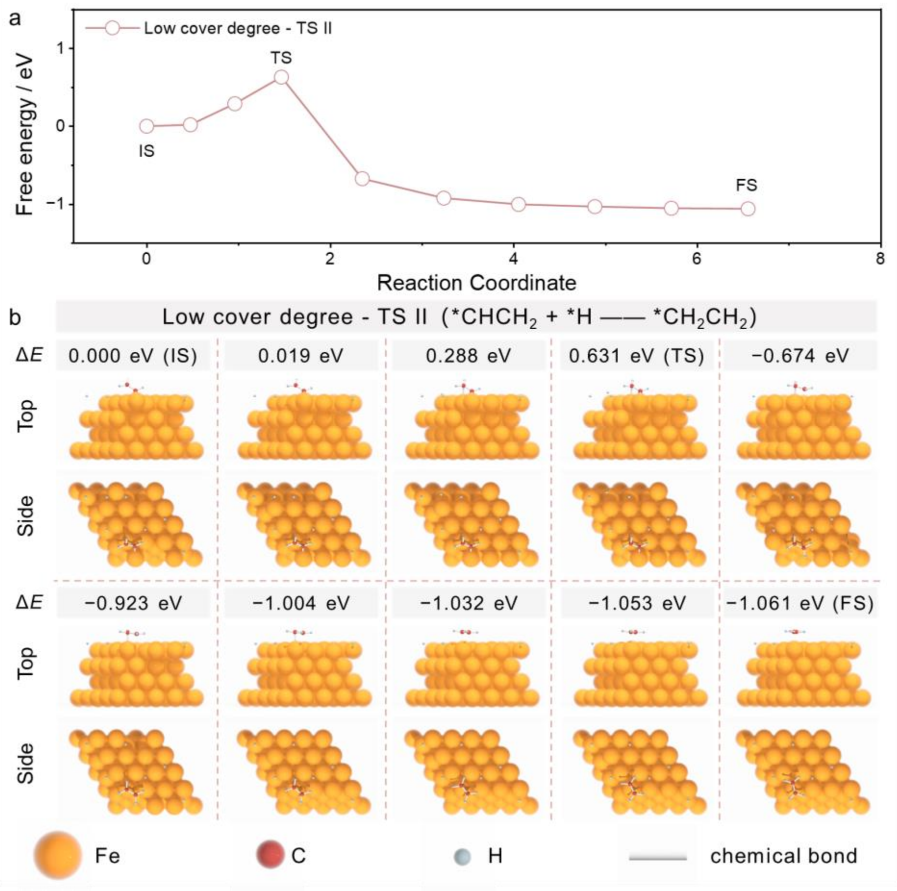

Supplementary Figure 37. Energy profiles and optimized adsorption structures from C_2H_3 to C_2H_4 with low C_2H_2 coverage. The energy change profiles (a) and theoretical adsorption models (b) of the transition states for C_2H_3 hydrogenation to C_2H_4 with low C_2H_2 coverage.

Supplementary References

1. Shi, Y. M., Yu, Y., Liang, Y., Du, Y. H. & Zhang, B. In situ electrochemical conversion of an ultrathin tannin nickel Iron complex film as an efficient oxygen evolution reaction electrocatalyst. *Angew. Chem. Int. Ed.* **58**, 3769-3773, (2019).
2. Wang, M. K. *et al.* Boosting CO_2-to-CO selectivity and durability by metal-support interaction and encapsulated effect of Ni@C capsules. *Chem. Eng. J.* **454**, 140000, (2023).
3. Shi, Y. M. *et al.* Unveiling hydrocerussite as an electrochemically stable active phase for efficient carbon dioxide electroreduction to formate. *Nat. Commun.* **11**, 3415, (2020).
4. Liu, H. *et al.* Curving effects of concave dodecahedral nanocarbons enable enhanced Li-ion storage. *J. Mater. Chem. A* **6**, 14894-14902, (2018).
5. Ma, C. Y. *et al.* Mesoporous Co_3O_4 and Au/Co_3O_4 catalysts for low-temperature oxidation of trace ethylene. *J. Am. Chem. Soc.* **132**, 2608-2613, (2010).
6. Gu, J. *et al.* Synergizing metal–support interactions and spatial confinement boosts dynamics of atomic nickel for hydrogenations. *Nat. Nanotechnol.* **16**, 1141-1149, (2021).
