# Supplementary material for: Ethylene electrosynthesis from low-concentrated acetylene via concave-surface enriched reactant and improved mass transfer
Source: Nat Commun. 2024 Jul 13;15:5914. doi: 10.1038/s41467-024-50335-8 (PMC11246534; doi:10.1038/s41467-024-50335-8)
Supplement: Supplementary file 3 — Description of Additional Supplementary Files [file 41467_2024_50335_MOESM3_ESM.pdf]

# Description of Additional Supplementary Files

**Supplementary Data 1: The optimized DFT computational models.** Atomic coordinates of carbon models with and without concave surface in AIMD and DFT processes.
