# Supplementary material for: Ethylene electrosynthesis from low-concentrated acetylene via concave-surface enriched reactant and improved mass transfer
Source: Nat Commun. 2024 Jul 13;15:5914. doi: 10.1038/s41467-024-50335-8 (PMC11246534; doi:10.1038/s41467-024-50335-8)
Supplement: Supplementary file 4 — Supplementary Data 1 [file 41467_2024_50335_MOESM4_ESM.pdf]

```
Initial state of AIMD in C model
1.0
        9.8709001541         0.0000000000         0.0000000000
        0.0000000000        17.0969009399         0.0000000000
        0.0000000000         0.0000000000        20.0000000000
    C    H    O
   64  228  114
Direct
     0.726949973         0.980620056         0.210000000
     0.476660008         0.980659995         0.210000000
     0.607779959         0.772070016         0.210000000
     0.482300037         0.647360019         0.210000000
     0.361730004         0.520600031         0.210000000
     0.227259985         0.980899963         0.210000000
     0.225169991         0.396829992         0.210000000
     0.100620004         0.272199984         0.210000000
     0.975849990         0.147259990         0.210000000
     0.976780003         0.980079989         0.210000000
     0.479879983         0.897130036         0.210000000
     0.358150017         0.772920000         0.210000000
     0.233090008         0.647730011         0.210000000
     0.104690002         0.522350034         0.210000000
     0.978289995         0.480980008         0.210000000
     0.976149979         0.398230000         0.210000000
     0.231539993         0.898469996         0.210000000
     0.851519977         0.021550000         0.210000000
     0.107059998         0.857140020         0.210000000
     0.107550004         0.773190033         0.210000000
     0.981009989         0.647539967         0.210000000
     0.850389971         0.271860002         0.210000000
     0.980730000         0.897230050         0.210000000
     0.851099992         0.105509994         0.210000000
     0.850020033         0.356050013         0.210000000
     0.725929963         0.229859991         0.210000000
     0.725870013         0.146620006         0.210000000
     0.982299989         0.731649988         0.210000000
     0.600220001         0.105619994         0.210000000
     0.856170043         0.605139953         0.210000000
     0.601310009         0.022089999         0.210000000
     0.730539983         0.897019981         0.210000000
     0.729420025         0.479929995         0.210000000
     0.599730020         0.354289997         0.210000000
     0.475849990         0.230169991         0.210000000
     0.350479992         0.105830000         0.210000000
```

0.856350037	0.855530027	0.210000000
0.732540004	0.730939958	0.210000000
0.101470003	0.020900000	0.210000000
0.606549957	0.607309984	0.210000000
0.466140017	0.479990015	0.210000000
0.853619994	0.521539989	0.210000000
0.226110004	0.147389987	0.210000000
0.352710004	0.354920011	0.210000000
0.226379994	0.230979994	0.210000000
0.726320045	0.397749980	0.210000000
0.351810014	0.272060003	0.210000000
0.101190001	0.105159999	0.210000000
0.605449998	0.855359952	0.210000000
0.483100007	0.730920044	0.210000000
0.357070010	0.607220010	0.210000000
0.475160016	0.394259988	0.210000000
0.226329996	0.479440019	0.210000000
0.100420000	0.355899991	0.210000000
0.600950022	0.271050013	0.210000000
0.975070019	0.231089994	0.210000000
0.355910005	0.856400036	0.210000000
0.606850042	0.522239979	0.210000000
0.233509992	0.731930007	0.210000000
0.105189996	0.606890012	0.210000000
0.474659986	0.146909995	0.210000000
0.350989997	0.022210000	0.210000000
0.857769983	0.772199984	0.210000000
0.730889969	0.647690016	0.210000000
0.679119983	0.993450026	0.007650000
0.088799996	0.770489976	0.013679999
0.813220064	0.831570055	0.930010033
0.144769994	0.592109955	0.967100048
0.953700001	0.925259993	0.909819984
0.024800001	0.052530003	0.955420017
0.425430001	0.232649994	0.969149971
0.693870008	0.360310000	0.915690041
0.114469997	0.169750006	0.992490005
0.953400012	0.154119992	0.979730034
0.771040006	0.082089999	0.889940071
0.367980010	0.006920000	0.997480011
0.039009998	0.336829992	0.951370049
0.243819991	0.383909993	0.861940002
0.423299984	0.557070016	0.914770031
0.764869997	0.171839991	0.905019951

0.461959980	0.073820005	0.963460064
0.242210003	0.627260004	0.911950016
0.799049968	0.558979997	0.873430061
0.136720006	0.513159967	0.871330070
0.260740007	0.500069949	0.826879978
0.324030020	0.038660002	0.911330032
0.851179989	0.897279974	0.792399979
0.566030030	0.278039983	0.873649979
0.985530012	0.269279985	0.830389977
0.204669989	0.862190000	0.958689976
0.573260000	0.813350012	0.942659950
0.527169993	0.966139987	0.861140060
0.764169975	0.686130002	0.810700035
0.498050025	0.534529993	0.848760033
0.844019969	0.279430007	0.794640017
0.221570006	0.894609982	0.885379982
0.698539978	0.923589980	0.792409992
0.596210007	0.274349989	0.795889950
0.052499995	0.439560003	0.782079983
0.402780007	0.638189977	0.848239994
0.264279995	0.107280002	0.862559986
0.595300041	0.027200000	0.816639996
0.432169989	0.858100003	0.927670002
0.689499979	0.459599998	0.845399952
0.561689952	0.813269967	0.829959965
0.190050007	0.838500006	0.754939985
0.888769978	0.699320035	0.857420063
0.987080003	0.037069998	0.803919983
0.071990000	0.679189955	0.878979969
0.092940003	0.172460005	0.886800003
0.517289978	0.156259999	0.856360054
0.927389975	0.421739990	0.718840027
0.374399986	0.318060009	0.845510006
0.145480003	0.920859969	0.785019970
0.857849980	0.139069999	0.785830021
0.211360003	0.228000003	0.852120018
0.438559990	0.091460000	0.830089951
0.060289999	0.716310006	0.809570026
0.145610010	0.026100001	0.817930031
0.676429987	0.824320035	0.770550013
0.800130024	0.078440000	0.737230015
0.399359990	0.728810034	0.873250008
0.113420000	0.356869987	0.726739979
0.326510023	0.952000067	0.721000004

0.781359957	0.083729996	0.624209976
0.356769997	0.717249964	0.776809978
0.084799995	0.034669999	0.704220009
0.147339994	0.580259993	0.717050028
0.492629987	0.155870009	0.751659966
0.561140021	0.412790003	0.810589981
0.051220001	0.548060009	0.656389999
0.550499986	0.146180009	0.682730007
0.171210002	0.013770000	0.639120007
0.413049983	0.472229994	0.769249964
0.180999997	0.267289986	0.701489973
0.764209973	0.164739993	0.590990019
0.403510003	0.695169963	0.698880005
0.789190000	0.467619984	0.650129986
0.751779957	0.709339949	0.691389990
0.448669997	0.999210036	0.689219999
0.206230003	0.742609971	0.695110035
0.069279999	0.433209975	0.641569996
0.999329977	0.829880016	0.645400000
0.065909999	0.695519986	0.691720009
0.653389984	0.232899988	0.709689999
0.908500057	0.594839966	0.713039970
0.796459969	0.274349989	0.691639996
0.369780015	0.592990004	0.759890032
0.674460013	0.735109999	0.630729961
0.766390036	0.575339982	0.723710012
0.640290041	0.989300066	0.725179958
0.330669987	0.181169997	0.684639978
0.943249959	0.221860002	0.720079994
0.841929999	0.813359996	0.636369991
0.186590004	0.136750003	0.666760015
0.926889994	0.195120012	0.643470001
0.884729936	0.939230005	0.603079987
0.971000027	0.014939999	0.612400007
0.711940005	0.410039995	0.705919981
0.283719989	0.505490032	0.630460024
0.595589982	0.309630008	0.646679974
0.867160065	0.029180000	0.516240025
0.648209985	0.009920000	0.647259998
0.058290001	0.481630019	0.568920040
0.843049957	0.695569966	0.568840027
0.946739973	0.373199990	0.536040020
0.297650021	0.156519992	0.578079987
0.739660026	0.894569988	0.526770020

0.354339991	0.856989971	0.569299984
0.861779978	0.606750003	0.568820000
0.719739954	0.851340016	0.453700018
0.811749974	0.101549995	0.477169991
0.205779995	0.842630052	0.533209991
0.557959996	0.952350034	0.526920033
0.520309961	0.367179987	0.598370028
0.069059995	0.557299999	0.482270002
0.480660002	0.667729955	0.600780010
0.267930002	0.191240001	0.509619999
0.949819954	0.337289986	0.464130020
0.054140000	0.929679987	0.501879978
0.204589992	0.738489965	0.555850029
0.343600009	0.643819963	0.589960003
0.683610007	0.225649997	0.510610008
0.841750006	0.257350006	0.518879986
0.973869968	0.862280029	0.523950005
0.489149973	0.935530027	0.597729969
0.079149996	0.681389995	0.556079960
0.256949980	0.335670008	0.557789993
0.226140003	0.078890002	0.505509949
0.621999956	0.474410009	0.564650011
0.639890008	0.690450037	0.505000019
0.128920010	0.473249997	0.459070015
0.133509994	0.065070005	0.445790005
0.668160008	0.768670025	0.530859995
0.755859949	0.477580017	0.523180008
0.467800002	0.191560000	0.513689995
0.517350024	0.268860013	0.547779989
0.117930000	0.304100009	0.527309990
0.157519998	0.703530016	0.452099991
0.627439993	0.441970001	0.426979971
0.233300000	0.304839994	0.429500008
0.302189995	0.013290001	0.432299995
0.815159991	0.213230000	0.429430008
0.715219979	0.683879983	0.372440004
0.485370018	0.556249986	0.499199963
0.556100017	0.248459983	0.428039980
0.399419988	0.759389984	0.476499987
0.919409985	0.007170000	0.394250011
0.244489990	0.686850017	0.394190025
0.480089975	0.691850017	0.446640015
0.036440001	0.944390040	0.375400019
0.779250036	0.428400019	0.405019999

0.832480016	0.658649999	0.418489981
0.912169968	0.177810000	0.375250006
0.251120006	0.244369987	0.368569994
0.635249989	0.244439991	0.358999991
0.330870004	0.982510013	0.355069995
0.512939997	0.564379999	0.426189995
0.449460015	0.422800016	0.484019995
0.206830004	0.492859980	0.355380011
0.915470037	0.309430007	0.374210000
0.351609997	0.101539996	0.381819987
0.993239964	0.397170002	0.369830012
0.427659965	0.185530013	0.360759997
0.471899993	0.348139998	0.424519968
0.184880008	0.401750003	0.366479993
0.042920002	0.631439975	0.398259997
0.465509992	0.950989993	0.424790001
0.293889993	0.881659973	0.411779976
0.776429997	0.891569999	0.362290001
0.204900004	0.813940002	0.382800007
0.749689987	0.801800038	0.370730019
0.968509976	0.558759999	0.369760013
0.051639998	0.072420004	0.352030015
0.497270006	0.862590002	0.455110025
0.122950005	0.155980008	0.349339986
0.886349972	0.058940002	0.109480000
0.604449987	0.533230028	0.132070005
0.840610049	0.362740024	0.135299993
0.152899990	0.572309984	0.134459996
0.819100036	0.793439986	0.100520003
0.894160017	0.930090030	0.097180003
0.427219982	0.289520008	0.054809999
0.629719955	0.339949993	0.075839996
0.505630030	0.854239990	0.121579993
0.886229976	0.416579984	0.077030003
0.133149996	0.646200007	0.091480005
0.635460030	0.575449982	0.102559996
0.786869991	0.773210003	0.028880000
0.857969976	0.135550010	0.067610002
0.370539986	0.888519975	0.088100004
0.193130000	0.503180048	0.044810000
0.592239963	0.396510007	0.020310000
0.272729991	0.286309978	0.054900002
0.951219998	0.947789975	0.025470001
0.982339987	0.736419949	0.098650002

0.886840002	0.504459989	0.015300000
0.049959997	0.320030010	0.059490001
0.128949996	0.103809999	0.076319999
0.155339995	0.377160018	0.022849999
0.266600004	0.148320002	0.060159999
0.714779995	0.667339994	0.085810000
0.005570000	0.531130002	0.064359999
0.103589996	0.793329987	0.122950006
0.312110009	0.485779979	0.001100000
0.528959974	0.085239996	0.073760003
0.457360008	0.168390006	0.078230000
0.800079978	0.258579995	0.080339998
0.679979999	0.197900002	0.066919994
0.123449998	0.928229972	0.069440001
0.350650009	0.692250020	0.113720000
0.232779995	0.996359985	0.076070005
0.409740011	0.767080000	0.073379999
0.582610035	0.930119984	0.031430000
0.638890046	0.531460000	0.976159954
0.699450040	0.304619995	0.979419994
0.563159993	0.195759996	0.979399967
0.974439994	0.837350034	0.877070045
0.758809983	0.741590024	0.917409992
0.242280000	0.746539989	0.017980000
0.884769935	0.322409998	0.918249989
0.877300071	0.541960017	0.950920010
0.115709999	0.979749992	0.943840027
0.519720032	0.549709997	0.722889996
0.562980000	0.428950015	0.732249975
0.433659981	0.462209997	0.593460035
0.537590036	0.397689989	0.538269997
0.687520005	0.354399997	0.501270008
0.754390004	0.489950021	0.095070004
0.783269981	0.485119984	0.015750051
0.027230024	0.498680009	0.913920021
0.948990033	0.515690005	0.836429977
0.984449956	0.343169980	0.909759998
0.914780015	0.498680009	0.913920021
0.725419982	0.307839982	0.930900002
0.675429977	0.948790009	0.038320002
0.484350008	0.193430001	0.950049973
0.018470000	0.995710030	0.947780037
0.910009952	0.880020025	0.882200050
0.172549988	0.771910037	0.988220024

0.760870002	0.786719993	0.950250053
0.836539960	0.515690005	0.836429977
0.781390004	0.121200007	0.926550007
0.153129993	0.605929973	0.919790077
0.046590000	0.145979994	0.963240051
0.454270028	0.017630000	0.974069977
0.274610017	0.328379994	0.851609993
0.173200000	0.475900019	0.836759949
0.306389982	0.052280002	0.863990021
0.912200015	0.240160006	0.806589985
0.493829990	0.815779979	0.912249947
0.789679982	0.677290014	0.857699966
0.769009986	0.891460000	0.765290022
0.523899971	0.279500011	0.828890038
0.228299994	0.911380010	0.933170033
0.599459982	0.971170053	0.827519989
0.418209982	0.559389984	0.863189983
0.588610003	0.794410009	0.785039997
0.346509996	0.690940014	0.847119999
0.069390001	0.013050000	0.787389994
0.166950005	0.175170005	0.851329994
0.221769998	0.883930018	0.780080032
0.016800000	0.410140009	0.740600014
0.818960041	0.087370003	0.783780003
0.530080029	0.116379997	0.821889973
0.597069975	0.435759982	0.851729965
0.027909999	0.725039995	0.855159950
0.497380002	0.183989996	0.708230019
0.355250006	0.982939969	0.681570005
0.067179999	0.588879983	0.691820049
0.710349968	0.755530027	0.671930027
0.450529975	0.428950015	0.732249975
0.785480043	0.446189994	0.695819950
0.194119999	0.315949998	0.732670021
0.764859997	0.138820005	0.635739994
0.100799998	0.049290001	0.655560017
0.403749994	0.738500006	0.733780003
0.008020000	0.464069999	0.613349962
0.826520047	0.618369981	0.733200026
0.640250043	0.032019998	0.692229986
0.105910005	0.746730033	0.697249985
0.964470031	0.235580006	0.673799992
0.238390002	0.187309998	0.664619970
0.881789950	0.996569943	0.591939974

0.923299984	0.839550018	0.615180016
0.706740008	0.281870015	0.713249969
0.407270007	0.549709997	0.722889996
0.321210005	0.462209997	0.593460035
0.740479995	0.844479958	0.503180027
0.117339998	0.506820007	0.497900009
0.295850016	0.818180048	0.549510002
0.844880033	0.047570001	0.470069981
0.566399968	0.961220032	0.576219988
0.912949938	0.654319979	0.562419987
0.322530003	0.148249997	0.529349995
0.513230034	0.316179981	0.620029974
0.963780009	0.323979997	0.512740040
0.187699999	0.035829997	0.478280020
0.575070029	0.354399997	0.501270008
0.531029993	0.236119990	0.506309986
0.406319994	0.646629963	0.626600027
0.218490001	0.301380010	0.522429991
0.781620043	0.211600009	0.510710001
0.721639979	0.468579995	0.569350004
0.171709996	0.689529993	0.538169956
0.065470003	0.873260038	0.509049988
0.632599992	0.717109955	0.547830009
0.714730045	0.472030021	0.418149996
0.971739999	0.980920044	0.356649995
0.764089978	0.701740018	0.411889982
0.545369986	0.259059986	0.379410005
0.839589990	0.217040006	0.381859994
0.463660013	0.747929984	0.439109993
0.149129999	0.700540011	0.402790022
0.448530002	0.583090019	0.459369993
0.363700002	0.017909999	0.389899993
0.206369997	0.294609982	0.382719994
0.338630002	0.157519997	0.366979980
0.043280001	0.128670011	0.365829992
0.925469951	0.361950004	0.391840005
0.471560006	0.404450004	0.433039999
0.143079997	0.455930001	0.370530009
0.703949967	0.852680032	0.372630000
0.964710022	0.598150038	0.404799986
0.228530010	0.871399980	0.376480007
0.485870000	0.918079978	0.465560007
0.919949965	0.089919997	0.072449994
0.959439979	0.911769972	0.064120001

0.447489993	0.851340016	0.082000005
0.219269996	0.464569988	0.008840001
0.641939979	0.489950021	0.095069999
0.830739985	0.747009996	0.072080004
0.561889993	0.345019997	0.040329999
0.350399995	0.251500022	0.054890001
0.835630043	0.365200001	0.087269998
0.162870002	0.590280018	0.087679994
0.393689986	0.707959994	0.071749997
0.220230008	0.939510024	0.080480003
0.536639976	0.137600001	0.091830003
0.670960000	0.487430023	0.013750000
0.214630001	0.099760004	0.050749999
0.122199997	0.321909981	0.023730001
0.949580059	0.486020003	0.051289999
0.080859998	0.740039995	0.108770001
0.628000020	0.635180004	0.091439998
0.772759990	0.213990010	0.052550000

Final state of AIMD in C model

```
   1.00000000000000
     9.8709001540999992    0.0000000000000000    0.0000000000000000
     0.0000000000000000   17.0969009398999994    0.0000000000000000
     0.0000000000000000    0.0000000000000000   20.0000000000000000
   C    H    O
    64   228   114
Direct
  0.7333231276417069  0.1868088660235853  0.2236743587090828
  0.4851200542325171  0.1875027142968477  0.2204412323282969
  0.6125347660879016  0.9798963216619572  0.2151180408343931
  0.4890916715808480  0.8545543956569744  0.2250858693593130
  0.3604196092257178  0.7287061531988350  0.2300217707615632
  0.2348108316741325  0.1899052506330630  0.2274756153357300
  0.2338028187851258  0.6047812829274604  0.2351085701480053
  0.1125313228137668  0.4812749112309346  0.2327396429485739
  0.9833314296695828  0.3552222712700300  0.2357532714073524
  0.9867250570794452  0.1887465131050963  0.2300450015720223
  0.4843703828330903  0.1049437447090786  0.2160523229246934
  0.3634360037321876  0.9803122660944948  0.2182294678680199
  0.2379179359866760  0.8551527582928656  0.2248112896007123
  0.1109862673438175  0.7302560987658328  0.2302210529612222
  0.9857847701864880  0.6892397977480068  0.2298554828301707
  0.9839910763610236  0.6063187843922725  0.2321996431133778
  0.2340142844239897  0.1076199487194118  0.2254316525157973
```

0.8587039476386403	0.2299105418258835	0.2316080303748588
0.1113241566726058	0.0651622193727486	0.2230712974372489
0.1114476306881310	0.9819659647211525	0.2202565236356643
0.9867906655339486	0.8561629734890119	0.2159368357925570
0.8630414449165236	0.4805784762833810	0.2332430221094779
0.9836827336297164	0.1055210346160804	0.2241006187379860
0.8605329407176000	0.3145994405311713	0.2360459787212275
0.8622252365351534	0.5643504552766136	0.2329513124084062
0.7358529820282002	0.4371986318256511	0.2294316791976154
0.7341916586436249	0.3538895406098123	0.2306734168919455
0.9853966395356808	0.9396577181937318	0.2162878064184159
0.6111640277841860	0.3115460984584565	0.2262808583133473
0.8612435696912534	0.8145682447634411	0.2159369704811538
0.6094269411351806	0.2290078387319161	0.2213128977267358
0.7348561132401777	0.1025727741272923	0.2148497787007185
0.7403438899238773	0.6888788006950306	0.2337994528655705
0.6097559586052348	0.5618710307089733	0.2328485927736767
0.4849164056304868	0.4382650462548691	0.2304989540733671
0.3602669379633215	0.3144140020620567	0.2272200785760890
0.8615937136582839	0.0626502286199031	0.2166444837258759
0.7361718367906308	0.9370660014083995	0.2137593388498058
0.1107773122865533	0.2313347942391446	0.2318890439658942
0.6132776391520400	0.8122441276119433	0.2236989573868775
0.4905966527748307	0.6878852675625649	0.2337194884308250
0.8608592384517444	0.7317068232281604	0.2278846510808786
0.2343871271159444	0.3561276802166113	0.2252062764959845
0.3577236732165677	0.5632710561917696	0.2384476714547197
0.2352384568916531	0.4386795066442317	0.2300066594450827
0.7363823029508113	0.6045270960207283	0.2345651115641539
0.3587998527474404	0.4805368888820993	0.2348186170095976
0.1108783441742088	0.3159419971670722	0.2293987696928471
0.6094218888151637	0.0630059073159042	0.2128489940371937
0.4875692307084695	0.9377445383115055	0.2187975402124717
0.3629453201020247	0.8140836813660016	0.2252655833494971
0.4854967199883792	0.6028813910793660	0.2359694100156647
0.2365184247958398	0.6879561444207632	0.2353486179932207
0.1100239478917665	0.5646065265773758	0.2311712260462048
0.6102970031939213	0.4782328338832008	0.2263612750695984
0.9873295538871923	0.4395202308355953	0.2354575520927586
0.3616642506888003	0.0635438717844379	0.2170546735014549
0.6135965330982368	0.7294431296555102	0.2304459634400144
0.2370117634514901	0.9402914342268295	0.2230536770130712
0.1124277127002441	0.8149548253274043	0.2244626713592467
0.4846771491093753	0.3551044824510884	0.2288973952442697

0.3599813402708287	0.2302750072858143	0.2255122132158604
0.8610548359915001	0.9787904398886402	0.2134709040174824
0.7353247875521838	0.8550284780255204	0.2167836993445404
0.7515790956094383	0.0096869659086698	0.9454759210324563
0.1209605329324524	0.7351906630586695	0.9903109064715711
0.6616396918214078	0.7474775019331692	0.9260886382000566
0.9108671789802893	0.4207838131185027	0.9734956710030823
0.8387922935755284	0.8293991284927994	0.8695688197784693
0.8698920370923330	0.8524170626317091	0.0550698372252253
0.5725583119601600	0.1230409538863596	0.9716239853877091
0.5416361319834910	0.3049965625874672	0.9884352039881841
0.2891932983043973	0.1699567727116845	0.8798682270291545
0.1745060116306346	0.1751421489957679	0.8219427786681321
0.8334371965433777	0.0922604631560685	0.8343981195097100
0.3743201048140566	0.9470632651323712	0.8535658276238869
0.8175305354417670	0.2176829028271323	0.9965532986365641
0.3581237194465935	0.3002149251693452	0.8429388722201107
0.2004592545736369	0.3316808105457179	0.9337541655971382
0.8239512010484011	0.1347157589246433	0.9041628355754319
0.3332426798599951	0.9023522824227627	0.9168811435078655
0.0295494730481842	0.3714309665867888	0.9370116480426469
0.8047011887675790	0.4066571612812264	0.7943807240607511
0.1627983038710347	0.3956569190733912	0.7011043014691944
0.2129988282516151	0.4244454216790444	0.6265298977179679
0.1285651825587543	0.0868164281027342	0.8988654940208520
0.7924337831315010	0.7521733801548082	0.7342924676917687
0.5367988898632187	0.2100713052560944	0.8091467834878527
0.9533256291673055	0.0247895958722951	0.7785540275479284
0.2748922058776100	0.7941122166305425	0.9612983538363259
0.4314492696203571	0.5959458004920054	0.9434163502813659
0.6779088564845405	0.8921843752418769	0.8424144235216194
0.8104744675848596	0.5512265273964868	0.8761185521957405
0.3354873554100204	0.3837754163389115	0.9382753593992760
0.9192711626526864	0.0726853597021867	0.7108886342640660
0.4082400040665135	0.7775766125287144	0.9204519694944020
0.8635226471732617	0.7359728681341849	0.8059289864700512
0.6899037286107604	0.1824246551573222	0.8078854302424393
0.9945326045066880	0.2366573995837418	0.7823325212258012
0.4401780558607366	0.5299723918776073	0.8007241181308504
0.9775976420530478	0.0522418036170110	0.8934642092238132
0.7058695459127002	0.8932165502965964	0.9177431322971351
0.5482685909438026	0.6633341282067693	0.9607191203599046
0.8261135717553582	0.2794505102678056	0.8212409712369405
0.4336962088414816	0.7489719665944133	0.7955084620260137

0.2045291495004993	0.8046809614614046	0.7507916129767743
0.7590331658231372	0.6193022086575515	0.9149050764049104
0.9841919444329997	0.8933988047658841	0.8259139433342700
0.2531869861792042	0.5674139503527574	0.8731774998353552
0.2826655875877506	0.1008835337920761	0.7785802621613029
0.3897553465680138	0.9967549990050038	0.7521675821865531
0.0912631355261145	0.2125223233388238	0.7230736595669066
0.3548309281655012	0.2655362349448764	0.7747665749396052
0.2674125238843080	0.8601403329844796	0.8088256334020569
0.7773441947192701	0.9971865069298478	0.7563620604282082
0.3193760790613482	0.1206189289622250	0.7003294435094660
0.4064997294382957	0.9027948616561716	0.7679473556052875
0.1530995986985150	0.5531884107236761	0.9387286792847032
0.1053341332779773	0.9533639427279579	0.8392740805003300
0.5910654890808658	0.7211622982214945	0.8028902418515941
0.6211746150994393	0.9580958479333023	0.7657131765296082
0.4495554483292989	0.6222439918965768	0.8140247147919163
0.3907127881621465	0.2007719693079309	0.6204370754942934
0.6103764157882108	0.7848180102160230	0.6615226866657585
0.8486872673812684	0.0790661243723862	0.5703699401898743
0.4236232325076828	0.6131818336589695	0.6797444084647117
0.2361096184802878	0.9207964598675960	0.6454596762030115
0.0592973096052365	0.5493211574818743	0.8385075677285250
0.5196668441597181	0.3616015427416007	0.6155799880598539
0.7984416175801538	0.2526502290886034	0.7486942204188473
0.0630245244764149	0.4807588166535190	0.7829933914069527
0.4647998394303840	0.3395408457354988	0.6983904048665069
0.3300698263626139	0.8598763486698170	0.6753646678410293
0.4805613330777305	0.3826506686863574	0.7983037825833538
0.5012060268241326	0.1773675689942903	0.6733539665646667
0.7517494385468597	0.1086993632709320	0.6367143964726631
0.3550833009917544	0.6256680609407225	0.6114538622022180
0.5536741387472468	0.4847370809408281	0.6950015825519085
0.8198950763222758	0.8546869275026663	0.6453458510811080
0.4786909014005032	0.7289325491932276	0.6727239478845766
0.1812588257445207	0.6312992017721468	0.6883168810833173
0.9939484326562552	0.4129408863652302	0.6333900097511228
0.0775465386222369	0.7166166539768620	0.7151897245541048
0.0632016057472624	0.6008990548760823	0.7418057232170239
0.6863896114262373	0.3181037830392572	0.6772194992149163
0.8679610703778025	0.5488838703009092	0.7697303907266720
0.8496871077226694	0.3169939544952516	0.6716237631250118
0.1062989475248484	0.3349916761800425	0.7834514112692837
0.7665151054133630	0.8758725622447411	0.7160594191691275

0.7793137521051775	0.6299364570068597	0.7696227735917295
0.6065728681962991	0.1214882550407222	0.7409313962964758
0.5124137555521906	0.0102164545934287	0.5602920845567890
0.9995061843979630	0.1404380494783070	0.6344752184217862
0.9970518244429680	0.7985273403776642	0.7183830958912686
0.4497346246460385	0.9693676857991160	0.6198654511762005
0.0909134913200524	0.2100223483684505	0.6048144796478349
0.0639143980875620	0.9344519621561931	0.7211140473687735
0.1504129809545560	0.0035019841200429	0.6953096635347190
0.6549129885774619	0.5532952909966685	0.7208385085537605
0.2487708422362477	0.4433055866527437	0.5114128295608863
0.4269430331631416	0.2707277869028524	0.5285664582317458
0.9345930017867700	0.9704215177266085	0.5129555503256851
0.5825782079372097	0.0669622537612360	0.6697113386147906
0.8912616677718368	0.4760726132983661	0.6195724191672511
0.9281850368568595	0.6130016626894836	0.6419686267732042
0.8175849958974959	0.4147902472931322	0.5500787194140505
0.3285802232206245	0.0589260260718989	0.5742543243337104
0.5410443087669345	0.8909172168581571	0.5027244298934960
0.0938286837193082	0.7929897166607858	0.6105545091177398
0.7857907953413903	0.5853492502272465	0.6002912755768589
0.6947578251730694	0.8796268910341365	0.5216320402091582
0.7748792599758624	0.9953259798063551	0.5099021861817111
0.0695638871774427	0.8391764727448388	0.5419986001591673
0.4587243504576162	0.7826810247213999	0.5180202007551863
0.2911436903523327	0.2273351730885778	0.5339730071534990
0.1386201696133233	0.4289539710917962	0.4156976658092463
0.3430908942442772	0.6336192593338363	0.4903241268546668
0.1789624085746171	0.0921481701820026	0.5835209202441295
0.7668314978915587	0.4370546668544554	0.4677456000395563
0.8201221880796923	0.7963476407846041	0.4768439058636831
0.0479342124183640	0.7073684571383053	0.5206064465744241
0.1995955994401699	0.6311855199817451	0.5292572026959443
0.7630954312900331	0.1912050029490779	0.5631423968653301
0.6962305505568850	0.2645196382829887	0.5794547402841094
0.9019002007786675	0.7967150785689047	0.5516925375579188
0.3807381848520761	0.8460472190785240	0.5603291506130913
0.9686890588757099	0.6263797584219277	0.5378612033989241
0.1186034023963657	0.3226656032388391	0.5564384741708039
0.1351955789066273	0.9704113415111691	0.5205248939637200
0.6003415824738599	0.5594797881003416	0.6073039895359987
0.5830382443235758	0.6694501417248093	0.5305999621853549
0.2538801739657769	0.3587494493385516	0.4141259602182874
0.0771626257454827	0.9443320997240542	0.4514091581763310

0.6831892514550726	0.7497406083493744	0.5301870731488674
0.6486851255056910	0.5257178033850781	0.5379050065056267
0.4882008035217923	0.0444264959688701	0.4325811689419185
0.5057963340533463	0.1187060037485857	0.4829328863478753
0.0575757025190667	0.2647032015405871	0.4993270609150110
0.1011984661807567	0.6329719803953618	0.4305659948376838
0.7076664602799345	0.3688952704757777	0.3698776477612981
0.2546655610781980	0.0877430376941929	0.4068000885374508
0.3429340362762602	0.8613711155873424	0.4460085223562616
0.9203701529680935	0.0831666051593474	0.4287914679893763
0.8899453008198349	0.6313943338458033	0.4513691421043300
0.5276509848153946	0.4641035356413210	0.3782239526496277
0.6149884685721615	0.1351944137274909	0.3996339058027719
0.4695487261743701	0.6647423342924063	0.4061830032586410
0.6496630799586715	0.9138495635245780	0.4153206608584852
0.2287984194991170	0.6284211250896731	0.3839933174654757
0.4260517963818189	0.5763594468873086	0.4084470274243284
0.6193281958272395	0.9510838388220173	0.3473535829661885
0.8479984879040120	0.3536046678826413	0.4048269846924304
0.7480509256311138	0.6235265004190134	0.4713988555446463
0.0447472397156887	0.1267879141046757	0.4022557341083547
0.2082519971761985	0.1132290915982253	0.4759334598866131
0.7486976111919470	0.1443930075446699	0.3618448430608951
0.2501687877061053	0.8302853780909879	0.3924404108779965
0.3867524114026918	0.4572864850187109	0.4066110537539001
0.5245139745360813	0.3267714023006782	0.4199847419409077
0.3587435223074346	0.2708791628706723	0.3624781995798799
0.8474585249652132	0.2680852996182715	0.4862863463994480
0.3438630536181034	0.9540043501477606	0.3739215288734883
0.9166667881302653	0.2219308398877130	0.4286144173018890
0.3911150085532205	0.0178122832654665	0.3266706211085715
0.5954796899128324	0.2535498954800066	0.3790760364073647
0.2372891463794612	0.2241564360673618	0.3919134029079258
0.0483836885199363	0.5366915034249565	0.3820966474380222
0.4846591192467747	0.7885138486752901	0.3739191344852129
0.0198800997085856	0.8081739735781376	0.3861231198117799
0.7670293390147704	0.8262479117707241	0.3815070051782873
0.0991166649129051	0.7278559841432646	0.3829631423837090
0.8230255848186174	0.7384263435149224	0.3867055674660440
0.9194268231949608	0.4786658778460643	0.3784239470600702
0.9122581125450068	0.9578278488337201	0.3714380911513760
0.5815277947602475	0.7427514504328686	0.4261319813334969
0.0441120768156272	0.0065195162417716	0.3523914825531615
0.1559076906814286	0.2085757328481717	0.0685395937493245

0.6249841750491407	0.5325784456460643	0.1030079516846731
0.8809805436560764	0.2141310084119362	0.0962791547439547
0.7813536212401617	0.4128004516452967	0.0566898175660010
0.7875232814002812	0.7440673959900050	0.1152150488644279
0.2201562266759531	0.9389730266480951	0.0582615850835229
0.6780036789872633	0.1441108426116214	0.0793437752029904
0.3795863352298715	0.2684051625488943	0.0548276998679519
0.3844852622827852	0.6570596804111913	0.0428916809466813
0.9075226871068954	0.1295964631087838	0.0748107028753562
0.9004319698262260	0.4704199935050237	0.0714626965661274
0.8319119922922873	0.5895846463622380	0.0618491900293297
0.6518413274138897	0.7757121293858521	0.0837476872572056
0.2248834433759189	0.1380998061130544	0.0340385536415026
0.4067935966643035	0.6647692047477193	0.1217191490297168
0.2526587227150456	0.4356583590500548	0.0673736283997317
0.5197425636308219	0.2247516359483315	0.0706446660752549
0.5587788305903911	0.0896947088138541	0.0866319305149725
0.2319984670339288	0.8608600918652098	0.0958701052873732
0.1741568539472632	0.7006173171516581	0.0825524855999997
0.5622374659744636	0.4381305513102462	0.0197956151697479
0.9844969278307625	0.3395357922468494	0.0908080967872123
0.3813411817766653	0.0726041853425801	0.9632055140831611
0.1296230984849026	0.3144592390802578	0.0949359151615304
0.2559368384978910	0.0176875849374548	0.9569389738188931
0.8895698447692812	0.5738684726478779	0.9900263988387324
0.5639733712155046	0.3650582981541214	0.0738337483586783
0.0204892381430727	0.6792511300951016	0.0568565953979151
0.3485563023625832	0.3795016978132941	0.1009793647295774
0.5923152452897914	0.9799634365950526	0.0421015472681056
0.4318040252769752	0.9931592865533337	0.0544366489245458
0.0423029999253044	0.0615310033516349	0.0210733843090486
0.9889863088012139	0.0194988799467422	0.0850348509823806
0.5173070607752789	0.8597587082059378	0.0811784359076991
0.0891687002171023	0.5520551159969896	0.0526439128271262
0.4780015972550753	0.8049492399427438	0.0240376421589594
0.2405401354100706	0.5725497933208755	0.0582604080576040
0.8164074508441088	0.9496429672712098	0.0069984770962044
0.4858344463449110	0.4539615096890985	0.9060455049535981
0.5555295119280546	0.2357275661386614	0.9392049208303103
0.5582272287081034	0.1294870039895206	0.8895530337765786
0.9960047010269044	0.7979213232231894	0.8873875361413934
0.7364068446174291	0.7180717892959999	0.9968135362265730
0.1688051642708237	0.7107297886052882	0.9167371023106959
0.7369331281435967	0.2692226238692452	0.9437688133421464

0.9957863463625958	0.2512225356388291	0.9563654685644523
0.0054693705335717	0.9024605316108644	0.0583531048058345
0.1628524061388015	0.3911117926610501	0.8397069650216227
0.6149373740915005	0.4042410789273608	0.8305764627865254
0.2939647110037159	0.5174995932683607	0.5553715052889504
0.4469982285732610	0.4144099927601398	0.5537078019502826
0.6033379936129056	0.4008710981315661	0.5306657056980177
0.6224620453531400	0.6049882746636548	0.0568114499579650
0.5913318951680607	0.5166800818876552	0.9308196356812681
0.1601706580980322	0.2317173369895668	0.9752248436585085
0.8388147706652768	0.4052679655307980	0.8756351094218462
0.8169709360312611	0.2369914936773904	0.9507400192002192
0.0943043604970374	0.2603987378239589	0.9483796231064862
0.5623550016182046	0.2925560557226819	0.9394377836900554
0.7265309147953647	0.9723547269931652	0.9818672112900481
0.5179737812415509	0.1389286794583612	0.9337221922412564
0.9053378272782537	0.9069116073453952	0.0546215005058752
0.9338516259184999	0.8132754602517042	0.8515553092126115
0.1291966369570919	0.7552671360875306	0.9438246118182609
0.6983087134961752	0.7043164864442141	0.9496252645989135
0.7745717914756093	0.3847924178243834	0.8359333928822229
0.7975086027571163	0.0841735222331425	0.8802559520716629
0.9590955466522392	0.4131118848384601	0.9313250002459058
0.2329862061895859	0.1418075067427995	0.8487811549860517
0.2930676237749748	0.9292596148663226	0.8780240895959819
0.4221150273468335	0.2900548498239513	0.8024759208711593
0.1549983780627126	0.3859143071615075	0.6536647961409934
0.0635913365414723	0.0471809732330320	0.9157409887266936
0.9072469695515348	0.0717621199199362	0.7585490483608349
0.4547382147912278	0.6416497263370061	0.9694048637826279
0.7904239514576553	0.5665486164546238	0.9224292782570831
0.7918912548304486	0.7168817279895278	0.7763479169894943
0.5975270189275053	0.1633076470465671	0.8067789895062921
0.3677949490171538	0.8119926099167288	0.9530929537182370
0.6846009742137185	0.8572551141366681	0.8829582905460482
0.2760454789777563	0.3584750354061493	0.9046756748393704
0.5027967072640833	0.7236663171421149	0.8249081533680940
0.3913815267557125	0.5787781885673702	0.8085709268355452
0.0397861576348878	0.9363987135373902	0.8055160544953432
0.3090118517436927	0.0739253257965123	0.7355318680771695
0.2899830907950920	0.8237051886894958	0.7724519158446479
0.0924123143879451	0.2447591143224533	0.7645443951535655
0.7226510112073679	0.9473097564107084	0.7635735422440733
0.4563828394599436	0.9556847942631179	0.7699444842962058

0.8470589327534498	0.2307394825843588	0.7950889994323067
0.1659296964043585	0.5780954407820046	0.8943741031470601
0.5420644665247620	0.3613750206417818	0.6730619810374044
0.5204339873232849	0.7804694939348208	0.6835425019965656
0.0216046698174795	0.5310401188655282	0.7929667230321841
0.7862737947003557	0.8286310109327348	0.6854676404898036
0.5215039343762188	0.4271856788526218	0.8212846915843074
0.5890064169127077	0.5350453180804259	0.6831092637920846
0.4017683960981671	0.1905895689419885	0.6693534178830989
0.8288734197892972	0.1188865959537083	0.6065025284239485
0.3233651320673575	0.8920264339546787	0.6355267244608802
0.3487276088606265	0.6426582904856761	0.6612714560569422
0.8942538492623807	0.4209484394888059	0.6206076021884593
0.7713102492049337	0.5721708476850459	0.7684341890334175
0.6283639574985922	0.1116029054670914	0.6892041585503024
0.0833693145782042	0.6309359689351689	0.6994213574271103
0.0944521831735583	0.1629425387615848	0.6359147370621840
0.4891770126604507	0.0199654741492026	0.6064287161428152
0.0949309526323706	0.9602188037211318	0.6783029257018421
0.0857024497765551	0.7781490006698513	0.7104411288548260
0.7715249221352315	0.2838033401430502	0.6830591571440011
0.1283140900876193	0.3922831774829579	0.7919459233831672
0.2786932807036149	0.4603527244331627	0.5582565922162036
0.6322047057365755	0.9132106569501295	0.4991607330885197
0.2289015776350861	0.4120278930567928	0.4351555798874612
0.0518607643352298	0.7889498846608672	0.5689763138237743
0.8687081531062693	0.0149757019331934	0.5135261483890124
0.4124467326575973	0.8362839960806858	0.5155670788896981
0.8852658018802508	0.5829889806895074	0.6030597397094900
0.2337864748501557	0.0546766817201901	0.5543479454338843
0.3881552561720105	0.2180126010771339	0.5313763666840210
0.7436913259543176	0.4259955524399070	0.5156370343479025
0.0845385435443376	0.9274814598256338	0.5003058470503169
0.5149953337700707	0.3740413086678715	0.5442567778781783
0.5528040321554786	0.0764031730570788	0.4618893105534737
0.3000678996567840	0.6234497067648919	0.5378975766751848
0.1271026935079991	0.2700042584124414	0.5344947261262131
0.7103157602876811	0.2359247036605860	0.5396431755775367
0.6286081463967289	0.5766349237544854	0.5627918117445342
0.0314840036718879	0.6549837705098154	0.5047132117508488
0.8081856507942976	0.8037727521494221	0.5260540586366216
0.5952407521120364	0.7257552239650475	0.5179847717762622
0.7881919387658904	0.3972377991733725	0.3863813468988124
0.6979346056315687	0.9232466555514477	0.3713567218999765

0.8040598074553487	0.6187392372495321	0.4330577829326887
0.6622036355635573	0.1719118227512047	0.3696568521066749
0.9427957653425963	0.1190071689261857	0.3928207755594497
0.3837912007245705	0.6319998182145298	0.4128347861843939
0.1252955787284756	0.6265958289482246	0.3837523224457578
0.4622419245740595	0.4913252849456310	0.4052965614019582
0.3351832388334900	0.8596435107275923	0.3954601394895977
0.2039700084602877	0.1320376944232150	0.4274386197828173
0.3649990452572498	0.0120411132815732	0.3724335913039180
0.0119648965062325	0.9628657272979998	0.3771377543552822
0.9098348909279685	0.2718635701595975	0.4509639106344029
0.5563554128048412	0.3082229299188681	0.3757008802818281
0.2631696294278604	0.2748491592283377	0.3650367906283362
0.8451977323927047	0.7949425687678054	0.3954464270575962
0.0141910023392323	0.4831200875019913	0.3946074430324873
0.1124791152729571	0.7865738397395335	0.3912652491740850
0.5530760596492389	0.7472692103108970	0.3793880467693565
0.2353977442876726	0.1924210163491912	0.0439446969551098
0.1868181253415041	0.8860159362734943	0.0597719343720715
0.3333671880746937	0.6607748567826475	0.0861486526685926
0.2681504154732833	0.3814161116606807	0.0743420698576500
0.6682375662257768	0.5847597578159885	0.0990225334712152
0.7521051138734197	0.7626155252509451	0.0719747768289233
0.4778401801934744	0.2765790696935537	0.0625704614565514
0.5825497743190787	0.1390731167262564	0.0726673088861587
0.8343467310575365	0.1712451669296711	0.0724183788886989
0.8767178871470526	0.4153979447613661	0.0603548666665804
0.1830780504217364	0.5297555947963221	0.0425907130762991
0.5003937503452714	0.8042067289626516	0.0758466948249340
0.5198826038017658	0.9713076472135166	0.0759431708174551
0.5096223261294975	0.4857224299246771	0.9471163408658110
0.3107615716623074	0.0456195477687073	0.9875641600746055
0.0388389044693401	0.2913150258211540	0.0994094241981641
0.6051943794643948	0.4118500066796586	0.0581691266961644
0.0886944599684181	0.7195145805781563	0.0589208629328207
0.9178783590579752	0.5856173955225853	0.0390592837316138
0.0379387140273914	0.0649885115132564	0.0694398983946135

Initial state of AIMD in PCC model

1.0

9.8709001541	0.0000000000	0.0000000000
0.0000000000	20.0000000000	0.0000000000
0.0000000000	0.0000000000	20.0000000000

C H O

96 240 120
Direct

0.498490006	0.497460008	0.543990016
0.998679996	0.500209987	0.533550024
0.247869998	0.498140007	0.540409982
0.749140024	0.499159992	0.538429976
0.873650014	0.534240007	0.534460008
0.123960003	0.533729970	0.535579979
0.373160005	0.532729983	0.541400015
0.623399973	0.533159971	0.541689992
0.248290002	0.428999990	0.525600016
0.749080002	0.431070000	0.520460010
0.498809993	0.427639991	0.529829979
0.999100029	0.433099985	0.514259994
0.123879999	0.601019979	0.515510023
0.873790026	0.601710021	0.515699983
0.623160005	0.600619972	0.519299984
0.373299986	0.599810004	0.517859995
0.373530000	0.395880014	0.514900029
0.624170005	0.396750003	0.512369990
0.124310002	0.400629997	0.503750026
0.873650014	0.402819991	0.498540014
0.999260008	0.631470025	0.498719990
0.498340011	0.629320025	0.499529988
0.747309983	0.630330026	0.498879999
0.249310002	0.629320025	0.497509986
0.624230027	0.337799996	0.472600013
0.125420004	0.342869997	0.463880002
0.874189973	0.343809992	0.460700005
0.374559999	0.338180006	0.473529994
0.998950005	0.684669971	0.453090012
0.498549998	0.678749979	0.449169993
0.747399986	0.681829989	0.451270014
0.249219999	0.680239975	0.449239999
0.748139977	0.313930005	0.444489986
0.251410007	0.314929992	0.444970012
0.499029994	0.310600013	0.451189995
0.000580000	0.316859990	0.439779997
0.622850001	0.702719986	0.423970014
0.124329999	0.705910027	0.425969988
0.374150008	0.699980021	0.421539992
0.873030007	0.705720007	0.426470011
0.499289989	0.263179988	0.397850007
0.000630000	0.263749987	0.393759996

0.748870015	0.262499988	0.396270007
0.251500010	0.267289996	0.392729998
0.124289997	0.749000013	0.370719999
0.623560011	0.746829987	0.369439989
0.374080002	0.744440019	0.367430001
0.873579979	0.749740005	0.371749997
0.376340002	0.244760007	0.366250008
0.623179972	0.240630001	0.369949996
0.874339998	0.239680007	0.369740009
0.126719996	0.243670002	0.366479993
0.499100000	0.766839981	0.341360003
0.998979986	0.771139979	0.344779998
0.249249995	0.768130004	0.342189997
0.748430014	0.769800007	0.343840003
0.623849988	0.201969996	0.310470015
0.874499977	0.196459994	0.314319998
0.376390010	0.205359995	0.307200015
0.126149997	0.201480001	0.310310006
0.749149978	0.178540006	0.284590006
0.250770003	0.183310002	0.280019999
0.500569999	0.184520006	0.278470010
0.000420000	0.177330002	0.286000013
0.498930007	0.815339983	0.290639997
0.999170005	0.815760016	0.290679991
0.748950005	0.815710008	0.291070014
0.249160007	0.815410018	0.290360004
0.374199986	0.841009974	0.267239988
0.623769999	0.841589987	0.268279999
0.874140024	0.841289997	0.268009990
0.124169998	0.840600014	0.266739994
0.250000000	0.132259995	0.231549993
0.749329984	0.130720004	0.232979998
0.000260000	0.128580004	0.235560000
0.500069976	0.134859994	0.227290004
0.624549985	0.103579998	0.210280001
0.374900013	0.103660002	0.211109996
0.125279993	0.100660004	0.215120003
0.874670029	0.100149997	0.216649994
0.374320000	0.899269998	0.227469996
0.623749971	0.900219977	0.229519993
0.124360003	0.898649991	0.227019995
0.874379992	0.899839997	0.229440004
0.748989999	0.931490004	0.215550005
0.249290004	0.930339992	0.212449998

0.499170005	0.931410015	0.213560000
0.999509990	0.930249989	0.213080004
0.624390006	0.034740001	0.193299994
0.374370009	0.034610000	0.195069999
0.124949999	0.033240002	0.194749996
0.874830008	0.033020001	0.196380004
0.249829993	0.998820007	0.195869997
0.748899996	0.999180019	0.196659997
0.499449998	0.999719977	0.195170000
0.999960005	0.998350024	0.195859998
0.826259971	0.605180025	0.999289989
0.369349986	0.361299992	0.019780001
0.955439985	0.650439978	0.996349990
0.230660006	0.350510001	0.983709991
0.497090012	0.125980005	0.939159989
0.082359999	0.892769992	0.911130011
0.615769982	0.949949980	0.928349972
0.782130003	0.711790025	0.967509985
0.230059996	0.202110007	0.983229995
0.465449989	0.255199999	0.912999988
0.716639996	0.314770013	0.943849981
0.452419996	0.817430019	0.967620015
0.134169996	0.728399992	0.989030004
0.231800005	0.757200003	0.934340000
0.450139999	0.742179990	0.946510017
0.083470002	0.229800001	0.970910013
0.608020008	0.398750007	0.878400028
0.937879980	0.457729995	0.900520027
0.215110004	0.595499992	0.946269989
0.422710001	0.585959971	0.963220000
0.901889980	0.995360017	0.906539977
0.209230006	0.998589993	0.913580000
0.700349987	0.915239990	0.875469983
0.549109995	0.139349997	0.867919981
0.095820002	0.811900020	0.909439981
0.476159990	0.931349993	0.827250004
0.385870010	0.929929972	0.756659985
0.192440003	0.078019999	0.851849973
0.769930005	0.807330012	0.911099970
0.895070016	0.857999980	0.902670026
0.056680001	0.171509996	0.881850004
0.304280013	0.131040007	0.874830008
0.966440022	0.109410003	0.883639991
0.900229990	0.645609975	0.886770010

0.265020013	0.264609993	0.892700016
0.519720018	0.450260013	0.847069979
0.125410005	0.605080009	0.831439972
0.835030019	0.479609996	0.843370020
0.269230008	0.958509982	0.847790003
0.031569999	0.999459982	0.861109972
0.773069978	0.583500028	0.841180027
0.669809997	0.539420009	0.878340006
0.222670004	0.427190006	0.803499997
0.066299997	0.414669991	0.818369985
0.184169993	0.532239974	0.834819973
0.682139993	0.027729999	0.861999989
0.784630001	0.082860000	0.832880020
0.474790007	0.332230002	0.892870009
0.490509987	0.781979978	0.848819971
0.267109990	0.851419985	0.806620002
0.709840000	0.252319992	0.905969977
0.839280009	0.684830010	0.820590019
0.207310006	0.316320002	0.841239989
0.461840004	0.679629982	0.815370023
0.507560015	0.334160000	0.787599981
0.160850003	0.752460003	0.796109974
0.314720005	0.652559996	0.814570010
0.386009991	0.283320010	0.781769991
0.037939999	0.707220018	0.819999993
0.798479974	0.311820000	0.828390002
0.890049994	0.370599985	0.806159973
0.320410013	0.961139977	0.647029996
0.796130002	0.864130020	0.804180026
0.993730009	0.062040001	0.749159992
0.286969990	0.042769998	0.742829978
0.936739981	0.254850000	0.773679972
0.136600003	0.870119989	0.761629999
0.468160003	0.828970015	0.790899992
0.606469989	0.246509999	0.726209998
0.742020011	0.787660003	0.766409993
0.211009994	0.203869998	0.778859973
0.747009993	0.206860006	0.721629977
0.832679987	0.748589993	0.712069988
0.098880000	0.200080007	0.720220029
0.917400002	0.253399998	0.700730026
0.885860026	0.573379993	0.761789978
0.162149996	0.385030001	0.718760014
0.454800010	0.472680002	0.735289991

0.633480012	0.039140001	0.770359993
0.724839985	0.920189977	0.763989985
0.291280001	0.890030026	0.631879985
0.238959998	0.120820001	0.722599983
0.930800021	0.129580006	0.725539982
0.941730022	0.917360008	0.712800026
0.554180026	0.142350003	0.672100008
0.050409999	0.956889987	0.667890012
0.622850001	0.955540001	0.682569981
0.412869990	0.101599999	0.670620024
0.514259994	0.900390029	0.662819982
0.476110011	0.776870012	0.692139983
0.743040025	0.266669989	0.628250003
0.463990003	0.321570009	0.673799992
0.131150007	0.718010008	0.721310019
0.886089981	0.510209978	0.712859988
0.183239996	0.346760005	0.649999976
0.364820004	0.532040000	0.740740001
0.555339992	0.039299998	0.702910006
0.741090000	0.044259999	0.579479992
0.245289996	0.996500015	0.533999979
0.247840002	0.058690000	0.578040004
0.854830027	0.065569997	0.628939986
0.877770007	0.134680003	0.547670007
0.749130011	0.893970013	0.624610007
0.230649993	0.183880001	0.581629992
0.154410005	0.672200024	0.665350020
0.538519979	0.279639989	0.616280019
0.864229977	0.292050004	0.578999996
0.463939995	0.704559982	0.667850018
0.646619976	0.673810005	0.736620009
0.592540026	0.605799973	0.712469995
0.652899981	0.411199987	0.666350007
0.176970005	0.534370005	0.660790026
0.674470007	0.460579991	0.727370024
0.863349974	0.378719985	0.669679999
0.995299995	0.416640013	0.660920024
0.046519998	0.574500024	0.687309980
0.950760007	0.904399991	0.555880010
0.593980014	0.941009998	0.575219989
0.424340010	0.101709999	0.535679996
0.825089991	0.826569974	0.609130025
0.189339995	0.133149996	0.516669989
0.002040000	0.166979998	0.588159978

0.983969986	0.726379991	0.637969971
0.860920012	0.689670026	0.609340012
0.330379993	0.278710008	0.611739993
0.250919998	0.242780000	0.668200016
0.123760000	0.925989985	0.466380000
0.889370024	0.998290002	0.485870004
0.516889989	0.042199999	0.519159973
0.083779998	0.864839971	0.545350015
0.681959987	0.962800026	0.512929976
0.296649992	0.818970025	0.557869971
0.450729996	0.772199988	0.580770016
0.203529999	0.801039994	0.618560016
0.693300009	0.200000003	0.552630007
0.748950005	0.071640000	0.464560002
0.616940022	0.111720003	0.473580003
0.526239991	0.795469999	0.514069974
0.565029979	0.165570006	0.577489972
0.822170019	0.945980012	0.435699999
0.263229996	0.932690024	0.424860001
0.757759988	0.861639977	0.505699992
0.103220001	0.991590023	0.371499985
0.069899999	0.066480003	0.354099989
0.861630023	0.831839979	0.462449998
0.480390012	0.925750017	0.457650006
0.425509989	0.862049997	0.419059992
0.211500004	0.039910000	0.430979997
0.321980000	0.095859997	0.438859999
0.575510025	0.029209999	0.381220013
0.668780029	0.083549999	0.353300005
0.523639977	0.397029996	0.338079989
0.501689970	0.501470029	0.420709997
0.864979982	0.511720002	0.289790004
0.402869999	0.520929992	0.364670008
0.286810011	0.371520013	0.275970012
0.911220014	0.668349981	0.296519995
0.237020001	0.606599987	0.243009999
0.483060002	0.416480005	0.265639991
0.788820028	0.536130011	0.223120004
0.530399978	0.527970016	0.300359994
0.719460011	0.622269988	0.174410000
0.343780011	0.543990016	0.257939994
0.004790000	0.499570012	0.198500007
0.173810005	0.531130016	0.229110003
0.995280027	0.309340000	0.205080003

0.226530001	0.766049981	0.109399997
0.590170026	0.776130021	0.158539996
0.664059997	0.236090004	0.154679999
0.140210003	0.336739987	0.270080000
0.877860010	0.665870011	0.220500007
0.477019995	0.665870011	0.125880003
0.348300010	0.630039990	0.135759994
0.211720005	0.714630008	0.193959996
0.321119994	0.449200004	0.137769997
0.650449991	0.366389990	0.213809997
0.083520003	0.672370017	0.228230000
0.749040008	0.428479999	0.217230007
0.295659989	0.394180000	0.188569993
0.752550006	0.837849975	0.089280002
0.135629997	0.135989994	0.082979999
0.958459973	0.105339997	0.009629965
0.233630002	0.952889979	0.037140001
0.949109972	0.368090004	0.246409997
0.806760013	0.212219998	0.131369993
0.535759985	0.825659990	0.074620001
0.351080000	0.794950008	0.148120001
0.742309988	0.734499991	0.134470001
0.326240003	0.274230003	0.174449995
0.861810029	0.689840019	0.103390001
0.121110000	0.499020010	0.137109995
0.619310021	0.561909974	0.160500005
0.529999971	0.558160007	0.229230002
0.660080016	0.007930000	0.042010002
0.239449993	0.193709999	0.104369998
0.857509971	0.156690001	0.130129993
0.201159999	0.878840029	0.066170000
0.831830025	0.894039989	0.043140002
0.090200000	0.820550025	0.019300001
0.985700011	0.823080003	0.075479999
0.409410000	0.883409977	0.060210001
0.579469979	0.935540020	0.034929998
0.425999999	0.113470003	0.056559999
0.490610003	0.711589992	0.033870000
0.812770009	0.358999997	0.101109996
0.087830000	0.654079974	0.116410002
0.499330014	0.361919999	0.128940001
0.549480021	0.536109984	0.139119998
0.232470006	0.451290011	0.040010002
0.862079978	0.512099981	0.023949999

0.492909998	0.047540002	0.030260000
0.944429994	0.412090003	0.025720000
0.052000001	0.473049998	0.025500000
0.219880000	0.533770025	0.036570001
0.313080013	0.642359972	0.990019977
0.415540010	0.477670014	0.104719996
0.712090015	0.528280020	0.035739999
0.723349988	0.386810005	0.995029986
0.596090019	0.434309989	0.994710028
0.910290003	0.996550024	0.005680000
0.776130021	0.955699980	0.985130012
0.257719994	0.070589997	0.995119989
0.103620000	0.046769999	0.997950017
0.771780014	0.783729970	0.998319983
0.864350021	0.253569990	0.063189983
0.411610007	0.271730006	0.106600001
0.870549977	0.303550005	0.051640000
0.533020020	0.652620018	0.992079973
0.618040025	0.319779992	0.101480000
0.143649995	0.638159990	0.043669999
0.866569996	0.277770013	0.906849980
0.859780014	0.215320006	0.868969977
0.572650015	0.548959970	0.926220000
0.535369992	0.606739998	0.032170057
0.685299993	0.569739997	0.995169997
0.108389974	0.068340003	0.972630024
0.007439971	0.119690001	0.093129992
0.014289975	0.216570005	0.026190042
0.699410021	0.499110013	0.102120042
0.565469980	0.440670013	0.067720056
0.297019988	0.382090002	0.999080002
0.871580005	0.642340004	0.970539987
0.735419989	0.756179988	0.961000025
0.901839972	0.216570005	0.026190000
0.176960006	0.244080007	0.984830022
0.497079998	0.300489992	0.928950012
0.418700010	0.787609994	0.932560027
0.145260006	0.732710004	0.940509975
0.754119992	0.277770013	0.906849980
0.628589988	0.953090012	0.879440010
0.475149989	0.150150001	0.897610009
0.053399999	0.851230025	0.931710005
0.173800007	0.572749972	0.859770000
0.460200012	0.548959970	0.926220000

0.913129985	0.447840005	0.853070021
0.550960004	0.404220015	0.838079989
0.203140005	0.124810003	0.867410004
0.969210029	0.156949997	0.901629984
0.800890028	0.849039972	0.887480021
0.393449992	0.915719986	0.803889990
0.945620000	0.023180000	0.871609986
0.194549993	0.987619996	0.865320027
0.572849989	0.569739997	0.995169997
0.717509985	0.542710006	0.835060000
0.151629999	0.392980009	0.802980006
0.251269996	0.270509988	0.844730020
0.899779975	0.649150014	0.836650014
0.690010011	0.074380003	0.843379974
0.404940009	0.643769979	0.833010018
0.130380005	0.705399990	0.803849995
0.471610010	0.296799988	0.759519994
0.854529977	0.327800006	0.790250003
0.662479997	0.216600001	0.697329998
0.182899997	0.834490001	0.787710011
0.747330010	0.215320006	0.868969977
0.504059970	0.783649981	0.799369991
0.784500003	0.744949996	0.755400002
0.203529999	0.192440003	0.730820000
0.956770003	0.223399997	0.734149992
0.908710003	0.081840001	0.733089983
0.333710015	0.916800022	0.668500006
0.788869977	0.883639991	0.758350015
0.275620013	0.074380003	0.706369996
0.158930004	0.390599996	0.669799984
0.887520015	0.560020030	0.714659989
0.401109993	0.496270001	0.769119978
0.035950001	0.931850016	0.708880007
0.611800015	0.913580000	0.655979991
0.508859992	0.102830000	0.651419997
0.592670023	0.007310000	0.737240016
0.432300001	0.750339985	0.657410026
0.467390001	0.313190013	0.624779999
0.119209997	0.718039989	0.671729982
0.812170029	0.302199990	0.619830012
0.837660015	0.038640000	0.588289976
0.274809986	0.010830000	0.578459978
0.897669971	0.424809992	0.668770015
0.568040013	0.653110027	0.714190006

0.607580006	0.439359993	0.698880017
0.144950002	0.578369975	0.674109995
0.183329999	0.142949998	0.565419972
0.821980000	0.873889983	0.596570015
0.903900027	0.164450005	0.583140016
0.236269996	0.262340009	0.622430027
0.885869980	0.734420002	0.624220014
0.472680002	0.081620000	0.498230010
0.016249999	0.892009974	0.520020008
0.587899983	0.957180023	0.528100014
0.217360005	0.835470021	0.583609998
0.598110020	0.209539995	0.562600017
0.716139972	0.117320001	0.475089997
0.436850011	0.788929999	0.533159971
0.820680022	0.992519975	0.450919986
0.178509995	0.957180023	0.437790006
0.763260007	0.839280009	0.461569995
0.418229997	0.910369992	0.421770006
0.046980001	0.020579999	0.342790008
0.224439994	0.089070000	0.433809996
0.637939990	0.038020000	0.344500005
0.243699998	0.560450017	0.264739990
0.487019986	0.496069998	0.372680008
0.480030000	0.379590005	0.298079997
0.849730015	0.498140007	0.243719995
0.679780006	0.582599998	0.196500003
0.102329999	0.496149987	0.186509997
0.490570009	0.520969987	0.255879998
0.231130004	0.340240002	0.249300003
0.923449993	0.640179992	0.257160008
0.171210006	0.670430005	0.204229996
0.700200021	0.397909999	0.185849994
0.370130002	0.422329992	0.171010002
0.444790006	0.619949996	0.132290006
0.972660005	0.321079999	0.251239985
0.747860014	0.253089994	0.135869995
0.314559996	0.753799975	0.128989995
0.617079973	0.767639995	0.112429999
0.829060018	0.703939974	0.147239998
0.894990027	0.119690001	0.093130000
0.354059994	0.246329993	0.134900004
0.822830021	0.847729981	0.055399999
0.230069995	0.145469993	0.094460003
0.995940030	0.068340003	0.972630024

0.261260003	0.918229997	0.069540001
0.191259995	0.493149996	0.062980004
0.586960018	0.499110013	0.102119997
0.801320016	0.547619998	0.042930000
0.086499996	0.820219994	0.068659998
0.501990020	0.865649998	0.050209999
0.498199999	0.096380003	0.026990000
0.611440003	0.978039980	0.012270000
0.967360020	0.456499994	0.003040000
0.674969971	0.420060009	0.969579995
0.453020006	0.440670013	0.067720003
0.252339989	0.603450000	0.990700006
0.455040008	0.683250010	0.997630000
0.091770001	0.669820011	0.069990002
0.530879974	0.340169996	0.086429998
0.876890004	0.352050006	0.063780002
0.876259983	0.955449998	0.979130030
0.197809994	0.034139998	0.982029974

Final state of AIMD in PCC model

```
   1.00000000000000
     9.8709001540999992    0.0000000000000000    0.0000000000000000
     0.0000000000000000   20.0000000000000000    0.0000000000000000
     0.0000000000000000    0.0000000000000000   20.0000000000000000
   C    H    O
    96   240   120
Direct
  0.4521365673677133  0.4634364461154112  0.5256297727158471
  0.9515468396029761  0.4615412825118175  0.5242070937402896
  0.2017078039048288  0.4636243247986769  0.5249761718881734
  0.7020098327315379  0.4621786834697421  0.5225809898892463
  0.8263514361636178  0.4964367625541581  0.5306061653790769
  0.0750177746011826  0.4972255588038760  0.5326493386696497
  0.3273130697504889  0.4977463011127013  0.5351381250260876
  0.5777319366744811  0.4978532966794582  0.5321892989412760
  0.2008530108442523  0.3985936319761603  0.4932201776261608
  0.7008147972019070  0.3962772301496871  0.4966415736340780
  0.4489180806413956  0.4001049812359444  0.4922973579387426
  0.9513482519016654  0.3967378277091787  0.4932476806091455
  0.0757753284888494  0.5691012476242230  0.5359097757385892
  0.8284733877084358  0.5679323017116245  0.5319203878644395
  0.5793864359099536  0.5688525256835174  0.5362859457254263
  0.3267462600205412  0.5698090077806021  0.5386308369412093
  0.3244905649869049  0.3719726551054700  0.4703511547503481
```

0.5760556909865905	0.3696518438368490	0.4730995215690122
0.0755316023185321	0.3726670480470600	0.4679166951040556
0.8257709572449209	0.3678887078209361	0.4741039912070367
0.9545723060064492	0.6031254298560145	0.5260294454875653
0.4541451049338464	0.6028168424372334	0.5296574908205324
0.7048105267382310	0.6023887532676075	0.5272161116092586
0.2013225808687498	0.6026574016873961	0.5293714681225309
0.5764853182804812	0.3233937833391499	0.4189541874813225
0.0773624676362570	0.3268747093181539	0.4135372070132399
0.8264559472546786	0.3215113907920036	0.4194546078025266
0.3247556778448513	0.3239664330939058	0.4173202349958227
0.9554079675619268	0.6665204299908216	0.4941923166098562
0.4543434190988448	0.6668205983153173	0.4963734235076555
0.7048800459326328	0.6666174373731557	0.4946233900892202
0.2026390537449107	0.6658351857843914	0.4959109747757047
0.7023232533899492	0.3010166236908384	0.3916823326310412
0.2022263820905423	0.3038287307647334	0.3872501319564091
0.4523281405712121	0.3038360096404912	0.3892199801323993
0.9515597776878854	0.3052559293341300	0.3866639421078016
0.5799569284679492	0.6933641330113894	0.4722432326358831
0.0788414841717113	0.6933549414537026	0.4722003063208738
0.3271847668387198	0.6926499854678274	0.4741633394966090
0.8296354786319571	0.6929399275976496	0.4704573958669794
0.4555431614646231	0.2611803646990995	0.3318125379291619
0.9542350129603711	0.2669896859536698	0.3263773021353070
0.7038248587884666	0.2624442295324362	0.3309225396443572
0.2025252818502275	0.2604451125802891	0.3312987837915729
0.0796325129314112	0.7424173942651193	0.4193676260755989
0.5796673129766089	0.7416957467231787	0.4207808866057433
0.3280437035874424	0.7408177017446438	0.4216770913282187
0.8296702777248190	0.7394466563064627	0.4173927957584619
0.3302156246206929	0.2387883352385174	0.3047549115677988
0.5789581495526088	0.2431372778472595	0.3029041626339104
0.8289106964226879	0.2445848175072635	0.2994954846507132
0.0792409717686544	0.2419269208058393	0.3008865074761162
0.4532459049550554	0.7631304751295152	0.3942030105463948
0.9535993339728597	0.7634048797436903	0.3918694590745383
0.2046707030571366	0.7636898958309195	0.3937010819214816
0.7037915313976574	0.7602267953687182	0.3899916774688428
0.5793874774584559	0.1944742375736079	0.2496074662836820
0.8296226976170321	0.1965363349622612	0.2468933792769466
0.3297386367037967	0.1910964848482401	0.2522740456926736
0.0796745716210710	0.1948472041398429	0.2463780968184655
0.7036695039585914	0.1674239497701498	0.2268319402232974

0.2046375206433283	0.1646715883211920	0.2284829997352185
0.4536687295630518	0.1653622767407944	0.2283818152579037
0.9536485012560348	0.1695953387437176	0.2233376442985517
0.4546675379273223	0.8037372961082804	0.3361967276233720
0.9543742858708101	0.8062834098983422	0.3361370056210232
0.7048719539374629	0.8030639982581748	0.3338418372375733
0.2049043439350837	0.8069565931510995	0.3367836657491992
0.3304954152094018	0.8253138326464390	0.3082517221303097
0.5791258747239562	0.8249892467501485	0.3074269446074375
0.8301420823567327	0.8229975250204385	0.3055214209130113
0.0791751792347369	0.8266258825149611	0.3080108185243058
0.2043286960852881	0.1028489378452341	0.1932363438523157
0.7036908412290780	0.1027207121142875	0.1970122913366591
0.9542963158619050	0.1042279323737733	0.1944422182012712
0.4521220297335898	0.1031495387195684	0.1953438026693712
0.5779356494789166	0.0684111561664016	0.1894493577859419
0.3278236313509817	0.0692636759155693	0.1830075834009030
0.0777941611679386	0.0686138542831723	0.1859310412132783
0.8290269784722173	0.0692355676239750	0.1893557701994388
0.3277976391348045	0.8737543055897786	0.2554984927305118
0.5786051104374560	0.8743240689858144	0.2559110899526851
0.0761255169463369	0.8731888710274690	0.2548875526471545
0.8287460724648078	0.8722366703878121	0.2544075548090461
0.7044356125903880	0.9010228900611956	0.2349096181068042
0.2022301530969292	0.9011053683518961	0.2323087121450463
0.4527632383514223	0.9026806692570938	0.2342395491393826
0.9525300614533052	0.9005565102253614	0.2323425570736835
0.5820348595071189	0.9971838728787595	0.1919062882877967
0.3278456021731598	0.9970584493741752	0.1850501678175881
0.0757475349526064	0.9968932857053577	0.1879322885311561
0.8273876884974150	0.9988248132241455	0.1943383463092480
0.2003093167227087	0.9627033410572103	0.1954140278758965
0.7051062945697191	0.9647137563630646	0.2042694648645864
0.4540468809555473	0.9640890218025183	0.1999279851321620
0.9527691619758039	0.9637839724841943	0.2016351155435409
0.7687086058914910	0.7507021283489742	0.0348008984417572
0.4800188041268028	0.4353285202627662	0.1009157585658943
0.8857058145354908	0.7240443147025408	0.9905975047557904
0.5580745703690324	0.4402025263976561	0.0238803610088445
0.4617484164127512	0.9894693107966211	0.8861220939956737
0.0761414806728617	0.9395645700736786	0.9053140877573095
0.7907261569723387	0.8048903028031380	0.9441647127629411
0.3897929022283314	0.7526669179738136	0.9872174179660207
0.3522075746522161	0.2215185245282380	0.9364720586151434

0.5215888859645431	0.2392211866736297	0.9900515492483466
0.6182499982984562	0.3154320352229889	0.1409983439491681
0.2317169872670827	0.8357548184979467	0.9908647439086286
0.0446137402347619	0.6547033145794974	0.9811006803172493
0.0602515495350970	0.7261549611315405	0.9340011378817836
0.3665371767171155	0.8643566325567742	0.9932799687604372
0.3543226748727448	0.1446873125994765	0.9196082760389904
0.6068704326587188	0.3112297475733914	0.8273898972025046
0.8612867515611938	0.3653591959388535	0.9543523885065570
0.1392566889824991	0.4202354737497082	0.0032520182172344
0.6793952077345877	0.7243540614423747	0.9201739401473904
0.1538977498785601	0.0039867442083627	0.6674971266442340
0.2154558522973974	0.0023627952557640	0.9874511928532913
0.8624338552200491	0.8746434635707354	0.9136251635735333
0.5703643981606354	0.0431704040537221	0.8898624959149196
0.0449049316680128	0.8955089354750393	0.9678114698896672
0.2945506894577057	0.8847506648687126	0.8890907172446317
0.3828823275548224	0.8689703004938621	0.8274950116526083
0.2546919477584464	0.0881273264140643	0.7978767551037411
0.9857292676157099	0.9529570870399601	0.7968057399047559
0.8549314134974875	0.9765391755787889	0.7599842772558805
0.0040957023266851	0.2751834811390252	0.9182626739376462
0.3666853716723364	0.0487323744978905	0.7613349418472185
0.9149992065936610	0.2599453098877892	0.9816506375540214
0.8970494983927697	0.6346019267058423	0.8496583139158501
0.1622015473289371	0.2342916865589625	0.8655323689306185
0.4820589738752090	0.3672081141034885	0.8410651047006581
0.9915357393937264	0.5393807015212285	0.8602247892693723
0.9396903117188816	0.4344440454575477	0.9663440408727805
0.2908373628530661	0.9710809314503811	0.0507204916020435
0.0178088463373468	0.0258304606757912	0.6440673205621341
0.6221791577075743	0.6385873658111501	0.8444412287765102
0.6025384184878833	0.6155223051118153	0.7729008496510038
0.1654471248265705	0.4167815364661941	0.8381366064156432
0.2648767126964077	0.3984727116930120	0.8946877997215799
0.1452921877739758	0.5422037712347858	0.8660069472803510
0.8665143588779232	0.0277886102293696	0.8680530309559072
0.8551727397541092	0.0931753006763483	0.9209068577525276
0.4998032218531214	0.2884004888706365	0.9329892184027250
0.7481625693733854	0.8315330279155230	0.8469563981615140
0.1988610836344440	0.9113174366056974	0.8047991328563389
0.7707045178488129	0.2169127197841377	0.9112971302624343
0.7916239352992689	0.5833557759541338	0.8494849257333099
0.1472493940471293	0.3112692874173080	0.8570143004656700

0.2876035332118510	0.6654543162933351	0.7871830670555767
0.3522364971636837	0.2822136975503239	0.7503632032423544
0.1089418321120652	0.8059369210878705	0.8420392606395177
0.2425197685911976	0.7207548676704250	0.8418460813713133
0.3894633670888203	0.2231794323415214	0.7919627143194372
0.0391268694409556	0.7368059690537294	0.8210782743398035
0.9173105031272830	0.4426042554266171	0.8292679316654442
0.9895204606577511	0.4092699263237598	0.7709466779379315
0.7125978568135622	0.9893463286846487	0.7000290468542119
0.9810638287045562	0.9177527515462157	0.6343168031346172
0.7910935380072994	0.0947849561800624	0.6429271752077322
0.1462886013364926	0.1380784750622024	0.6894399252008295
0.8048262712164609	0.3330483349911590	0.8045901913800818
0.1202190305272750	0.8724280138269399	0.7414237456287065
0.7381090396482065	0.8615613066931237	0.7739606125288204
0.5148198947549892	0.1119663128503012	0.8223085810260004
0.8344149932838497	0.7351121745359136	0.7897335198494309
0.0293470460117287	0.2441152625108703	0.7926703365024546
0.6353159767865016	0.1377500624205758	0.8713101601017427
0.9141514478556642	0.6802493407789794	0.7419656716970752
0.9636507683403325	0.2441823266535582	0.7158231913318015
0.8069194680246876	0.2588955367926210	0.7876645922867272
0.0683916540096658	0.5253905262508406	0.7633074526899273
0.3691291088582579	0.4015910895931366	0.7076467656337758
0.5501829059236647	0.4770620825156892	0.7117401833022829
0.5651394732632978	0.0948487895124847	0.6948870984975984
0.8392381478186759	0.8989626112550283	0.6629451863547592
0.6095075114946701	0.9236994834228258	0.6977881127183475
0.2667597303156356	0.1907734963588044	0.6978592704470130
0.9048586172428262	0.1282696430397108	0.5984384333710545
0.3620261105995753	0.0343460982047731	0.6698503545440320
0.4519651862798176	0.0863575429648581	0.5935640750121722
0.3561499171303890	0.9590097498522897	0.6988517940357444
0.8061540122592138	0.0494193292083144	0.5564460433218905
0.3488143781791764	0.1282698304573867	0.6425939332917108
0.6670305951506977	0.0183900103033466	0.5442066039939182
0.5616590353593537	0.8224345095440936	0.8043003606969817
0.8308181857598741	0.3207637506931769	0.6549908938300886
0.6583546218066604	0.3207165676979856	0.7169141579120859
0.3325520592445227	0.6546871744337890	0.6676656387835455
0.0706107057847835	0.4876358543216953	0.6944039339963783
0.3662286862525442	0.3474015592423270	0.6443064464100904
0.4591237575817428	0.4739782836345136	0.7797808377835742
0.6669352111613045	0.0935547956587092	0.7546378886489246

0.1458867539908096	0.0067663761597268	0.5525593077224894
0.4300414354271846	0.9704562750622817	0.5369953716328294
0.4828631359613724	0.0337309578494336	0.4947780586960295
0.1776890846873358	0.0773421624130450	0.5210974440986785
0.8561243274626235	0.1935579943441813	0.5138312933789796
0.8256270694644691	0.8392973978341355	0.5828138994040567
0.2758328546385284	0.1956063370910406	0.5134328236560518
0.2924828135923169	0.6896045451463555	0.5970491331317439
0.6493633113695771	0.2958236128122598	0.6406643773742662
0.9144717287965879	0.2736110298662154	0.6083421363720114
0.4445562749814927	0.7675856406026905	0.7987764131641879
0.6188817801047746	0.7281362234906118	0.7183156232590145
0.5199833479288011	0.6881171975241381	0.6719730949950508
0.7027590332571250	0.4257385513746244	0.6742363874269928
0.3614246305114876	0.5372843460803303	0.7119749805498123
0.7819546916154034	0.4613742852855928	0.7245824290965898
0.0288866166844008	0.3670137475103213	0.6578143248927789
0.1762222723828457	0.3838370375242581	0.6788834766593498
0.2188122588602665	0.5573278454815594	0.6954151879241657
0.0727611456320802	0.8667114934973958	0.5037897758510177
0.7631692356091517	0.8740778465673777	0.4796281518465413
0.7217523776755946	0.1062739880811990	0.4958747370624208
0.7988909531737142	0.7790670273331928	0.5303275820727471
0.3282248940578071	0.1412487793439513	0.4646560276438826
0.0148695548048632	0.2080087455957617	0.5200212004338287
0.9341289004033421	0.5908484961273838	0.6763110959947395
0.7911126987295469	0.6376507456134600	0.6688820446254534
0.4132196927773199	0.2421474849274853	0.5791114867780021
0.2771614080084689	0.2349450905711242	0.6104787710416307
0.2735611551570669	0.8928825055100521	0.5406208420180409
0.0351146797938758	0.0593938285588195	0.4030853628602091
0.7136366267412736	0.1609683965498134	0.4356627651766620
0.0567446372939926	0.8306754525819910	0.5756536058829115
0.7600700769750222	0.9516746707872370	0.4776036849850001
0.2245887274733565	0.7576528569821965	0.6699513455060579
0.4601555128578155	0.8566502029086305	0.7279225310902037
0.0689879213475463	0.7613154238329632	0.6815813401122827
0.6414383061582504	0.2023461230682106	0.5485144396725856
0.8340952802844835	0.0977830556491439	0.3735122388862366
0.6893475868572685	0.0642102461742882	0.3784056410273689
0.4632498701486585	0.8689582595286987	0.6467663726874096
0.5781899798028325	0.1853078110061288	0.6154226665930667
0.1099658252815649	0.1214419964427106	0.3765505169965901
0.4284967246545847	0.8672410423801457	0.5442686170194931

0.9541757607103513	0.9182817388279091	0.4341686047878100
0.3592158647411861	0.9414205570660295	0.4484078177591483
0.2890383099589525	0.9395366773562062	0.3824965458190585
0.0657501499973591	0.9719141545037580	0.4622708867840051
0.4505135115345370	0.7702080749066396	0.6027040981798710
0.6016086932409610	0.8001604571452576	0.5771038985785298
0.3153224877589278	0.0487880478416389	0.3917007138465017
0.3953468087934661	0.1087496065887155	0.3586525004636998
0.6792264696878124	0.9492886823815686	0.3865236116475532
0.5341692452922644	0.9734454034529832	0.3695451636152138
0.2379440931327352	0.5022473893926298	0.4215720747230606
0.0719099377101295	0.5414595558710235	0.2827034381895800
0.9857291696613121	0.7496366784423171	0.1617520104373714
0.1075613261634131	0.6166391451937687	0.2810928252055155
0.3508378933298776	0.4587427339812167	0.2516801485569763
0.8613439997418696	0.7786200951867526	0.1158357041045390
0.3185009653716400	0.6261623243285541	0.2079713838305758
0.2016092678754003	0.5298295081683013	0.3536417633549923
0.7169395716119453	0.6765538932956008	0.1960809601574842
0.5839658134578410	0.5851595293405778	0.3290101314749021
0.7999987334080457	0.5213650431195253	0.0682787204209646
0.4216059591740192	0.6279720641485874	0.2628384248877010
0.7781930541630450	0.6290407336707790	0.2564156639864053
0.7979755738772052	0.6204048145013659	0.3713471935595561
0.0434199001608909	0.4123082073602104	0.2266408141097643
0.1334660753136381	0.7125339373071452	0.0629932752002628
0.3266222621948376	0.7681229897513709	0.1125966805330985
0.8300593851415543	0.2694120171623965	0.1167939865392931
0.3103474369326575	0.4153467292025382	0.3112849994107924
0.7613013167590730	0.6445494537815724	0.0165665742386937
0.4891368261548216	0.6822304037370739	0.1814623826500486
0.5762767170220654	0.6814702346009945	0.1102238897842652
0.0677234461796282	0.7229069873608354	0.2687740682873070
0.6261471486491346	0.5038480782833303	0.1438497389878191
0.9407530100120182	0.4577159371248716	0.3117997946159772
0.9373697578887019	0.6933868077375770	0.2352668753305426
0.7914542517015443	0.4542146553239191	0.3329178483041846
0.4893634555613851	0.5292569398963788	0.1722518800258228
0.5986361646384980	0.8107241184479176	0.1122636422008725
0.1723778698359247	0.1393133194143567	0.9556632133986671
0.0402267887745490	0.1376510377656883	0.7718191567803377
0.3144146905376269	0.0767782431827228	0.0150704845534264
0.0888272751942455	0.3849547072802020	0.2995117120532614
0.8989196521322413	0.2090066003516544	0.0837445448191534

0.5000974991100866	0.9154328824223702	0.0440887755155765
0.1317824899450761	0.6997430592522614	0.1392113333993402
0.3581417245775120	0.8181849387258793	0.1738568516593726
0.3095199638550747	0.3695000748040834	0.2092869514812779
0.7068892251594120	0.5655115191460682	0.9954832204658625
0.8228030274264790	0.5515061761742622	0.3301514443695659
0.7913309044510145	0.4449980720650528	0.0819537001344447
0.4678017148743712	0.5293829121046164	0.3355696391903998
0.6994159623375754	0.9279992014751921	0.9734315973775446
0.1150209603337929	0.1955143840785494	0.9918747381551938
0.9432600554884459	0.1331954975015329	0.0232703614321551
0.4464085947724599	0.0514176555837217	0.9738986635391242
0.6917229114384255	0.8455482548865539	0.0545054809458579
0.0586108546603531	0.8905450653718650	0.0859561444915035
0.0410952068907793	0.8128212023727196	0.0645349553836010
0.4051536738879965	0.8661288847325583	0.0856718694606323
0.7330598599031805	0.9415439194188634	0.0471522038217963
0.6559397099035199	0.1494948295900703	0.0729485985501608
0.3087526811117921	0.5891286231995833	0.9462420749653400
0.1069959760318760	0.3619663872810213	0.1540588651593463
0.0563467528201272	0.5934878936806546	0.0759157856223723
0.4707251275023254	0.2906838418950441	0.0657756820022372
0.5817918821486407	0.4093759848451626	0.2821687109093462
0.9151648467885836	0.5133019335627197	0.1662158848624620
0.7335115625478983	0.4500034227161698	0.9521697026297050
0.5096216238332084	0.1309339296414552	0.0402863337645552
0.3425986142827577	0.3952062199166894	0.0147372229097659
0.2545803023750545	0.3363992083823483	0.0020870205465436
0.9039460289449238	0.5835309789652866	0.1831571869691662
0.0355493933886948	0.4266294993988012	0.0641710115590623
0.2570330232973560	0.5308274736402711	0.1202983931193904
0.5809677028233794	0.4627845495594298	0.9266988672013062
0.5389923173566248	0.5282994187713685	0.8500926172154210
0.3968021261142275	0.5025506156229316	0.8748476912252037
0.8805054235943720	0.0020911739445131	0.9987646858808039
0.0099782797928050	0.9922541352028439	0.0426479640879831
0.2351643983748426	0.0430721649511493	0.8712908622846273
0.0854654512198886	0.0473387722117006	0.8976319005731102
0.5272152686283211	0.7491938554845472	0.0253050441845483
0.0967619894241412	0.2635233435148725	0.0818771158560989
0.3181872841386960	0.3134665868170849	0.1566598243896874
0.9505104739872070	0.3570357100233824	0.1425603618072952
0.2964125763456282	0.6305993120546586	0.8786806435694459
0.5056073312073159	0.2254897979028045	0.1053698072259369

0.0576151307799323	0.5332865918625407	0.0215016919538643
0.7168870670261849	0.3368161250384654	0.1920484311939522
0.7326575194570528	0.1626644269384082	0.9685318981358003
0.5301022208859305	0.7105286602229292	0.9140141456742271
0.2872640095111278	0.6274951244671640	0.0556052708813231
0.4415952200976897	0.6201100216934072	0.0408247652278278
0.0190184390655028	0.0585182585264788	0.7688175681355378
0.8467663416691493	0.0786566377888041	0.0438524836600801
0.2497489442046030	0.2431094809140174	0.0810829053385114
0.6540516729959183	0.4517133577003403	0.2377589219523121
0.1554566215076166	0.5426695928959009	0.1782731250419474
0.4871103755209856	0.4129201682347517	0.0552839003705418
0.7813216344974256	0.7298359690135104	0.9918566425709910
0.4659969470835860	0.7256024318887579	0.9997938533407820
0.1779286362815517	0.2599478676448365	0.0495978761509833
0.3086746678155801	0.1875050906161949	0.9073717181621266
0.4596294257028973	0.2714982464554512	0.9733644615572938
0.3054694064357181	0.8330236091810742	0.9622019085342622
0.0322279843914158	0.7060855911239614	0.9794865952121675
0.7128434474965717	0.3242556619791913	0.1466109546609188
0.7818261174129906	0.8437771770509588	0.9134171828367861
0.5231503353034669	0.0096393367627385	0.9210077993395172
0.0202974286978420	0.8994206098416649	0.9213710111329861
0.0736305034969387	0.5119048078072950	0.8474349965066887
0.6105435222345268	0.7114435056803210	0.8849355670385878
0.8621768427363153	0.4128200245202310	0.9419792586044206
0.5249502502327782	0.3253781847110165	0.8547549459854389
0.3528984064285196	0.0764241905197138	0.8029606481715764
0.9105527135638145	0.2794857462463767	0.9399773586456065
0.9223874746716066	0.9935765705567524	0.7920897603559199
0.3325620454342528	0.9069958319913800	0.8494424026511656
0.0843651841496670	0.9915193069401880	0.6359315980884356
0.2105391762162093	0.9971045961985276	0.0373111884148517
0.3556200548002251	0.5928739066795995	0.0429367409299884
0.6051572204356281	0.5964541772745340	0.8173841626043198
0.2256645943236689	0.3830430698299463	0.8539753761236374
0.0947687306832996	0.2695259246823682	0.8473245060207043
0.8727721171619338	0.5933185597316950	0.8728473876037433
0.8995813376484235	0.0504305950544656	0.9103951214342083
0.3274230006801399	0.6959337345695490	0.8214833551845110
0.1106811997211514	0.7583233537989608	0.8538924844615958
0.3785279243200410	0.2345580786838177	0.7461572380127257
0.9020602630014045	0.4087908296546736	0.7932619927376875
0.5984750900827138	0.0992009467678486	0.8428247842666582

0.1065292345654730	0.8915310509454779	0.7874837222939012
0.7093106868857160	0.1787831102997155	0.9228225306159500
0.7243810235183218	0.8197217476974683	0.7968779255425193
0.9122430568691731	0.7033840786522939	0.7878461126958120
0.9721125042455955	0.2174953359063372	0.7568196906692227
0.7450769309296460	0.2963486343399567	0.7927364597210880
0.8743953125316668	0.0824610909708765	0.6173733242927703
0.7071212341056013	0.9387945755891062	0.7087873150344333
0.9072521592368807	0.8817759845740047	0.6324070358417218
0.2164580979516923	0.1628972343204088	0.6652485518576808
0.3297312879366993	0.3607225097102684	0.6903147912263736
0.0728303773897247	0.5347866988570733	0.7147252910843663
0.4555301589741336	0.4714412752251153	0.7289229449067243
0.3010439143673555	0.0031243913355805	0.6997153041536087
0.7465531982569321	0.0291881700419554	0.5190862415864025
0.4470912683880796	0.1100083386517960	0.6370673488343995
0.6684300345715762	0.0864977688691473	0.7023764495959490
0.4680505441729326	0.8136905427213130	0.7876045360450804
0.6540101736527864	0.3356834435019316	0.6692643747040877
0.3503036783631276	0.6976860460414590	0.6350854320193221
0.9264477236717565	0.3043729178763867	0.6463231229427765
0.1383408679240603	0.0321071744638873	0.5116980258138506
0.4757574934902145	0.0168614749663795	0.5380530783827991
0.0854342309135393	0.4016682459918228	0.6789204926495115
0.6012229881658299	0.6854921806758925	0.7017637850772364
0.7163694720286294	0.4705945114612412	0.6896049110765899
0.3078089806542563	0.5795026329070671	0.6968772189233684
0.2876998565070694	0.1457427368394888	0.5088063932927249
0.7529724898872581	0.8165120533727561	0.5551823607890658
0.9359283740091244	0.1945964266153859	0.5448878806312176
0.3244875119480218	0.2628379481937834	0.5772369412820499
0.8881231712997364	0.6365266411018458	0.6746704092099899
0.7173343650841119	0.1574492999230384	0.4849526128108835
0.1092871397382869	0.8305208449016762	0.5348813170658467
0.7898085665107323	0.9100413847896306	0.4507950963231347
0.1499117765021251	0.7903189084147767	0.6809237153957004
0.5906283083085703	0.2246488725799834	0.5862985214657058
0.7321057252074511	0.1058292980815084	0.3686390631157401
0.4643458653408699	0.8917246517796682	0.6905334804982185
0.0218416659866765	0.1062780623758556	0.3914997298729682
0.3617062145305769	0.9032767957213230	0.5236758350168339
0.0478426717901762	0.9308115085486687	0.4356320270995307
0.5028691584255305	0.8088726667484445	0.5887165323930971
0.3718994071489587	0.9586679886343397	0.4010265763450241

0.3145600118341462	0.0978843186587236	0.3871856024899428
0.6291159417061070	0.9843946139838281	0.3621792287533730
0.3879980586600751	0.6572772426779504	0.2274523218746315
0.1041069450794490	0.5766995690855899	0.3106585526892027
0.2618339753736887	0.4935089027337339	0.3738218259415211
0.7964065837224827	0.6585857070559151	0.2177907915121034
0.7844084720652730	0.4878751465938609	0.1053561664755770
0.7587231292830211	0.5944512786409669	0.3325637462347478
0.4892091840138317	0.5723872236315311	0.3154181409986588
0.3601523504091245	0.4142905684176054	0.2698665199863979
0.9530044087552634	0.7551205589451925	0.1146631137783138
0.0354587891924810	0.6891105044056949	0.2362721004400978
0.8766271747908921	0.4811418850262072	0.3450075299779022
0.5464594728892276	0.4900073026121864	0.1736219716137793
0.5803058777787928	0.7021092265289141	0.1530529620838822
0.0698708415100712	0.4258575235472095	0.2752481515065167
0.9114465931891287	0.2571487609414502	0.0933357503802772
0.1995425956206800	0.7050886029047077	0.0994002614963915
0.3899729036696555	0.7977453363741613	0.1337686208147144
0.7129039739366617	0.6010838980503119	0.0294527356893128
0.8475831390442636	0.1252165544452952	0.0297020994628999
0.2466938763476550	0.3377991077711825	0.1809711388065304
0.6798139758923503	0.8056295537207814	0.0860778128997950
0.0832621017569595	0.1503031088952476	0.9757165161296475
0.0824148215333465	0.0934065866753222	0.7594280335234634
0.3968045365795038	0.0890050552599120	0.9913745353851948
0.9771359040804823	0.5509204618644098	0.1757118378263240
0.6775898228870549	0.4126082077198299	0.2635145199219481
0.6468759298674834	0.4727643540688191	0.9639768544977074
0.0967022713471626	0.8548988364632568	0.0559319078962637
0.4078950881328358	0.9057281036357019	0.0563515942980622
0.5808460464060906	0.1696008933716852	0.0498473057394920
0.6597529823765377	0.9260202127335336	0.0194331834081719
0.2748147595350932	0.3813403723405670	0.9843957494303681
0.4817974995040565	0.4846524913394084	0.8546900791818602
0.2498244734716639	0.5294855806603259	0.1689596781989820
0.0577907867775073	0.4455871942350925	0.0182345307698084
0.2948690965156645	0.5852719642014566	0.8983537711866827
0.0557743124048529	0.5835027849006478	0.0270718351808978
0.4644041132422950	0.2714219566569989	0.1092834177565234
0.0228442047099382	0.3881562029418950	0.1423340274399640
0.9108946622887083	0.9916243937526237	0.0445667701001283
0.1559042419987884	0.0156714894507730	0.8902647582972173

Initial state of C2H2 migration on C model
1.00000000000000
9.8709001540999992 0.0000000000000000 0.0000000000000000
0.0000000000000000 17.0969009398999994 0.0000000000000000
0.0000000000000000 0.0000000000000000 20.0000000000000000
C H O
66 230 114
Direct
0.7364419213811857 0.1929657896055212 0.2205916298281819
0.4863457240054075 0.1930096483754842 0.2207949527812532
0.6113907101171340 0.9841494199256016 0.2222658528138040
0.4861545138894662 0.8590876717988547 0.2241553883258366
0.3610409295746684 0.7341866536602154 0.2242031579402157
0.2363004020211188 0.1930146855432824 0.2203968413720743
0.2359755438124735 0.6091165418789781 0.2205656677467340
0.1111036445207097 0.4842350648630830 0.2127019216489721
0.9861889015063168 0.3593065276325474 0.2146933280499916
0.9863227032455338 0.1929905771539416 0.2206716172887702
0.4865142786618240 0.1092212265671652 0.2213223845974655
0.3613038970989446 0.9840496686774328 0.2220868009733521
0.2361494616175007 0.8590893162376619 0.2219351759820029
0.1110087371357805 0.7342125932955889 0.2210548935319891
0.9861572669093870 0.6929461742522890 0.2209217678191389
0.9859002860606282 0.6092623167699875 0.2183977015505057
0.2362663422640703 0.1092160651914258 0.2213935713325234
0.8613748982048742 0.2342620339223015 0.2198418848892532
0.1112899417315732 0.0678751637030690 0.2218836338825125
0.1112985324849341 0.9841375386391636 0.2216099271558754
0.9863362042751904 0.8591374407422978 0.2212768060757541
0.8612679126750975 0.4842278064786681 0.2163359599200393
0.9863332055911752 0.1092286647888825 0.2217555651052988
0.8615323296208696 0.3180123490425556 0.2173941349559524
0.8609908403294306 0.5679600418229567 0.2193036912514222
0.7365555115672408 0.4429550759540455 0.2190408477848299
0.7365556072841547 0.3592411530129143 0.2183119691216952
0.9863658168402918 0.9428534902653174 0.2212531016589005
0.6114042780629524 0.3179584922078452 0.2195109451624688
0.8613841923884706 0.8179220302187625 0.2226072811244215
0.6114396455449942 0.2342084473348334 0.2201562679007517
0.7364803124228124 0.1092084942939784 0.2211960094021215
0.7362978161108868 0.6928422982249621 0.2243926054012283
0.6113155015358653 0.5678970893545987 0.2247305872012428
0.4861077309101648 0.4428780273081679 0.2208925101327147
0.3612215276192030 0.3179462266671360 0.2189385543302701

0.8614119125405069	0.0679210500217015	0.2214374220619622
0.7364655419248908	0.9428669918045700	0.2217885956180072
0.1112448791089477	0.2343477062060415	0.2195014269754554
0.6112310716832203	0.8178655786630397	0.2244702585922879
0.4861201139386092	0.6928784520575610	0.2252192747884812
0.8613341211401964	0.7341737292905212	0.2228284508080457
0.2362266395698708	0.3591542022456868	0.2164946310379956
0.3610587281491852	0.5678738559602964	0.2219993146835441
0.2362194821924017	0.4429528188363097	0.2151309933877757
0.7362937029946099	0.6090878144989350	0.2237343505549548
0.3610500831964046	0.4841225157121770	0.2193606276533776
0.1111854019084511	0.3180582543520730	0.2167475626002034
0.6115223869602535	0.0679366162283099	0.2215303898396123
0.4862780247802317	0.9428000019124748	0.2230562155644018
0.3610987695468315	0.8179705840635367	0.2236611006873234
0.4861164598365916	0.6090736408948236	0.2249943826776624
0.2358902115529594	0.6929257055719861	0.2225456751068315
0.1110132283160403	0.5679759231848605	0.2161425069557614
0.6114088416773927	0.4841756484578429	0.2222461179612046
0.9859652328091772	0.4429468588349543	0.2123243946230757
0.3614144783961200	0.0679583545267763	0.2214122933072588
0.6112308279930760	0.7341238323916457	0.2251471105884537
0.2362482114625324	0.9428258525823144	0.2218483561643799
0.1111329478266253	0.8179184994857082	0.2210673050627936
0.4861623992185669	0.3591358270620059	0.2197384706070371
0.3613868778156897	0.2342030252408981	0.2201328860663244
0.8615128826066477	0.9841412300304838	0.2212917630484728
0.7363528857891388	0.8591103850085443	0.2230269913461745
0.0483729706130554	0.3912912982130402	0.3602865734409534
0.0633025784094722	0.4609284996450515	0.3524176762010464
0.0400702802412682	0.3294051830302807	0.3672358423726476
0.0752550150924294	0.5232668971410074	0.3472110017524142
0.7633067231011426	0.0118628636667141	0.9503788937653525
0.0906098724895967	0.7322017116098229	0.9716221802721576
0.6834924126922652	0.7675185374159991	0.9180775778000342
0.9037768246217608	0.4097908644000197	0.9773444018284120
0.8669993808383472	0.8222856017117293	0.8766122944526759
0.8526504736313363	0.8495327635848797	0.0684673467423622
0.5638842229040141	0.1403969655440429	0.9727713306106356
0.5388593794970663	0.2951268192152890	0.9849503959639420
0.2962848811974650	0.1562202779930781	0.8739649954804185
0.1663262056830876	0.1724108631329915	0.8292824407255437
0.8173426875926402	0.0743905916509473	0.8462486087387635
0.3565491233567648	0.9565300564452272	0.8604063244545601

0.8323870558308587	0.2039868077375460	0.9885439909374412
0.3636381793714492	0.2997117526524357	0.8424454478183545
0.1879844696398930	0.3116643604686132	0.9018186018164968
0.8010996657915569	0.1384137902914672	0.9062562649636252
0.3278996184328080	0.8917615322659647	0.9151357428751986
0.0081997591182355	0.3570224450718852	0.9393505050744672
0.8561981693360581	0.4197991053706246	0.7974344186037519
0.1834013569114241	0.3896021019916083	0.6824748945465421
0.2234721477530158	0.4254090337593615	0.6087949920728177
0.1189745043443478	0.0779624890094896	0.9099009072597144
0.7586914202648750	0.7492951387388230	0.7373983426272208
0.5280463147187411	0.1999293197043905	0.8078754357171188
0.9497275954070956	0.0028935334638011	0.7863285345534888
0.2886261918197388	0.7728864327174993	0.9366614888698296
0.4809957172255552	0.5805478645962281	0.9580662121771968
0.6933352765508496	0.8810724626191705	0.8438004298923401
0.7768628228177613	0.5670086357015660	0.8662622564372595
0.2864901609847286	0.3715708940835637	0.9346984018072184
0.8865217247908833	0.0551235803002682	0.7254910822726803
0.4336408829608668	0.7744134187852688	0.9028667340152896
0.8297724723462773	0.7433243514633132	0.8070883750471425
0.6848245563790329	0.1873438899559660	0.8021240415839711
0.9832587469262630	0.2293875339317659	0.7766965738570600
0.4460412001755769	0.5302586735835200	0.8183417066805415
0.9626588902706136	0.0609740311675594	0.9189075678378829
0.7086997543918994	0.8992933722770515	0.9229419921496960
0.5402647424896890	0.6691067674360043	0.9580926511233464
0.8113298375497728	0.2946736940446880	0.8087366258706830
0.4248809198785823	0.7542991991511533	0.7994893157850416
0.2068235457216739	0.8108889696694430	0.7512616677742844
0.7456445751663183	0.6313590851822671	0.9212393981626876
0.0120108442030931	0.8844437059324225	0.8265101094451416
0.2537459705230667	0.5731681494279708	0.8739997628638795
0.2713234243186778	0.0964055988789819	0.7748833676216648
0.3918489985556087	0.0016446845548872	0.7533902907305469
0.0935340787241257	0.2071334064745030	0.7220503795077371
0.3631762034718863	0.2650272119755846	0.7676663911661182
0.2464660200762658	0.8367446617236826	0.8236203280299310
0.7497141486373595	0.9715023963295516	0.7692040840603486
0.3226658627257377	0.1262599452756589	0.7042780832718217
0.3809298998461633	0.9091194189220162	0.7678587508531467
0.1696500199628558	0.5549657137056319	0.9421487787497858
0.1196907521154290	0.9485020531432774	0.8495063548342321
0.5783246898765265	0.7290278303743721	0.8102457440671498

0.5927722285791350	0.9398851856378416	0.7662241759108507
0.4299046761597944	0.6236113245395342	0.8231738043226315
0.3846927592106302	0.2175744024929757	0.6240825691801812
0.5753743729601619	0.7732202587672916	0.6532289019274479
0.8469707229791920	0.0646699730150610	0.6023945600333420
0.4260622450149914	0.5972999051177407	0.6849726135038035
0.2631945750349520	0.9096317846809732	0.6551505528069878
0.0634018534274986	0.5369001304453791	0.8362993378403309
0.5025820233835094	0.3676854814025124	0.6250381052101313
0.7967276835665185	0.2670808552772113	0.7358910541280032
0.0663153903318316	0.4697089932462091	0.7806843328276549
0.4561442632087362	0.3207980352040120	0.6875310738571010
0.3963397886351993	0.8486284563196399	0.6593194920723751
0.4976601756704977	0.3815323418133700	0.8112726558895350
0.4880616462736623	0.1868645333543726	0.6762199334749889
0.7543336011385290	0.1045734747776171	0.6600825717343294
0.3652717767443970	0.6331798056165023	0.6179466651555006
0.5296142415798754	0.4715183815837228	0.6970051029509419
0.8025001017863077	0.8449946259370879	0.6391350853109953
0.4490527241592486	0.7245783016852879	0.6772583500779502
0.2014465711749903	0.6302237769511554	0.6943507843955710
0.0072536217265018	0.4167187739298866	0.6239168093078949
0.0768412617157348	0.7281198403808014	0.7082189725949664
0.0860628785100316	0.5925520437456420	0.7421289255227944
0.6639309944987294	0.3404685109878688	0.6763912999902961
0.8589233208946526	0.5495143726363889	0.7755133924580176
0.8147397544389903	0.3664136187078292	0.6561181888078623
0.1160500119254831	0.3267180368744098	0.7689789539527742
0.7594262967669984	0.8685809121373421	0.7134163626027518
0.7651844149027075	0.6260396425734586	0.7751075440763003
0.5945418076332055	0.1150658129672802	0.7333697006630530
0.5049117232539664	0.0312953605236938	0.5597935681195919
0.9901042586713296	0.1468560100461517	0.6426992311287937
0.9831884720032612	0.8050652931778446	0.7059581601695722
0.4348410031422135	0.9699433967420352	0.6136947363486353
0.0912860066779644	0.2088277381174068	0.6067349886490599
0.1092928437360131	0.9416457786538360	0.7287249876162015
0.1808271144420796	0.0105384446557512	0.6933255909020338
0.6312925087418996	0.5354303680103102	0.7269748681821342
0.2835869630450287	0.4448384695647979	0.5062733533552202
0.4475362455749146	0.2705761376697070	0.5368297714361758
0.9207288420011820	0.9957085729270024	0.5224573513520172
0.5482304332148480	0.0734913048015055	0.6610487979194823
0.9174599416916638	0.4934596305778777	0.6201725964748467

0.9760886765267214	0.6132061017293090	0.6511676659228732
0.8040568520045903	0.4328375374982212	0.5505756364312383
0.3082806095229881	0.0578683222123951	0.5776582973773446
0.5564708572774693	0.8973895297203153	0.5098276652983631
0.0850082681252022	0.8078164008033231	0.6183210881900073
0.8314314177974398	0.6032158360044394	0.6088021087723248
0.7141338827384617	0.8801131657086527	0.5167539032630528
0.7593026205533778	0.0025275328034169	0.5161407424228758
0.0859325685104184	0.8640648813488541	0.5510506108866913
0.4645768332516129	0.7876334507641893	0.5253592834092831
0.2999396474109300	0.2358666624215777	0.5301749478166997
0.2208807851246199	0.4224271849588905	0.4061271343455939
0.3696710091884549	0.6239015166169848	0.4915612848897501
0.1686351715742308	0.1024095870244866	0.5932253118107454
0.7423026296660099	0.4259639266900004	0.4750155751850745
0.8360518215339308	0.7942691011667499	0.4742494597482163
0.0800918047177740	0.7242118116816431	0.5358688098218019
0.2333747938036243	0.6383119344691734	0.5327962353824209
0.7715368607016269	0.1800283528680669	0.5741080773482400
0.7467478365686726	0.2650377558816378	0.5996944262994356
0.9086292798680088	0.8061221131605789	0.5455097518544979
0.3716963147067104	0.8545622376690473	0.5597214465174934
0.0168243943660471	0.6383577805231314	0.5496883847076358
0.1436422846022667	0.3165697713029840	0.5761688286812263
0.1322304407619767	0.9915628919549324	0.5285085374539612
0.6161086845149840	0.5605831800765512	0.6196633022939947
0.6018278531328281	0.6658827893144396	0.5418926891790985
0.2859104158125866	0.3432899680819448	0.4244360514116198
0.0608083059753899	0.9494133530829628	0.4631084651128587
0.6823811905590862	0.7447210628124418	0.5291465572253966
0.6802395104687926	0.5369953378558554	0.5502102144669331
0.4649182244326657	0.0616316048417915	0.4497465809614428
0.4723054442723792	0.1362591120629116	0.4984428735766269
0.0608146238914170	0.2772982695553646	0.5138859844346046
0.1046533655006997	0.6504757578170995	0.4390958751947932
0.6860834544562271	0.3565692846577507	0.3922622310998786
0.2506960510592062	0.0767891365230739	0.4085959052554000
0.3570045569424359	0.8555240732839090	0.4416180187539313
0.8946745185852421	0.0920949442620783	0.4541016445642663
0.8295857460803464	0.5929778356051384	0.4269572663741035
0.5523962214560478	0.4683361633065021	0.3503409562133307
0.6276208720207916	0.1257707831745938	0.4194733225839226
0.4453811146353933	0.6661652385765288	0.3900994695445818
0.6832341744974664	0.9260981950534356	0.4227699868585671

0.2278360486596410	0.6227437888353790	0.3945585811088519
0.4413523863156502	0.5699562962869599	0.3947798040214575
0.6723636399741610	0.9515032730925328	0.3462376771395884
0.8301964637543442	0.3403495764065661	0.4213313709719603
0.6804592546551347	0.6219435841261930	0.4167683786256795
0.0301330523877927	0.1195114278939501	0.4177338429871642
0.2061485911450744	0.1063822890198081	0.4802342767304836
0.7623149660754827	0.1453699055024241	0.3803192075331588
0.2540378944531742	0.8395316254656827	0.3814689339415079
0.4369372917010805	0.4454861082279599	0.4020843644850663
0.5363809834447577	0.2947272555490682	0.4386019157359183
0.3716810463937157	0.2591696874663879	0.3765459171780040
0.8508164833991639	0.2594945492201759	0.5027358615863580
0.3568484537513940	0.9555739157105584	0.3853912495054299
0.9249982687504900	0.2190926459285067	0.4428378883776373
0.4033920787906520	0.0288390961011031	0.3401824671927721
0.5994855813389853	0.2368752246760681	0.3850776894445753
0.2497478593038813	0.2087262769136439	0.4058812597314397
0.0217577146759922	0.5540205204919171	0.4462649950152943
0.4750421426118163	0.7907214294058778	0.3805504539708952
0.0279013193122576	0.7977167294557284	0.3756751829676834
0.7816557354143475	0.8104009096139064	0.3754398059248246
0.1301873596026442	0.7288397035564806	0.3699151899509253
0.8171940211922049	0.7204652494338011	0.3930509401184093
0.9258716443809284	0.4835779878488095	0.4412682791205880
0.8984456516128977	0.9420952840122576	0.3814562309210680
0.5626943186653136	0.7429341651642043	0.4324426342995695
0.0049827241512198	0.0065847409802136	0.3680252588944415
0.1742442967698384	0.1794358261463439	0.0741872859938970
0.6126363894545228	0.5359156965490603	0.0908974685840480
0.9344854864820096	0.2190357516631496	0.0725809519837015
0.7432141387239768	0.4171630523973420	0.0620163470858977
0.7325546353752422	0.7350247886163106	0.1031484494060595
0.1989388134151396	0.9453712933531347	0.0647029096333201
0.7122801871724009	0.1502309967416445	0.0676213008954572
0.3724506368374165	0.2451071191050604	0.0513202082130347
0.3597665039190869	0.6590395256974200	0.0325654488265541
0.9348655163880808	0.1239362857264868	0.0667010485693335
0.8691535139155456	0.4758471514996547	0.0604164338519874
0.8015136377122892	0.5867830624991128	0.0571251854504315
0.6263498981518610	0.7942153782557301	0.0707742465468698
0.2537552260325349	0.1299161560919481	0.0207970130401313
0.3451905648681499	0.6772061303727029	0.1097465772946679
0.2179167908466800	0.4410698792455230	0.0317604437906458

0.5206995677914785	0.2241109715460206	0.0708744409471733
0.5906942263137304	0.0878163051871804	0.0749365930994772
0.2642376175741423	0.8625288626144406	0.0691566246023216
0.1276279560622416	0.7067368160245302	0.0653778829284612
0.5602708742770232	0.4379679669253195	0.0171482901920504
0.9550443145847016	0.3463385188005715	0.0746593554736922
0.3974708898331217	0.0783757966840480	0.9651367439045758
0.1046061120674138	0.3244240555759840	0.0558136853399698
0.2990662939385331	0.0067035037660434	0.9606228696613276
0.8758206652638114	0.5751214236305606	0.9886229815963408
0.5380014762706192	0.3694513229645995	0.0717208149915737
0.9826310712231882	0.6747966253321358	0.0483273867405795
0.3240659639246988	0.3784982018189960	0.0567520319419157
0.6069991627269993	0.9795092931124372	0.0319475112393794
0.4525349577568814	0.0047839070276469	0.0402111248894448
0.0478631741124451	0.0447688233778733	0.0165065145883554
0.9788579801732168	0.0004158407899768	0.0773673151278232
0.4934522191374890	0.8865953092018823	0.0666483835989756
0.0673486331139484	0.5438851007278412	0.0419767113389517
0.4460444078378841	0.8190204831986300	0.0163067307054412
0.2161144618865859	0.5745200103813128	0.0545003663074781
0.8151042446620734	0.9454647001921956	0.0040592347412726
0.5360583051086967	0.4575296936397514	0.9045122485259576
0.5367962043137211	0.2526259609699096	0.9187111826809000
0.5584529602046679	0.1302462569194910	0.8921016915686507
0.0100856595047727	0.7737834758594121	0.8800908054317904
0.7116463128109218	0.7350867054024033	0.9909997758961964
0.1433741493295659	0.6770903630866920	0.9125201590386186
0.7341544659050201	0.2581313912244131	0.9423805496133172
0.9792638551068502	0.2420839398661571	0.9285805997188508
0.0045225672344392	0.8768437278447760	0.0547004011978478
0.2021866796657257	0.3845308557620418	0.8126094848330284
0.6398104074263038	0.4188210624016396	0.8246641858410997
0.3172461104089656	0.5249479207750185	0.5465462818502567
0.4396886396656196	0.4056402561483524	0.5419987583712225
0.5988292380704454	0.3900095504208408	0.5358952350022882
0.5805277234918027	0.6060914944664261	0.0431646274896570
0.6426362754573306	0.5145434146840093	0.9375525146393198
0.1181485843022889	0.2260880151463920	0.9684455870453242
0.8603346757809479	0.3980469616492226	0.8742240952625744
0.8167690984540580	0.2234433939339681	0.9416115318364188
0.0794328056556041	0.2511332091063341	0.9280180374216708
0.5796771804267614	0.2984455992402451	0.9394812411790396
0.7348877155095048	0.9670090972790256	0.9791703032759485

0.5032801202948675	0.1403065503144545	0.9328518748246259
0.9099052631314924	0.8956383686641476	0.0583559172135925
0.9431288285010068	0.8003536246372255	0.8494461378940374
0.1312419312688046	0.7334978853623370	0.9251011324701948
0.6849476386547114	0.7179439285909336	0.9448733723497428
0.8053600774713001	0.3907216372654129	0.8309781298009130
0.8037833901051334	0.0810348917646852	0.8952109870404500
0.9564778145531018	0.4058956507901036	0.9348038749560014
0.2240446927937769	0.1301833551101440	0.8492194104631740
0.2930130267150252	0.9426665703722616	0.8969885593771582
0.4218572954627473	0.2766814422683025	0.8058026281662942
0.1618984671600022	0.3885828620440482	0.6341169003714439
0.0540216687578714	0.0467103517454784	0.9362445542150308
0.8724063798732685	0.0374015010138733	0.7722794153079771
0.4741149628996351	0.6327190068119142	0.9800356780229160
0.7742181024883158	0.5754459309364123	0.9156167888203977
0.7538964636978485	0.7202060061625434	0.7801152713481044
0.5978889126438595	0.1575588555294525	0.8088855022513968
0.3779246261064145	0.8010888163766362	0.9375141835722400
0.7048974717789400	0.8561924418768987	0.8887741968754312
0.2640006807582399	0.3486928672266350	0.8905958647553699
0.4855688118251467	0.7230353036495034	0.8285561949152481
0.3800913300833474	0.5731342009619350	0.8217338750439780
0.0559890217278351	0.9352731868315493	0.8130776620728501
0.2979046093409165	0.0780890790423821	0.7291544655962238
0.2867904433695144	0.8221164724762464	0.7808804097655351
0.0838014591572926	0.2291801870664838	0.7683624823663822
0.6909451510852703	0.9234939524117920	0.7675760772928236
0.4344906903989005	0.9570834044164800	0.7780652425449772
0.8159633287943652	0.2452412543193032	0.7817271997604293
0.1630169533880468	0.5786474479936885	0.8970557828783962
0.5043925562710248	0.3694110292597423	0.6756013476716602
0.4971028366141984	0.7760192929401059	0.6827869074742822
0.0249426196325932	0.5214035744997437	0.7917283350958485
0.8101100626602127	0.8283566553696750	0.6855039556289861
0.5409638699016802	0.4314930685449140	0.8229909409836093
0.5581096946729356	0.5268557251172933	0.6933419457926548
0.3997599576083379	0.2157479382350184	0.6737002643054699
0.8513253442887523	0.0946998655057912	0.6454506343674856
0.3495683641555523	0.8909078442601438	0.6331109095665438
0.3668122588537727	0.6399389235623463	0.6677255477354385
0.9104338820515124	0.4349547976293253	0.6204009189122071
0.7631953692367451	0.5672001341235253	0.7788654552910921
0.6045140998778351	0.1149599522392181	0.6834751843270866

0.1037569584792870	0.6337577203313465	0.7076009568454547
0.0848091108141506	0.1680260435322643	0.6427667488734317
0.4616117231903437	0.0257692073102379	0.6043053424815279
0.1345000825896455	0.9603084017681668	0.6834234085173607
0.0778694787612282	0.7864455562152337	0.7012202730120307
0.7584354807290933	0.3198743863472404	0.6689943367827824
0.1498586018836940	0.3821851058985239	0.7703061625109602
0.3085055690118964	0.4662902521077051	0.5513081258817101
0.6477613075477129	0.9222913212860548	0.5040461266611199
0.2965139312762907	0.4013898227087667	0.4330255181874851
0.0670560240692357	0.8104264139730856	0.5692189175583903
0.8428581051614530	0.0337395424171029	0.5232028679954138
0.4177945937784939	0.8384418925744773	0.5170609039219194
0.9298702325791957	0.5915329805699079	0.6109630177242950
0.2169847586573931	0.0704400508714253	0.5585984166143668
0.3959811960233195	0.2202838872065987	0.5380495238946140
0.7221946361597034	0.4428350970902871	0.5227080069367867
0.0701186395068261	0.9489338962155990	0.5131808546947113
0.5083895672089727	0.3626590003339948	0.5442142195208514
0.5230095506648834	0.0892589251048131	0.4827927230113827
0.3295414354582223	0.6185267210862473	0.5374005342048516
0.1373755128338632	0.2700190942637449	0.5463511927004542
0.7435476143648037	0.2316649885076248	0.5584511019193624
0.6602415679381476	0.5833101814734090	0.5789595797097147
0.0812265732138177	0.6683398952949362	0.5206534283659449
0.8169829608342128	0.8053462036553123	0.5235093491135060
0.5921487957029499	0.7196015284631466	0.5226006772768521
0.7714050272027948	0.3836302822188054	0.4046699653792811
0.7268535477061994	0.9215468904720460	0.3775296886202693
0.7714254379288572	0.6279235200303259	0.3989477087803040
0.6631302517808518	0.1506617847108439	0.3779812252700431
0.9284933262550028	0.1242680036215657	0.4159769875488756
0.3921505562500449	0.6197380845617422	0.4064943714784667
0.1267011861478998	0.6298759032974574	0.3935882534500258
0.5104042580501659	0.4840781861971170	0.3921533243600542
0.3437170893990730	0.8655242104529712	0.3926911462895233
0.1935541574549169	0.1173152947220558	0.4313212029144488
0.3734814898695926	0.0151369981058977	0.3850859821092175
0.9976631479611280	0.9529882480417008	0.3840849896383300
0.9193621130355462	0.2708599450355363	0.4661173233533658
0.5484858951253087	0.2881800218479953	0.3903817089028265
0.2729926520845826	0.2599029835154754	0.3852551448228637
0.8493996348841413	0.7763926221800811	0.3966512236742985
0.9400156056047834	0.5316713704626791	0.4668303062363329

```
  0.1248670391000366  0.7867084726933768  0.3687994015656195
  0.5388315720565260  0.7460209616165387  0.3844957539139773
  0.2272795887188035  0.1848011741539488  0.0333629662217471
  0.1872031083669917  0.8912291367707135  0.0504539717806788
  0.2928190633323287  0.6626516705794910  0.0704489212351159
  0.2490251986652051  0.3855603183300094  0.0257411801065781
  0.6443276500459653  0.5884692806360667  0.0790067116221602
  0.7232185518802893  0.7740462981802962  0.0678761374122399
  0.4612996369796483  0.2700723143704370  0.0612584468949932
  0.6141930889815024  0.1406843655254698  0.0581432156861212
  0.8762232652476367  0.1722929334258703  0.0630057438097212
  0.8444556068611829  0.4202270303175537  0.0559363833577493
  0.1632266158101810  0.5325286481155106  0.0317218552464082
  0.4742856914004411  0.8291322915822135  0.0634932186127831
  0.5327508196756794  0.9850062642545516  0.0659774940173375
  0.5544664257296169  0.4873411588328485  0.9466783718239972
  0.3316923499840524  0.0475440379422903  0.9930875897490959
  0.0201389937182365  0.3021440176434009  0.0741982489045835
  0.5772006513474417  0.4218610532577475  0.0649629786226216
  0.0374711396191503  0.7232600313572639  0.0489103805347737
  0.8957669792131265  0.5806073253499389  0.0372905183472493
  0.0334215521944098  0.0468670686741279  0.0666895588640346

Final state of C2H2 migration on C model
   1.00000000000000
   9.8709001540999992   0.0000000000000000   0.0000000000000000
   0.0000000000000000  17.0969009398999994   0.0000000000000000
   0.0000000000000000   0.0000000000000000  20.0000000000000000
C    H    O
66  230  114
Direct
  0.7341135607171716  0.1930390202701210  0.2208062517790140
  0.4841802690306077  0.1931629188586262  0.2208641739437621
  0.6090610264414711  0.9842111348815740  0.2218643042889605
  0.4840017912576612  0.8592304754006920  0.2230938538374607
  0.3588241795777055  0.7342545739599811  0.2231430857828555
  0.2340990027971371  0.1930182118899970  0.2220008300475377
  0.2336542646124493  0.6092023104097208  0.2223619568082587
  0.1090026167932703  0.4841087839373605  0.2211475250813215
  0.9840196047508424  0.3591724109818279  0.2214817185337110
  0.9838361789896856  0.1929949327908150  0.2228822058708672
  0.4844112515935953  0.1094007021807127  0.2211837240069678
  0.3588389444142661  0.9840169005029576  0.2216170231578257
  0.2338736413543475  0.8590274118926610  0.2209811590663560
```

0.1086894882723952	0.7343297929190197	0.2209362338344813
0.9840568990892128	0.6930512009690821	0.2209831194215313
0.9842121830567786	0.6093298971415533	0.2206357968802466
0.2341322077026650	0.1092273317753484	0.2220009079993438
0.8588857129385129	0.2343552554682055	0.2214668414270790
0.1089534144253629	0.0680088962197476	0.2223706996530516
0.1091145360065980	0.9842467192786252	0.2214951639022937
0.9839953572529384	0.8591977364982950	0.2204012193456375
0.8589408691040659	0.4841511315939894	0.2176840533212759
0.9837600963590184	0.1091828825213358	0.2229764123595100
0.8589069812418316	0.3179753542582400	0.2196995533413539
0.8592449319515540	0.5679611706484300	0.2197849193858663
0.7340461206388081	0.4429301544097209	0.2156644258255864
0.7340417207025933	0.3592701880222917	0.2168157454530190
0.9842422741181892	0.9429386370832128	0.2210323481943716
0.6090195052155483	0.3179714988202357	0.2180306962865202
0.8588100699962887	0.8180461623986615	0.2215185862259439
0.6092344101204225	0.2343254672084708	0.2197845830236082
0.7339894198277982	0.1092499845594697	0.2213756567157149
0.7340157232234263	0.6928834963250973	0.2225120575156079
0.6091033236441361	0.5679206549633478	0.2211165748925222
0.4842490877398973	0.4429242852415894	0.2192567612794375
0.3589360999642168	0.3179726528418616	0.2210998970336158
0.8586431590419736	0.0679219174556020	0.2220601322154111
0.7342798541932508	0.9429261863959804	0.2213327059751107
0.1090030540464810	0.2342378615258142	0.2228009127999175
0.6087966506510072	0.8179218549380946	0.2229969731942039
0.4839342236826437	0.6927586832767554	0.2235357276373777
0.8589483047398705	0.7342135435822125	0.2217614092847977
0.2341865510928748	0.3593645851872145	0.2220832690689946
0.3585916404005132	0.5679002720141172	0.2224394245111799
0.2343996061406673	0.4429865223578668	0.2215466458668092
0.7342268669065037	0.6091823966205061	0.2213784884371823
0.3593754162570396	0.4842579283414126	0.2211889742324925
0.1092507430575849	0.3180693785844440	0.2224436071590551
0.6092512329968510	0.0680216815446847	0.2209701023255838
0.4840556483064556	0.9429191213505252	0.2224619918492145
0.3589710957605939	0.8180723549493818	0.2227134521995567
0.4838181388424073	0.6089840907847496	0.2228291154451855
0.2335618650886692	0.6930071442254813	0.2225194002481158
0.1089662192562740	0.5678612990392603	0.2211297311774854
0.6092749606150084	0.4842849814375183	0.2176939861364184
0.9841144080604368	0.4427984168018727	0.2197128414822434
0.3594233638664657	0.0679812198533694	0.2213733029077728

0.6087519109949535	0.7340563536141888	0.2230787875910360
0.2339853066883375	0.9427943362997648	0.2213938412145146
0.1087374290618489	0.8179692761768750	0.2203323092111918
0.4840303844422568	0.3592156767299619	0.2191845939380964
0.3590370778059096	0.2342730671976175	0.2213868358175506
0.8592393212265628	0.9841457184418648	0.2214459145375951
0.7338836358134933	0.8591686568300910	0.2218759537486105
0.6713653088869090	0.4064702070505486	0.3659542359925813
0.7028553327115377	0.4745545985200140	0.3636689928123708
0.6391557356007590	0.3457074740757323	0.3681537756756726
0.7271493112635599	0.5362425839134868	0.3632384034908595
0.7634390543722596	0.0099010916315859	0.9504886872767916
0.0839373121576564	0.7301340948587506	0.9689686254348168
0.6796907931408473	0.7654799295529536	0.9178091527799306
0.9011032623898370	0.4089431839976767	0.9790165383094980
0.8642503743083537	0.8189836875499844	0.8734325974127242
0.8519957573877663	0.8481017201230754	0.0678796922749924
0.5603970837692154	0.1425356115298842	0.9725061103109810
0.5380821285657763	0.2927090374554393	0.9823510949769084
0.2994392596237698	0.1540434268409841	0.8787356054904201
0.1704571612679676	0.1751415495043293	0.8342062069428726
0.8157928219119889	0.0725094205124590	0.8475511514881598
0.3551655142130055	0.9561178814417663	0.8588399776578332
0.8301555921192942	0.2033979243872188	0.9894031907137436
0.3624254084932028	0.3020697729299514	0.8421656814849171
0.1859816964482201	0.3125876410521317	0.9005756760438701
0.7988426796728336	0.1369320164169918	0.9073157552472274
0.3238829575254846	0.8910515386662590	0.9126244523008942
0.0066805200171126	0.3572191013036543	0.9406537955124690
0.8608246964545850	0.4212841494196850	0.7990318683097594
0.1939669617545068	0.3954790174244283	0.6776226437247024
0.2193571005908777	0.4247624664846912	0.5994573972864845
0.1185623475954612	0.0775145430540357	0.9103659058769794
0.7535922428403711	0.7486807774097289	0.7337135580576124
0.5300822571397943	0.2005409109459629	0.8084668895907910
0.9488518857639214	0.0000631108155342	0.7880903680101237
0.2829707607889434	0.7713792013092284	0.9333380958830628
0.4705990395590486	0.5811761665615123	0.9563740188274096
0.6925517202540319	0.8788379190882245	0.8433466752201437
0.7719804683287105	0.5666356656297546	0.8633591993507516
0.2853068795096113	0.3707684225455918	0.9351758187098512
0.8852119642168013	0.0514974863126264	0.7268782611729918
0.4280036529361859	0.7725570921461019	0.8994180235312562
0.8267923115900940	0.7419126931740295	0.8029668919638023

0.6875290682390218	0.1879812064966623	0.8044344716749228
0.9887352113358492	0.2321297619647758	0.7811607161471186
0.4445646133889120	0.5290289015690318	0.8160151444019552
0.9619334987093668	0.0606069676958493	0.9195059533408824
0.7108328477841668	0.8970755424455195	0.9224609865809940
0.5307677865016399	0.6691929942907943	0.9565761007104008
0.8134617551556552	0.2958648721559983	0.8102199921172835
0.4257656168152008	0.7512601158173664	0.7959203391899484
0.2081604607877745	0.8094229230016059	0.7491381921807688
0.7403715185827408	0.6302130667372582	0.9193339103456400
0.0125323630025780	0.8809480676275254	0.8257567532672376
0.2521075147568877	0.5723283582062922	0.8713950147668252
0.2723652885931980	0.0972847987348306	0.7789298700146671
0.3924715828956620	0.0016029899623574	0.7542059409713591
0.0974223107858615	0.2062873104797904	0.7274563672651607
0.3717317295511109	0.2689278809975399	0.7667986323111237
0.2521096109203866	0.8305287988431611	0.8219978485730363
0.7491643647744637	0.9692942981393924	0.7702296294164580
0.3195152990049406	0.1263662206089868	0.7075432683598740
0.3803339210543850	0.9083305907638280	0.7668448080995376
0.1688423846505925	0.5547767544238031	0.9399073257618864
0.1235817396953962	0.9433852984783146	0.8496544601460452
0.5786542488721218	0.7258446284533545	0.8078025356830839
0.5915532774501262	0.9387395721837258	0.7661669837784911
0.4287160028676169	0.6223621648233415	0.8208509290256062
0.3845849815511967	0.2187646206552340	0.6243652905429271
0.5747225258418430	0.7694037299688906	0.6463839196677585
0.8500840710322652	0.0640162198576206	0.6044453206002754
0.4263122574985846	0.5942952152149860	0.6821358426219809
0.2639733539308609	0.9091558531105496	0.6559282281588829
0.0613234463017484	0.5350246131707954	0.8339028686266964
0.5031476932107508	0.3697300256284741	0.6150076859204712
0.7967834394227379	0.2649807594920977	0.7385645300825612
0.0691477430793583	0.4686006438799934	0.7777912570634012
0.4521769931620617	0.3218965267725272	0.6771612402978415
0.3955330832974119	0.8471786580001697	0.6584153714617739
0.5004910243074945	0.3802801688365463	0.8086077215804260
0.4857772847970563	0.1904554431165530	0.6778647090668151
0.7605160253161928	0.1059291979098436	0.6615931205452439
0.3645806023986239	0.6262332831716342	0.6142325417946152
0.5321580288601918	0.4702327215242338	0.6911483037658008
0.8067280345925614	0.8499143782857937	0.6376934896622091
0.4495320626079420	0.7212236412014383	0.6727054014285617
0.2019293094056359	0.6283269877291066	0.6923868628116946

0.0065957927293279	0.4211461296927803	0.6304053944371997
0.0765960719894164	0.7277297741450922	0.7072100431950306
0.0848224608906917	0.5913648852703745	0.7396532666055181
0.6599010772668901	0.3388747015374914	0.6744800645205038
0.8579318632561033	0.5483476530668433	0.7726973665908080
0.8133121532451529	0.3643452019517075	0.6611782672075814
0.1242283422591586	0.3295048319113683	0.7673061577517652
0.7584722551555848	0.8688871220626607	0.7120478564597599
0.7640900359960807	0.6249194911785915	0.7712566925396707
0.5996516662620405	0.1196303097422994	0.7330989280188540
0.4988627144301924	0.0323044886059513	0.5625223171076632
0.9932480564162116	0.1426852142768660	0.6492094580514600
0.9828907872590964	0.8048498811835785	0.7060929342767208
0.4343338303198912	0.9702371256953845	0.6172502474276375
0.0978826009742646	0.2049770703790247	0.6158435327249819
0.1117430647436655	0.9400009783056680	0.7312558684199114
0.1835284120823412	0.0096609907066603	0.6969444936120978
0.6328711114635893	0.5337486257258215	0.7231907927732399
0.2529792522052152	0.4345182019729368	0.4931951449705032
0.4494449268576058	0.2698947950320820	0.5347866223405637
0.9226068512364936	0.9944466100531696	0.5255180391717789
0.5516054200045997	0.0773507519061914	0.6621208227463633
0.9160871054958580	0.4974078134044356	0.6273508373229897
0.9819703774737022	0.6153099281584326	0.6469909214079725
0.8156155243050299	0.4294112515212844	0.5608458811296796
0.3058617087699652	0.0576993112671708	0.5803065906821208
0.5599297513946498	0.8975098442890906	0.5102616571264375
0.0839794022994150	0.8100360367867553	0.6192694723642005
0.8370043917395876	0.6051639642397070	0.6056452866361146
0.7172838526258424	0.8788180506403227	0.5152979661958592
0.7599951028760313	0.9979638875194130	0.5196903973826242
0.0854783285643284	0.8662616923269539	0.5517005554855849
0.4668251779535104	0.7867572660708221	0.5247726282697414
0.3012219557840997	0.2346649839465217	0.5336466914699325
0.1125738277413181	0.3967716113284860	0.4148686873600892
0.3775824868179063	0.6246617154917087	0.4912555318597171
0.1667485591041586	0.1007020765187988	0.5975038159465403
0.8002544326509243	0.4248402520104078	0.4819488194275616
0.8397993035080406	0.7974454838195821	0.4720280905686186
0.0900122389235265	0.7270902827570700	0.5368439740180292
0.2396022289881079	0.6328947465291196	0.5308085878259247
0.7178037315368465	0.1800867301405208	0.5705964123708006
0.7316760862951315	0.2645088359604158	0.6012609331254428
0.9102454614621850	0.8054696157462271	0.5444003176031538

0.3757287612686838	0.8543151336416149	0.5594702112882143
0.0255559729933952	0.6407899304248703	0.5443821358473152
0.1457319985399027	0.3137495872786050	0.5827582446431580
0.1324825212505323	0.9931802813509132	0.5306929842506972
0.6220241161712062	0.5554777767689821	0.6156586067620526
0.6059114922839836	0.6632855754969409	0.5414863942420994
0.2388416290923457	0.3385561534251936	0.4094753651121105
0.0628644286347102	0.9529861843457258	0.4637775450966105
0.6876326620899406	0.7425073463527438	0.5287031480480641
0.6919473328043529	0.5328572733499244	0.5468182084427091
0.4579369626607244	0.0592642444607200	0.4530605088604288
0.4697812767846740	0.1346457741152549	0.5007287984468790
0.0614334811776763	0.2670460081761140	0.5247469240847452
0.1111480845169378	0.6605691203262878	0.4359782265345094
0.8927055492914259	0.3890182574226921	0.3777124693981797
0.2465748269552473	0.0782270994409184	0.4107687717669637
0.3611559713175274	0.8584345574193509	0.4418672803664555
0.8896090249863968	0.0874035618699226	0.4596991797441978
0.8410296490097973	0.6027703802803418	0.4294498219817721
0.4848909810468939	0.4608840945964925	0.3758579191393238
0.6273820932447839	0.1025981982764303	0.4213136886898962
0.4577026705785330	0.6785910300670287	0.3861052263096446
0.6888112505937251	0.9317367305829904	0.4213278170851734
0.2320104156041154	0.6400844696958067	0.3891100430406694
0.4160063887200402	0.5840947880204800	0.3919203493669018
0.6824556729832822	0.9696908965361536	0.3492187864958934
0.9296138767264650	0.3335325837192675	0.4403034859650105
0.7014539534256866	0.6465105006584074	0.4437289051738535
0.0214197224641493	0.1144425925080726	0.4201323928046716
0.2004894203139563	0.1076657441924784	0.4821160802865773
0.7608435677614785	0.1207703533433370	0.3806432689926546
0.2571783410033420	0.8451646751190226	0.3811222642445816
0.3276118066135611	0.4560322796107151	0.3943471772987185
0.5599320903715657	0.2651260334798733	0.4435339013015867
0.3780988356665846	0.2534119583878712	0.3824039993307105
0.8404233010382203	0.2519446938316663	0.5107170201823480
0.3595796853686067	0.9584253244543368	0.3869365399366218
0.9224075460186408	0.2070149143788861	0.4532315959916962
0.4098637624263691	0.0348375601715654	0.3449151433000002
0.5973031344152133	0.2054512172201392	0.3855932915930153
0.2444424431459161	0.2084070182475593	0.4025546413189538
0.0359371545480152	0.5663293874809244	0.4194962053947695
0.4836108830093477	0.8038903353719772	0.3766498371623837
0.0338304087979951	0.8137289727246364	0.3724031817041620

0.7818512470497373	0.8168984652112333	0.3715915005505182
0.1279803285095942	0.7400219820402498	0.3672839552280374
0.8245209627888408	0.7295443784707586	0.3910120265299868
0.9589188353592484	0.4878760972846086	0.4386715633714233
0.9036350147112104	0.9513416761431406	0.3820929173019230
0.5716217621290048	0.7560048988023035	0.4271974531383584
0.0339599614633751	0.9956768131819730	0.3573129933182485
0.1743394083869253	0.1795837809803905	0.0730020524459717
0.6086650060737775	0.5367485455768254	0.0881265763274153
0.9345264895023004	0.2189244103927991	0.0734919107620873
0.7385727048491737	0.4161109088326144	0.0611686940176455
0.7266945481995861	0.7328943900905130	0.1017825170782035
0.1994495165023142	0.9445234935962232	0.0675131989049087
0.7116839397250544	0.1502391140630216	0.0681296399562700
0.3712089232906521	0.2449783392983488	0.0484183591876980
0.3516134780456117	0.6616618543349577	0.0323481735953237
0.9346191197816232	0.1239161130837946	0.0668159110387702
0.8655269252208178	0.4738603867998600	0.0608048996826580
0.7979352916933863	0.5853843782586198	0.0526502613528825
0.6211132581531871	0.7926296632940085	0.0701649471821868
0.2520974670825724	0.1300108010016605	0.0191679856307789
0.3357990933259871	0.6849843114921809	0.1087979912197330
0.2171212735100214	0.4415249041458279	0.0307603115267524
0.5189071769160732	0.2238567792942021	0.0691790245502869
0.5904762849014356	0.0873517743498503	0.0738551880355830
0.2671843032031989	0.8620666818067991	0.0678132529573305
0.1178512180134805	0.7071161312996787	0.0625044938769678
0.5562352261425515	0.4381756103719993	0.0137164182974060
0.9545459821722482	0.3457419328513798	0.0761917684423834
0.3972996119347551	0.0771321475585957	0.9649456433458612
0.1041562235897885	0.3246779419089382	0.0565042870671317
0.2968794509179862	0.0064823506211347	0.9606259119659574
0.8726199368917282	0.5718362716636245	0.9847254735622076
0.5347617390309105	0.3689794272319824	0.0672215307079370
0.9750354085138020	0.6726620484336163	0.0438348647970880
0.3230460937364523	0.3795296414055707	0.0564777722798244
0.6074701928315009	0.9781468167128998	0.0307840536097411
0.4527752531961846	0.0039210964248995	0.0390231087817697
0.0475245056663138	0.0447594691938409	0.0165381510915321
0.9788137090906918	0.0003488507088839	0.0773115378370848
0.4892088540809843	0.8857029012816182	0.0636644418455482
0.0654544120259713	0.5431208599869567	0.0401936326184256
0.4401957052534216	0.8186697305805939	0.0130180794745160
0.2121549150859768	0.5758647137850764	0.0546953391814330

0. 8157814809597159	0. 9432938574926396	0. 0039176254529143
0. 5304259294680349	0. 4589612890147077	0. 9012165025077672
0. 5396139358840627	0. 2661342099959363	0. 9091805922592298
0. 5568828821465929	0. 1313230364926517	0. 8924098494872087
0. 0066715091546584	0. 7699433853369927	0. 8765685169867872
0. 7056139235005433	0. 7329122442835795	0. 9906530041800224
0. 1397881110734306	0. 6746708102932011	0. 9104711777293940
0. 7327627076303649	0. 2579438461920178	0. 9427355663260624
0. 9741766059429716	0. 2431723766304614	0. 9297507338729896
0. 0039254726448615	0. 8755048996418597	0. 0543771432472482
0. 2094624101532398	0. 3879544327643645	0. 8099853071902702
0. 6410809314341046	0. 4197867839763104	0. 8225091880538012
0. 3069346880918339	0. 5132564751651243	0. 5311186246104808
0. 4376149606254538	0. 4057516776665952	0. 5269403185117708
0. 6009067686471489	0. 3895070822763140	0. 5250916905282692
0. 5738973506188680	0. 6062644798065007	0. 0400082425969539
0. 6349257665397017	0. 5163834871447177	0. 9343642783750088
0. 1146853552666201	0. 2284924339384858	0. 9689052119276306
0. 8601972243976119	0. 3985481635917931	0. 8754510677581310
0. 8145764873210721	0. 2226090440008140	0. 9423881787096454
0. 0739360721327249	0. 2544673358960480	0. 9293935528200320
0. 5849412862260842	0. 3030604576703456	0. 9389179549122232
0. 7355791287834351	0. 9644326561770464	0. 9787500025682736
0. 4999581103955240	0. 1400772562286608	0. 9326810371735688
0. 9093093074506527	0. 8942196399680791	0. 0580650994761889
0. 9400072560964314	0. 7968060142564312	0. 8459419088667297
0. 1267174084561000	0. 7312366185894499	0. 9228148496111808
0. 6789522767793014	0. 7158609764641489	0. 9445108538495550
0. 8077748456157203	0. 3914543253058577	0. 8312867240213637
0. 8017618616711455	0. 0795882970485064	0. 8964072137309298
0. 9542323084775922	0. 4060676253302763	0. 9365131044128686
0. 2255521137951443	0. 1310039334016514	0. 8528645579961391
0. 2913389731776182	0. 9428660725333120	0. 8955493060429425
0. 4254316705222621	0. 2801021092677387	0. 8068279981746905
0. 1615651176747213	0. 3921165413234605	0. 6310530771346078
0. 0533473948389963	0. 0462412151551979	0. 9366162309369720
0. 8715371661110142	0. 0345667141872621	0. 7740457986352194
0. 4631766967753911	0. 6335125284203244	0. 9780915201314284
0. 7694956158030920	0. 5745266341048229	0. 9127950911275832
0. 7505624739354767	0. 7189266896900828	0. 7760353471889571
0. 5998621663609658	0. 1581928650979167	0. 8094869974331028
0. 3720966060863513	0. 7998938748959016	0. 9334605837882568
0. 7060913405209004	0. 8539567018944081	0. 8882398654839372
0. 2638651342748574	0. 3486647210231166	0. 8905506861156361

0.4853665209345111	0.7200279044859635	0.8256577901115504
0.3785761996963757	0.5718538422052184	0.8197796600674925
0.0571991467906845	0.9320363855876488	0.8137250205837129
0.2991613644954842	0.0784828107759354	0.7333173908931404
0.2898864422918642	0.8193077054001071	0.7779438937381965
0.0889404198074415	0.2321523375010532	0.7724443073126195
0.6894467851683178	0.9217536815863060	0.7674955713468111
0.4338630418807476	0.9559485685985950	0.7780672725575642
0.8186917368329413	0.2452724872304965	0.7846717016410716
0.1615556107884074	0.5778602061056303	0.8945759953152198
0.5032713560806146	0.3698151911548681	0.6656505145083452
0.4993805111371564	0.7720702142299612	0.6776925962597041
0.0231267463947261	0.5189579185395152	0.7894714364213395
0.8106693962855309	0.8302040413477871	0.6832794386096716
0.5419996890041541	0.4312808134644692	0.8192334912122218
0.5600148840154229	0.5257745370625740	0.6892315826975115
0.3971714986162713	0.2187881433682991	0.6743021094827483
0.8560716930734423	0.0916464186265213	0.6486427181691372
0.3494685951674050	0.8901945323641387	0.6330025042398871
0.3653987931108443	0.6354401193118002	0.6639251369033502
0.9092372366850052	0.4391087237145921	0.6318210500783974
0.7623872777514630	0.5662001917078143	0.7761070497060142
0.6074539316788877	0.1199974531088858	0.6830428521930431
0.1042492067115213	0.6329695879276621	0.7058018305970313
0.0885387925215156	0.1633058242378077	0.6511722967180581
0.4611823299180691	0.0262150877728003	0.6083995711853455
0.1390645721992373	0.9593095976093980	0.6862970160733706
0.0774209160883709	0.7860684770465315	0.7009557955009840
0.7552277602795771	0.3176501879397860	0.6703948370678238
0.1585783474923013	0.3849263906116311	0.7673762873772195
0.2953498659234647	0.4551364286624993	0.5355728975844123
0.6515396375111303	0.9218928678540148	0.5040309041784836
0.2122204984452467	0.3943573917774347	0.4217578274095750
0.0656131569991755	0.8128186524316511	0.5701228022162607
0.8420219675591576	0.0308836103258082	0.5268568512125464
0.4227792114439821	0.8382884988819486	0.5170690758353746
0.9352696778896188	0.5935390536537964	0.6068381031961454
0.2145053814625021	0.0703667476335669	0.5615996399272059
0.3984326671835313	0.2198131503934817	0.5400219387845869
0.7491455242365967	0.4348870473392171	0.5231528578965824
0.0704203588410679	0.9510543033067368	0.5139724718464429
0.5102746657298752	0.3649988409443616	0.5337037318390827
0.5163893807400480	0.0853987386198252	0.4872632625204078
0.3329824433953272	0.6085290210271741	0.5341272211248125

0.1418422870180281	0.2648176365570485	0.5553122334045290
0.7158602732583991	0.2351807900241498	0.5584194388967618
0.6669438476070821	0.5783951033897323	0.5751309924692002
0.0943225853475298	0.6722919307486547	0.5194893828024338
0.8192415567343138	0.8035396484294238	0.5216915650549137
0.5979413944851878	0.7166548158857851	0.5214804904884334
0.9436789634370144	0.3881406257015594	0.4195407046405607
0.7333446165592634	0.9326620861125000	0.3764021342406881
0.7701988874482026	0.6383034662015135	0.4093505965405772
0.6611372607970218	0.1184717787328008	0.3763478572541907
0.9204794883781264	0.1217069110708136	0.4219906753859031
0.3951643419640247	0.6391692394758841	0.4061221920676776
0.1304994318831248	0.6446164345777158	0.3887490935707077
0.4075783828164687	0.4915419160236644	0.3909114676175064
0.3476785350801717	0.8706730193935026	0.3934065656339727
0.1867280921712476	0.1175748166671932	0.4332912824095232
0.3721163536256842	0.0187909196959439	0.3876956939364943
0.0044180132832218	0.9532235626967216	0.3862299127520691
0.9206064369508457	0.2582289171314769	0.4794241433081742
0.5490804970425344	0.2561929630616744	0.3955779966343489
0.2774406805166484	0.2561669010157933	0.3796423817608921
0.8533948303974178	0.7858638838324964	0.3930124940609948
0.9652835056400444	0.5440027447002393	0.4493095869024066
0.1286348001374792	0.7982085551744054	0.3658601418436609
0.5536242116278574	0.7620440465790970	0.3792377792578510
0.2254612257665883	0.1849209725883636	0.0316115297557203
0.1869346546427420	0.8906729461424648	0.0524634523234123
0.2847791140474037	0.6666690819262028	0.0701034924146483
0.2482005378249147	0.3860146066171934	0.0252103105647329
0.6404871288057686	0.5887626482433843	0.0745897389583709
0.7178050432022424	0.7729465080537324	0.0673819690114510
0.4603874517927415	0.2697653849461137	0.0579892941415700
0.6136369416726294	0.1407036043663954	0.0584123462973983
0.8763229341894295	0.1724331169037933	0.0633114969615320
0.8404246860802496	0.4183340919196483	0.0569446954217548
0.1622998463206308	0.5334229324881070	0.0310452182318138
0.4665572585225593	0.8286573369777507	0.0604596330322317
0.5330358581769874	0.9842032504019740	0.0646722131051118
0.5458508554285866	0.4898452676963948	0.9430333757395092
0.3307935716080319	0.0469065846912219	0.9930377160534184
0.0215697468810228	0.3025117037321258	0.0768914614790953
0.5731767821730518	0.4217002123003293	0.0613882582690645
0.0274275541784570	0.7220927470550612	0.0454576371990562
0.8921993772525686	0.5782224497730393	0.0333910152117185

0.0331975922801821 0.0469006912108160 0.0667658003581988

Initial state of C2H2 migration on PCC model
1.00000000000000
9.8709001540999992 0.0000000000000000 0.0000000000000000
0.0000000000000000 20.0000000000000000 0.0000000000000000
0.0000000000000000 0.0000000000000000 20.0000000000000000
C H O
98 242 120
Direct
0.4576364476172661 0.4528326956263941 0.5451625138484194
0.9575310889974702 0.4533719981451103 0.5433535735240848
0.2075650784640806 0.4533159547047014 0.5438485998879308
0.7074656745205375 0.4533250730348640 0.5434618404158487
0.8325152299518195 0.4876227676569832 0.5514968035359979
0.0825300219733771 0.4876146409805233 0.5519117097094288
0.3327216969036807 0.4874188039564479 0.5527627749869247
0.5825126657514107 0.4874014541103498 0.5528524783523372
0.2076100823334880 0.3906731442503284 0.5097243743647336
0.7075299246754156 0.3902688097500275 0.5099984912058090
0.4576482761496116 0.3898831885935033 0.5110486726652222
0.9575906305622690 0.3903981656677951 0.5097069758442625
0.0826580960575720 0.5590221848524124 0.5497711833020337
0.8326811073620244 0.5589742859697683 0.5495730078488957
0.5826452819874686 0.5589720473056249 0.5506635229081095
0.3328546217250187 0.5588788343840043 0.5499998908611946
0.3327939058722379 0.3643933426665467 0.4865972957560008
0.5824009628688729 0.3634605956537744 0.4873815851667332
0.0827334324345660 0.3640927960204755 0.4865206341353255
0.8325123277478451 0.3638308521385011 0.4866276189258383
0.9578348721235156 0.5928568002154873 0.5397748513782251
0.4579174337322359 0.5929390331509313 0.5405061448192265
0.7075297963463509 0.5929759142157986 0.5403642391744012
0.2077667921363237 0.5928252557488631 0.5399359986236064
0.5827217781005137 0.3187958283962547 0.4316698290842883
0.0827431226587223 0.3188171312538249 0.4314753346269625
0.8326191313943845 0.3186112322337953 0.4315022790351103
0.3328649643190647 0.3193873754386539 0.4313168197598085
0.9580288250382546 0.6553943492970606 0.5054639158554152
0.4580420412090985 0.6556943017991649 0.5060243530740508
0.7076128766820332 0.6556429213561175 0.5060122232631198
0.2077728329852171 0.6555276108294557 0.5055526763847951
0.7076176988438604 0.2998455182645418 0.4016999133157665
0.2078071483400915 0.3000728894949814 0.4017962384446502

0.4578871279051031	0.2998341873528778	0.4019242971629277
0.9577225438978700	0.2992409939380627	0.4021592070435804
0.5829300121896049	0.6833997996767818	0.4838983130672392
0.0828001179550789	0.6827823582641815	0.4830712062185356
0.3329657241910791	0.6833165070488016	0.4836805821844033
0.8327754058247802	0.6827580729386047	0.4833275236240693
0.4580328277911921	0.2618802155172769	0.3414706666993462
0.9576994802453652	0.2613480608633115	0.3415645693977902
0.7078031173549707	0.2623707532149713	0.3409432884626486
0.2079228629861699	0.2616260807781766	0.3417786753988154
0.0829391958298069	0.7317720585566491	0.4313132697372108
0.5827847627931373	0.7331585290716096	0.4326315336881333
0.3329581204292474	0.7331225951664654	0.4325084821765967
0.8327555692233991	0.7318739612327249	0.4313555860537468
0.3329154670773915	0.2416865101447359	0.3127498275298811
0.5829479127285672	0.2422027361065935	0.3120113352894969
0.8326636116641098	0.2422500962606740	0.3117156858039233
0.0831530184906386	0.2412892717308629	0.3128047815015151
0.4579206101739370	0.7555343946279920	0.4053386741610942
0.9579643272574824	0.7536938764515692	0.4036829004933512
0.2080183267573327	0.7542756967803594	0.4043300425857700
0.7075057895524643	0.7545696168917776	0.4044361620814886
0.5830767541785320	0.1943657990709951	0.2590143501648950
0.8327691618609541	0.1946080607626960	0.2584693693978395
0.3329326749689424	0.1934474255620813	0.2601012267626694
0.0829789480051813	0.1936911594115817	0.2596932722184961
0.7077693135524282	0.1666379792545864	0.2369040015639831
0.2079563023894566	0.1655401838323473	0.2383945352345436
0.4580828283785664	0.1656269496704704	0.2385335434641609
0.9578342672815172	0.1661843665883802	0.2376048396015087
0.4579326675537242	0.7985153390338432	0.3484097514317506
0.9576361143863223	0.7976738265110179	0.3476108460983522
0.7076492371042136	0.7980248255539809	0.3478799128522914
0.2080290632026677	0.7977892505554102	0.3478533032379333
0.3329842558358778	0.8200472270650420	0.3204225009254268
0.5827918778126718	0.8203022202499008	0.3205104143828723
0.8326935027684200	0.8201995383153909	0.3204832222925257
0.0828022720934779	0.8201264269972316	0.3205078049159196
0.2078602165487157	0.1008909367050099	0.2080036092503630
0.7076700605733738	0.1012663862059438	0.2079227081801381
0.9577520870320972	0.1011844140570312	0.2077746036885290
0.4580119263279187	0.1006573727532627	0.2088863614309746
0.5830210899958749	0.0662049522086654	0.2019435410548751
0.3330079818314906	0.0662119716580959	0.2015522066556339

0.0826539753001743	0.0664414079116875	0.2009804631931803
0.8325416869291768	0.0665418139372120	0.2012903284725667
0.3327510000285042	0.8698036983307392	0.2691575718272923
0.5829651682900379	0.8697450907617462	0.2689856173608824
0.0825327404977104	0.8706796292361982	0.2700756768905935
0.8326902469595920	0.8704961359665573	0.2699282337102009
0.7079093809856686	0.8976274225538776	0.2473514996304365
0.2076151021158569	0.8978321426024720	0.2477127195872681
0.4579161242784389	0.8976322010454121	0.2474496400234864
0.9575741871709684	0.8986699625191932	0.2488113750838458
0.5829056322462869	0.9949347783328064	0.2049180672352748
0.3328293645188806	0.9948712328625512	0.2048961259087846
0.0826321160717433	0.9950719760281932	0.2048054861213895
0.8325002358536706	0.9951584533494298	0.2048598928474925
0.2076482663560965	0.9609927807373136	0.2143557814999838
0.7078046965634825	0.9608767030263800	0.2141410514446118
0.4578857863306480	0.9609786316482096	0.2145021673710769
0.9575756701347216	0.9614090874633632	0.2149938589539240
0.4498973301674982	0.4893239196475291	0.3910214559117874
0.5714975885101898	0.4959259251165629	0.3947281187334075
0.3411863893082831	0.4826504005451051	0.3875819399436213
0.6796081570380836	0.5005837465652831	0.3962470119712153
0.7596981391280883	0.7499995775338565	0.0196889434100516
0.4827241886968231	0.4502333990749493	0.0870581469067776
0.8996124008710616	0.7245536175212495	0.9861993796472678
0.5378944119244000	0.4437728114708424	0.0125825373221096
0.4527485108718418	0.9602619982864954	0.8924595042376925
0.0765666166612678	0.9201264822408960	0.9026727662744056
0.7808119770997122	0.8071013355789781	0.9367907331878820
0.3986376081756126	0.7621805972420653	0.9770817964642724
0.3792538647577828	0.2361200241437233	0.9243123941163156
0.5785530988588223	0.2479488699309756	0.9534832453180044
0.6251740522591568	0.3158248960166913	0.1234846215041142
0.2347284305934273	0.8361612589614489	0.9982632846606198
0.0608772700177791	0.6592603946256691	0.9942720449412554
0.0942806950900966	0.7219135657778917	0.9441252798989886
0.3771137129496349	0.8682919007078922	0.9995331833205749
0.3300172235361092	0.1621157863959386	0.9196975549400264
0.5753791630328096	0.3358547442935826	0.8275462905809701
0.8555006624741144	0.3662140603198210	0.9361835548939280
0.1371103776353647	0.4270981030581545	0.0051742925290000
0.6968389598682612	0.7048401943427471	0.9048896668847840
0.1664048179577061	0.9939154830069792	0.6637092769361904
0.1946761952582438	0.9958851080708072	0.9922074293004938

0.8719277717559031	0.8670461866743419	0.9121408727968972
0.5484785277376079	0.0228794183003701	0.8837691428083921
0.0504253986630250	0.8725117351165186	0.9644775868822002
0.3232856297160488	0.8751233097496069	0.8982880258833905
0.3983353616255835	0.8608766919612346	0.8314182675983628
0.2178447045203451	0.0893283286761322	0.8013875079771988
0.0002395516623379	0.9550876504994362	0.7839814118340639
0.8433871206603150	0.9712947821212424	0.7687392695897268
0.9623352582782010	0.2781032610481468	0.9224818229166796
0.3170043564135222	0.0335715422928005	0.7787667881807354
0.8905866902696791	0.2767759041258795	0.9960320384126816
0.8765009063594288	0.6387064810569455	0.8574743989965077
0.1675622366899888	0.2504354266465038	0.8797052692784574
0.4920291729778303	0.4012880783083874	0.8496228897643529
0.9856797371405048	0.5464261940496814	0.8649410003462074
0.9345705995127644	0.4308532341815203	0.9609148348614313
0.2886461197342355	0.9741940723740182	0.0531162354019031
0.0219348846901914	0.0299280825798024	0.6471594305886574
0.6077620035884241	0.6365417076698051	0.8330031342897228
0.6120107238840066	0.6141063629811309	0.7570705782810877
0.1314780811191704	0.4387849866882945	0.8572184429755141
0.2287653333718265	0.3948334080958473	0.9021382009212512
0.1480575305218223	0.5454438632614320	0.8733165477358462
0.8906181740222539	0.0227464224033283	0.8628228556669867
0.8393332836965683	0.0886513482898656	0.9008681489264034
0.5088195650086352	0.3140033624384885	0.9243063949764284
0.7456090679218205	0.8318525378381237	0.8394353648888634
0.2040762307000578	0.9029329268850240	0.8043619804480548
0.7777455111115744	0.2152684627691544	0.9247396216961628
0.7767219512719715	0.5810406804772476	0.8480067311635603
0.1186101468692803	0.3245975045075307	0.8600428093917488
0.3082616369332271	0.6542983540941827	0.7866225812146617
0.3651881437188645	0.2813710834845246	0.7552725232228475
0.1016164058004249	0.8029087817115769	0.8767074303910904
0.2474799725073238	0.7105884276897509	0.8370470200191910
0.3621179225666145	0.2262940976875631	0.8136163851230936
0.0362412296268959	0.7391604804473326	0.8404639390858589
0.8818844928466001	0.4210831742184505	0.8424870445535813
0.9491415561113388	0.4085054025106146	0.7684557579565686
0.6799795497045854	0.0010904244808514	0.7124678311545257
0.9748998969440767	0.9264844209116544	0.6426502386575906
0.8207940643298782	0.0963534627811822	0.6677207217844905
0.1678137498194835	0.1400557884231502	0.7012306070625710
0.7745235972001995	0.3450527583349608	0.7896127094677221

0.1369685991295641	0.8712996878929915	0.7389607397073440
0.7571472751408134	0.8508963146051625	0.7623829155154784
0.4708239249632093	0.1038434928633480	0.8309294866556459
0.8399611578114837	0.7466990327706734	0.7979496231833288
0.9971754140095626	0.2467294517602206	0.8004553188553628
0.6056399246735121	0.1349037670009980	0.8658043398752592
0.9080576573493788	0.6842421975547066	0.7601115077480040
0.9465461000407296	0.2463714442401388	0.7237872913099126
0.7742934753309437	0.2663978438990920	0.7866230314859715
0.0599330472838805	0.5302474797645933	0.7692820892324486
0.3655628405818451	0.3962305757500327	0.6979557779410918
0.5248960507084385	0.4741065820900719	0.7076472763031995
0.5905848687318288	0.0950623236783870	0.6846552305657679
0.8230444826029558	0.9187162507970620	0.6684026715112348
0.6110221102067489	0.9272971413609300	0.7094934465288821
0.2899296488050968	0.1913129372496620	0.7181665880782178
0.9176195048650000	0.1271312774389403	0.6094328794654151
0.3857386196456533	0.0297192270483050	0.6770715296547877
0.4439867725335300	0.0804045540946507	0.5924268750714358
0.3735738875828403	0.9525066238283100	0.6959781841826608
0.7930728031687976	0.0440431786271259	0.5755641036548155
0.3652026080264953	0.1264422889725501	0.6501092454163904
0.6450736419668744	0.0281746297202711	0.5412260235092131
0.5618510242834793	0.7939024863549932	0.7977771749153367
0.8419374166812766	0.3030065565035289	0.6459202967472659
0.6711518309281920	0.3111789184912573	0.7015473466470105
0.3171067496181711	0.6649239652185622	0.6735218982309096
0.0552802802229285	0.4893682267107428	0.7014614811315331
0.3199523211819754	0.3332022227411358	0.6528454515787473
0.4189422233320376	0.4763330995843002	0.7651735568598591
0.6412886493817134	0.0949399575054394	0.7599248302644003
0.1085339820787999	0.0187046785579283	0.5572497941760324
0.4281666578905781	0.9850890996806352	0.5265266385307084
0.4466036292525409	0.0537264524758286	0.4862759914283376
0.1624418416839166	0.0800410340478823	0.5141301842737288
0.8779640402352750	0.1876560262570356	0.5236502009128990
0.8465428180499800	0.8550062704851568	0.5893973340556155
0.2522837031744918	0.2021390944242825	0.5236592956629961
0.3139113282162934	0.7043400792082665	0.6052103620167186
0.6311671593885463	0.2889716099122425	0.6269638360645101
0.9571783534983024	0.2592941077014657	0.6112095482305531
0.4223356283812181	0.7516490039818694	0.8011282158975080
0.6353011488941777	0.7223890079181298	0.7166910941113249
0.5219340079651404	0.6919874776299064	0.6727178953933144

0.6851258424824419	0.4163289158247075	0.6737735559363710
0.3561121941511135	0.5599684919104060	0.7124267918514579
0.7452438055372633	0.4515811145097957	0.7369733463928344
0.0171601664562272	0.3638589642241343	0.6752385096311022
0.1553562734352592	0.3833752468862877	0.7078731263107232
0.2010219746279028	0.5766454453490664	0.7133838379232272
0.1068945464843382	0.8698169295962168	0.5114853826080359
0.7833152224897539	0.8853112978879458	0.4839887624764025
0.7336604753396467	0.1151660081178515	0.5087745064251818
0.8512279500429616	0.7871485645736136	0.5455668069001377
0.2509822168923170	0.1544447106246426	0.4574576909592334
0.0396003265756042	0.1836201392819157	0.5275744576624118
0.9468619247747984	0.5985162031951430	0.6972968474529126
0.8085543131854005	0.6357168738092542	0.6798046171832043
0.3982060304182565	0.2498669397485671	0.5810283597162742
0.2666216277817842	0.2272964030131405	0.6204396129514765
0.2997825247697118	0.8857795821707529	0.5281301985489705
0.0170497749904613	0.0625014313242627	0.4330464930197067
0.7252683260438606	0.1487310068306172	0.4403106496485194
0.1406023602635521	0.8420390910628516	0.5874094295061806
0.7794524414065017	0.9639418094797008	0.4854470059621863
0.2198476204197139	0.7736665683881397	0.6733113623672231
0.4555489591022496	0.8467748105384980	0.7237453564793851
0.0631443512022918	0.7846282682867246	0.6809693665453659
0.6199649934856195	0.2051549149199008	0.5413575359126717
0.8337470056664945	0.0954406886763529	0.3711959052971247
0.6859627301449625	0.0639409367551671	0.3720973011092511
0.4912147276645756	0.8594169998579170	0.6472022230000012
0.5411505049962559	0.1807374004855187	0.6052684358258129
0.0933714090716065	0.0917557101015861	0.3725162729417603
0.4582559697786522	0.8758320573818145	0.5266651480058063
0.9634771754303548	0.9228680596296928	0.4391821021211923
0.3527858254395608	0.9376877226300938	0.4375273554699838
0.2358756015195381	0.9444018411628480	0.3855523744336491
0.0874026081548617	0.9649316198255180	0.4647067782022843
0.5118284871163877	0.7711104827425310	0.5776612177780657
0.6433239882037725	0.8168710701256228	0.5687452346339037
0.3088813176155084	0.0441255629168797	0.3938592889238241
0.3635298557627763	0.1078772431757932	0.3561564772219555
0.6590501675355729	0.9567762553782624	0.3988854477745076
0.5121463126355766	0.9737889235076702	0.3708238183551507
0.0982567005683617	0.4480075079566072	0.3324530127034989
0.0296312436322354	0.5778984814334894	0.2549238945772288
0.9932549751875708	0.7765430028601294	0.1508709478420621

0.1309948618392892	0.6284520361560975	0.2865139502646629
0.3010725505614543	0.4733637452486247	0.2108446134213760
0.8526118113292165	0.8024043520171942	0.1186059846232916
0.3437388622994611	0.5893201415641558	0.1871954962692739
0.1293039288595184	0.5246308139003968	0.3365018536744572
0.7433428237136458	0.6741146465783269	0.1684337503802037
0.5749822472199667	0.6303342975309988	0.3255699685173316
0.7738529150411544	0.5326957671047617	0.0615695036319265
0.4409825148765887	0.6162720829682340	0.2484213874771617
0.7882583627049009	0.6543973780736965	0.2443143066617669
0.8097621134021629	0.6344079575626392	0.3506529490515782
0.0147910680000212	0.4006249954293916	0.2247100921378364
0.1836644458747983	0.7156436397473376	0.0519931512166274
0.3608457517757572	0.7666811350004010	0.1091827302862244
0.8309098159883407	0.2886687439132567	0.1086415093614243
0.2738771362128407	0.4541259839499888	0.2856943692327822
0.7447356347136070	0.6232796743244233	0.9871890888456047
0.5361829958116333	0.6498711360481280	0.1457393761359744
0.6341521394216366	0.6380748999031740	0.0828005332575779
0.0961474181808353	0.7425371407122084	0.2386743194276861
0.6420079474168073	0.5037005267819517	0.1373372842696271
0.8812524413678934	0.4669770955457196	0.2890729440507803
0.9800764420408050	0.6986711229295738	0.2052967956441258
0.7243479352405700	0.4639111412936891	0.2831688043392944
0.5154652125756524	0.5401604337571541	0.1748305229952125
0.6103105875435242	0.7883767837205203	0.1017588386880292
0.0718646339471462	0.1507055703798763	0.9370849608301088
0.0031834994071244	0.1484031837340537	0.7665437473039979
0.3085374041164357	0.0818575709570411	0.0035183985411094
0.9650228322439550	0.3621401232274322	0.2893005745533928
0.8827408082933246	0.2159892432521043	0.0875129600808437
0.5280013458409220	0.9308878646523484	0.0479589583429127
0.1897730463669977	0.7029319677717731	0.1300673484936618
0.4291081939352324	0.7965596563414299	0.1734192997974869
0.3015478395977829	0.3702000394863377	0.2061842965741687
0.6669776718818008	0.5529519704527703	0.9833245750781000
0.7717020554849595	0.5697703583817253	0.3086122945936585
0.7911368303120925	0.4544564269795432	0.0753930872503361
0.4590072969001757	0.5848731530005324	0.3488817785086816
0.7119959942974924	0.9031309570556160	0.9828592958622444
0.1233073376733333	0.1884328778569613	0.0015780531385464
0.9255939885647544	0.1431907607248532	0.0206797474459201
0.4255250980380629	0.0552690043961517	0.9562335589058282
0.6958223392449824	0.8465882229557729	0.0659334639139690

0.1243071011028574	0.9112372293663238	0.0539973233702284
0.0362489916001680	0.8446907304280834	0.0757123961828195
0.4311662503141053	0.8844769865178672	0.0916852837905921
0.7760120888931331	0.9486533743682916	0.0395096412825875
0.6462512224946885	0.1488543986866006	0.0636455041741114
0.3052410873319543	0.5822261820198226	0.9558579098088076
0.1358598059421666	0.3666674028327080	0.1488225068746720
0.0133850514431872	0.5874374768518307	0.0753281964817429
0.4805043357977527	0.2957059154774493	0.0496818319456463
0.5450365480003464	0.3942992664526996	0.2724933230531592
0.9106193809306397	0.5290885052602965	0.1503838309563574
0.7223446504499935	0.4554185135037354	0.9425994521743568
0.4988148952048426	0.1331641496768718	0.0347521273386239
0.3447048049568528	0.3965279054018592	0.0118008414363116
0.2373464285010986	0.3353991677373012	0.0042312286153118
0.9116732038782850	0.6039450503172480	0.1764607477060085
0.0388563468208509	0.4338966802259892	0.0660366932875398
0.2703106932430915	0.5412099925373204	0.1065553965655920
0.5794502427829058	0.4839180826082792	0.9131087435867370
0.5302147399369485	0.5338423965045264	0.8281854916075755
0.4001942218817176	0.5206882956696038	0.8725599519320166
0.9030421454619814	0.0171278055989197	0.9881532032633328
0.0003463162265175	0.0008505007910232	0.0490932822472535
0.2182940591279014	0.0202045644284796	0.8766469374169191
0.0724246357142405	0.0297851155256346	0.9088471820792822
0.4999884149664754	0.7180446080466977	0.0194493196853720
0.0886199483273035	0.2662934251688672	0.0685760766724832
0.3488438816486292	0.3193383425942122	0.1481021817655073
0.9828428668931972	0.3491520693351793	0.1336713878437366
0.3149973839450261	0.6197202224529226	0.8845591375765522
0.5004072114579147	0.2319984226276033	0.0924889204562281
0.0472263395862621	0.5368361182618523	0.0184460591692847
0.6962181774295446	0.3535719997245574	0.1828588155792009
0.7371242809039639	0.1544949533310253	0.9686567433167718
0.5411186741863219	0.7063901105444205	0.9117279970771872
0.3187998392812122	0.6323882084732732	0.0559342232611612
0.4466858563477624	0.5899406677500291	0.0359173562513859
0.0037438596406305	0.0670945420375918	0.7666218956893598
0.8461805493621027	0.0889839425149467	0.0590855607908680
0.2436957477686820	0.2483705050251610	0.0766637433340226
0.5818214904839139	0.4412462030056284	0.2129396908542695
0.1403581653120255	0.5441062274602586	0.1546312790205563
0.4759829215933956	0.4229232279983349	0.0459892929848499
0.7997373887322650	0.7303082918270938	0.9781242738085654

0.4467616108295868	0.7179601270410939	0.9783220080871252
0.1741749233085310	0.2591617878840149	0.0435883202462721
0.3170750718394701	0.2062943186058043	0.8978292960026664
0.5041688357829893	0.2794981425527205	0.9616100019185562
0.3222306172620015	0.8352788937680790	0.9730763176844101
0.0663544194201448	0.7093094751268085	0.9907690999493464
0.7152194246041017	0.3333653787802804	0.1386074751579458
0.7739808498172372	0.8520059866669181	0.9149110713352684
0.5086405605688218	0.9930345859080388	0.9178448743460832
0.0333793208372429	0.8769808975786273	0.9148928862230050
0.0708808232335721	0.5223529537239504	0.8506778352998807
0.6145957554631029	0.7008745116948747	0.8773451465875612
0.8625092597376174	0.4155992182037225	0.9289162646274024
0.4995500433417068	0.3527300826348185	0.8554732416681131
0.2998516726482220	0.0647854032075883	0.8169486205314752
0.8733395528992425	0.2821572207812613	0.9472137211086102
0.9255810699183176	0.9896929124934328	0.7897757742114361
0.3484702511896010	0.8973222027199905	0.8551556953128300
0.0832732464110349	0.9921290127084350	0.6353686910995224
0.2002438645805802	0.9955574002619594	0.0422581993066202
0.3482665043613811	0.5872930992084429	0.0397852290416751
0.6172618161303117	0.5955836535731345	0.8026468718687001
0.1861801309165268	0.3972022863673377	0.8572453691261503
0.0855704345062673	0.2767702660394948	0.8673921217594813
0.8536850225511743	0.5943133820625216	0.8768009816813849
0.8915759962487819	0.0474140950681689	0.9067931195990016
0.3296321413693782	0.6834161916491237	0.8255780385641748
0.1165258069230142	0.7544506512547623	0.8678149982339391
0.3813215739298602	0.2336661908727665	0.7655335245833190
0.8627880878329245	0.4145343667643144	0.7940124624134142
0.5706673831292189	0.1003541223881848	0.8348741346586956
0.1215609416614564	0.9032949151143304	0.7758014610024310
0.7188781605906283	0.1743299832340083	0.9242991990946168
0.7340681339655836	0.8138178180620155	0.7919209290938041
0.9050708206563732	0.7090711756106186	0.8042017122538065
0.9349335416428838	0.2236518067342073	0.7682590151445079
0.7121559440000932	0.3053328713495571	0.7849543805202728
0.8888955423748737	0.0852462041052396	0.6328692791281858
0.6990476342490932	0.9516400928804832	0.7154988631363619
0.8997998306610278	0.8932926361852135	0.6479492736870593
0.2427255755182239	0.1657392318352311	0.6802600590515863
0.3170128180165159	0.3524550539374559	0.6981787986113889
0.0565795125988306	0.5359112769664800	0.7190036292379722
0.4239699122055078	0.4777465143200559	0.7156967823942493

0.3280955092518796	0.9969952299501076	0.7007216513923517
0.7441617855977232	0.0312633931829045	0.5335474771278476
0.4546397134798924	0.1052634880107437	0.6357492940019075
0.6706400729370368	0.0840103636964142	0.7130662283387252
0.4632809724714111	0.7955399472063153	0.7880566407737198
0.6782556634831050	0.3241441186462068	0.6533649980234129
0.3416315765912898	0.7093856981487443	0.6518113868725995
0.9400392150008154	0.2898158021468321	0.6496774764999769
0.1184924560947917	0.0357542356059637	0.5104811514395438
0.4675159482091016	0.0318491454863452	0.5286377694117204
0.0598014402575082	0.3994228441933727	0.7030839078546888
0.6145298914099371	0.6831345431840775	0.6896023984591368
0.6871783619577752	0.4596460609865946	0.6973742551919558
0.2927718438557921	0.5986567878109583	0.7173097092999354
0.2052633351375493	0.1648495261158117	0.4999955324374609
0.7996913486972637	0.8282010323285209	0.5514552008200581
0.9572139084910746	0.1925496942320478	0.5545598380323935
0.2994830509774399	0.2589344283551420	0.5856295881057491
0.9038223441521956	0.6433177582014065	0.6906455661517045
0.7273928756322203	0.1611926299669407	0.4897762127565978
0.1700836293690495	0.8435937437038366	0.5403620280522998
0.7853568697713771	0.9249447308151312	0.4541638031522220
0.1480941364023568	0.8085281979750663	0.6744816595138979
0.5660280620197607	0.2218011003536294	0.5803746809678598
0.7361351611434624	0.1071132738400660	0.3720891947305448
0.4700371085437834	0.8830432185592347	0.6898876095832503
0.0036451784336937	0.0759158722592449	0.3861860859869725
0.3879034731052241	0.9086192369570476	0.5133061927772783
0.0636141562047042	0.9210621790422761	0.4446106557999830
0.5422429259202218	0.8173799073688153	0.5778824754516702
0.3275560877393099	0.9609333888277362	0.3947737491292816
0.3001858336177662	0.0941076021694827	0.3907657561711401
0.6068520426717330	0.9884150086810806	0.3707650732933656
0.4312838045370606	0.6116037268225485	0.1981787043360172
0.0642715951021325	0.5945569477816289	0.2982341030571905
0.1609534645213498	0.4803792210542724	0.3534412940803811
0.8236249811047857	0.6694711159122201	0.1996243071484441
0.7893782069205409	0.4981239328214193	0.0969356510545489
0.7477085532463628	0.6190140478998349	0.3161236877511069
0.4742054154356414	0.6273825822878063	0.3253751342552459
0.3299156228730619	0.4420126491464021	0.2464687824008794
0.9483651934009716	0.8144998185138962	0.1288232211347708
0.0767981465312826	0.7135667874616178	0.2005200571242433
0.7989868917579229	0.4936605667374183	0.2992036200177965

0.5496269210826725 0.4968984985993741 0.1563747007332379
0.6104171466378685 0.6702820908279180 0.1185171971220476
0.9965596034585880 0.4057704526894856 0.2739471070885631
0.9068871625964834 0.2639299183299654 0.0844411257856108
0.2478760142352008 0.7062869050085666 0.0899692056109850
0.4297898635658895 0.8002350366146422 0.1249299654625680
0.6891238426372792 0.5923422649565755 0.0136143297309048
0.8385006455950474 0.1369256641919221 0.0468175999235850
0.2703879804678300 0.3336332091735950 0.1755021370685185
0.7000256093820896 0.7990847950061014 0.0825887913944526
0.0739034634934498 0.1479153176228787 0.9855949567998362
0.0622266126492317 0.1073899798958429 0.7643229443624979
0.3871269086389300 0.0970268557738811 0.9776599936377348
0.9723497000476512 0.5639576597000732 0.1685961567614555
0.6223569036591698 0.4078305272714926 0.2453483012185465
0.6373657814536369 0.4810567277061548 0.9543344528874924
0.0995342264006473 0.8653275542609148 0.0406018686710977
0.4311137989266015 0.9214808690591396 0.0586386149392038
0.5494962004257389 0.1563366685139079 0.0710953844575872
0.6972445402583641 0.9191205480691326 0.0294479471270113
0.2618579896529398 0.3799143867347230 0.9864328146918716
0.4760662390518206 0.4961814179613986 0.8496114704357633
0.2374785144572211 0.5318888187650740 0.1525432238183044
0.0513854978711412 0.4510548505484880 0.0197795772643846
0.2927619662011510 0.5762298618118705 0.9066471023655984
0.0327438355853413 0.5853372496739978 0.0273255623297904
0.4759829321445334 0.2807190497982556 0.0969800068906160
0.0412471884620834 0.3874289118532189 0.1431732409178568
0.9044927048394120 0.0054094277847880 0.0366389451540754
0.1533517848744895 0.0007406052295950 0.9101825303165382

Final state of C2H2 migration on PCC model
1.00000000000000
9.8709001540999992 0.0000000000000000 0.0000000000000000
0.0000000000000000 20.0000000000000000 0.0000000000000000
0.0000000000000000 0.0000000000000000 20.0000000000000000
C H O
98 242 120
Direct
0.4578439743632412 0.4544323402948835 0.5398968150645184
0.9576219790218310 0.4535983608542258 0.5414132334767329
0.2076957494153609 0.4543953247067851 0.5400306406481596
0.7078520048129033 0.4538950308893158 0.5406959337378549
0.8327457554641867 0.4879313588755166 0.5503326082128500

0.0824188673149923	0.4882358271701118	0.5494987097998509
0.3328057909538337	0.4885559229913329	0.5489686861417098
0.5829009883731020	0.4883529449064708	0.5489501139319545
0.2077617747116576	0.3923749206907502	0.5046148612860533
0.7078859068061018	0.3912344134160930	0.5062215030041650
0.4577791192885011	0.3923522967595401	0.5046202984007370
0.9577528987491865	0.3908794354743750	0.5067587670294839
0.0825262184868484	0.5597618178369465	0.5479927760274880
0.8326933389828464	0.5595253688260603	0.5485254291223786
0.5827733135510678	0.5598017225334130	0.5477769447549146
0.3327682369144285	0.5599720908269392	0.5476475211400047
0.3328364288561321	0.3667051460390086	0.4804964901283399
0.5829248664759422	0.3656528417406113	0.4817520992393230
0.0826475054350106	0.3654467528668947	0.4820920491297530
0.8329089030840452	0.3643487286221781	0.4831987423656264
0.9575377366047604	0.5936442418854256	0.5384408592027149
0.4578993392614770	0.5938378071586451	0.5379189386380205
0.7078880140193924	0.5936409442795572	0.5382996137746933
0.2075093657595130	0.5937938800183865	0.5379535008841210
0.5829219628454382	0.3207305850052286	0.4262872394269159
0.0826910177614225	0.3204355037967676	0.4266556862640518
0.8328267752869905	0.3197968971773563	0.4272653790011527
0.3328161515406172	0.3214495506549058	0.4255412563830422
0.9577106322053895	0.6558802035417988	0.5032229809181843
0.4578827374996730	0.6565139524529103	0.5034781662738332
0.7076742759391395	0.6560303453979572	0.5034056398590256
0.2074947136352567	0.6564324306614331	0.5034318397391376
0.7079808856365208	0.3012085690974816	0.3969760976697617
0.2075841360133322	0.3017039253612052	0.3966182933604396
0.4579876296019677	0.3017964392941084	0.3964456850409827
0.9577031199424064	0.3007711416788839	0.3974178406097028
0.5828927234244863	0.6836807279915327	0.4810515969052476
0.0824023506127722	0.6833076366619163	0.4806276652892805
0.3326668894331164	0.6839381287078945	0.4811279321268032
0.8326954110227848	0.6828275428041592	0.4803195290840159
0.4579674304617416	0.2631343421577532	0.3364755559690453
0.9575924649414856	0.2628530115947050	0.3369007933017094
0.7078466499610424	0.2633591342090610	0.3364365020598502
0.2075771914377696	0.2629652808466639	0.3367563219614761
0.0826316491143674	0.7318222500789605	0.4283211616631677
0.5827379907584774	0.7328487230439287	0.4292227263173859
0.3326531931819191	0.7332029592572032	0.4294715017291226
0.8326623956599182	0.7313379900877810	0.4278159455349636
0.3326598236057753	0.2427064807715460	0.3079084953966660

0.5829893258241309	0.2431016930733351	0.3074225205993291
0.8326679871080201	0.2431929626446602	0.3074268116992169
0.0825763765511909	0.2425814478073381	0.3080260627034309
0.4576514879842928	0.7551750912496852	0.4020053152193969
0.9577287186271296	0.7533036778380974	0.4002449736144535
0.2077051989289774	0.7542441272439715	0.4012656471976315
0.7074862483838538	0.7539762480009774	0.4008339657717315
0.5827706471609293	0.1949925259549175	0.2546716554769494
0.8324407061423131	0.1951566318672222	0.2545144705021312
0.3325298047070235	0.1943057783722384	0.2554442821817432
0.0824302552054134	0.1944188985636906	0.2553587376849942
0.7075436997192348	0.1670464836632472	0.2329876028928763
0.2073957902849405	0.1662604462806652	0.2340778152140627
0.4576734367124356	0.1663913630298075	0.2339643783423335
0.9573183884386326	0.1665818315488267	0.2337210268534763
0.4577071863272678	0.7983492348630278	0.3452403834648270
0.9574192812129140	0.7973341496240449	0.3441574187224415
0.7075148017674007	0.7976397270833377	0.3444575495421779
0.2076498460425077	0.7977055439062229	0.3446841844030614
0.3326715274320528	0.8201610783123442	0.3174870253729456
0.5825603868185084	0.8202906021664585	0.3175231910990119
0.8324391726788349	0.8199651464300697	0.3171400560767453
0.0824438531611411	0.8201275162667633	0.3173464887183490
0.2072631075362146	0.1013977874013195	0.2042152496549512
0.7073018435982666	0.1016340650898173	0.2041832317885058
0.9572228498625630	0.1013983245253766	0.2043953719073473
0.4574785315337540	0.1012782433678827	0.2045405261464656
0.5824902691737677	0.0666657866290758	0.1980241104307089
0.3323798208097291	0.0666646009147198	0.1978582076216333
0.0821378830002050	0.0666944639339900	0.1976641341767489
0.8320878532463281	0.0667081985772586	0.1980677683517498
0.3324360092656786	0.8703315168306747	0.2666670215591202
0.5825840702751428	0.8702331786089790	0.2665264670123392
0.0822852178489144	0.8708406230198745	0.2671413975821911
0.8323965830725598	0.8706013126707690	0.2669101526638051
0.7074799965765509	0.8980321732713051	0.2447501966335755
0.2073156646177788	0.8982834586409979	0.2451376516091331
0.4575492622468606	0.8983691021122566	0.2452091378665858
0.9573327671987644	0.8987713919398317	0.2457857961934836
0.5823628635766674	0.9953555028806420	0.2020573000585645
0.3322950816828905	0.9953267698502950	0.2020460900482401
0.0821498295819486	0.9953483386049340	0.2018948585100027
0.8320547616787173	0.9953441789986724	0.2019198241071321
0.2072427172262716	0.9614356080681384	0.2117094110779001

0.7072431956874647	0.9612397530282512	0.2114618218767851
0.4573430062639783	0.9615654320326515	0.2120700934755309
0.9571859762459596	0.9615592941350786	0.2120457272808693
0.7954334374486369	0.4876872534953983	0.3911779639102156
0.9029994204209894	0.5155738041863980	0.3984142167506332
0.6991860473498317	0.4638904781617110	0.3846319722602332
0.9967304742121436	0.5421084652874909	0.4021214284513078
0.7587425624561375	0.7493577057176505	0.0223399256492300
0.4814791739688580	0.4477463013018430	0.0828401407482920
0.8969496923336689	0.7221510798038805	0.9883116055659276
0.5400951339252039	0.4429748396167817	0.0089801809597154
0.4555314446924248	0.9596519304660188	0.8912973470525215
0.0750802225311467	0.9194075978108682	0.9037720972290744
0.7785921349831233	0.8049811133291873	0.9383187546744159
0.3945245346704310	0.7603716750436534	0.9764598810295336
0.3778541124565021	0.2359465371042260	0.9246758869689186
0.5752122496188121	0.2505321301706674	0.9606076592863192
0.6323464504516765	0.3122994903723645	0.1226868068710120
0.2328577530361522	0.8343119056859689	0.9983391884903198
0.0563600002668539	0.6571941097370544	0.9957691363388960
0.0928543340214527	0.7206340410652409	0.9470907997538844
0.3751879822037020	0.8668002320952412	0.9998706012110324
0.3297185838208535	0.1616800519360980	0.9188928547318046
0.5823515884756278	0.3360962414701305	0.8284288977324322
0.8525707443242675	0.3663326180805675	0.9353844142660476
0.1369694219386672	0.4265666329728922	0.0038428834667878
0.6954592152476267	0.7046670218559700	0.9055160142804570
0.1648278579914812	0.9935799049267630	0.6631530088519996
0.1927817379905519	0.9932732098468280	0.9910245424375352
0.8689410480348988	0.8652297891518336	0.9133461672052996
0.5483389291385918	0.0233106120104081	0.8819239329459702
0.0477414952284551	0.8710748279022379	0.9650651095066092
0.3226744550293590	0.8750020045444917	0.8981825542831704
0.3976753604440262	0.8603687806347543	0.8314463712013263
0.2156444732355054	0.0909218629822533	0.8004325456479288
0.0017446869127048	0.9557338374525732	0.7833888412806723
0.8452076556913256	0.9718996050106592	0.7678721883156784
0.9575850275347402	0.2778611026288963	0.9207813521268932
0.3142951342490329	0.0347690114754700	0.7788484815357586
0.8883697178639377	0.2754675482945508	0.9942316771573964
0.8773770615180133	0.6384590149048182	0.8565832964563455
0.1655760100659335	0.2508162625261081	0.8791764376640511
0.4986055251844731	0.4015357568276949	0.8503325852755761
0.9884549415841876	0.5467537561420759	0.8644588648217182

0.9346609506257116	0.4301824186008115	0.9602087581969506
0.2861369747709932	0.9712294630136320	0.0522530308524829
0.0199853771784504	0.0291507717428231	0.6465010675683073
0.6087907227091823	0.6369267443782637	0.8324296864808204
0.6123490325596840	0.6159113261630643	0.7561442080644387
0.1311927755993893	0.4393683599123498	0.8568919810229962
0.2268368310145595	0.3943389615165290	0.9016666023266140
0.1505914999859667	0.5456767279923710	0.8748132255410251
0.8915492314204224	0.0229319138108001	0.8623421578842505
0.8438381736853031	0.0894361453413267	0.9014415061347972
0.5090563311302341	0.3144012352899430	0.9253005078073112
0.7431824828552406	0.8309398186232995	0.8404390401930042
0.2040053401099365	0.9028469596294412	0.8046583710329061
0.7829027469961384	0.2104453694847019	0.9211611662303149
0.7784534887789795	0.5805565759641864	0.8466076790653841
0.1166675802822085	0.3249821714900010	0.8593275603892135
0.3104670622827747	0.6539228600245331	0.7867534459052540
0.3634985892453226	0.2816161353505486	0.7555178616911554
0.1021095235596556	0.8017245833735617	0.8772702512964262
0.2467913315676361	0.7096007736999617	0.8371520173838354
0.3590290667599493	0.2261028223403272	0.8135106441074597
0.0361986234057279	0.7384413058108913	0.8409705823591048
0.8840435694231985	0.4213046014825895	0.8413675262944719
0.9521570613747994	0.4089940678625876	0.7672873511385936
0.6801752994965142	0.0010521336237996	0.7122251663596212
0.9736550365578314	0.9258162154433141	0.6420442083287169
0.8190010425190226	0.0960194582321550	0.6675203180476770
0.1662902360513540	0.1431448447017228	0.6971031261165419
0.7775062896054709	0.3462150135321299	0.7879578085589694
0.1376015151351197	0.8712139790997165	0.7390706784555452
0.7546482822268160	0.8524199181030131	0.7642232513072050
0.4703822712628156	0.1052041939412576	0.8306830134158035
0.8401990729067864	0.7471160843850666	0.7972349988704138
0.9980727843934236	0.2472799294908521	0.7996584415022748
0.6088979334723379	0.1332710508384077	0.8652616439352395
0.9087591859643590	0.6847242135460750	0.7592547823007421
0.9481924751251936	0.2471133110346170	0.7227207655062752
0.7781018510622452	0.2675549916845713	0.7850785895004494
0.0663599348685265	0.5309556459149097	0.7694129194483075
0.3669667819802221	0.3963254029499426	0.6963269183446766
0.5294171897228376	0.4737107826463425	0.7058791588858528
0.5892410311072223	0.0951388701285228	0.6842871819695191
0.8222008208520870	0.9179149187870750	0.6681296395102356
0.6104602927500814	0.9276282704248670	0.7098068288892210

0.2875918351365085	0.1936800261741507	0.7157459159636265
0.9162706762406730	0.1265699530448054	0.6092592634085472
0.3801778392813117	0.0301034817714503	0.6768787275562437
0.4427803467683677	0.0806290912062694	0.5922100211019415
0.3691895474893515	0.9530551218138676	0.6962185779379818
0.7925955058736637	0.0441187961777392	0.5751916951411714
0.3639155992836179	0.1280330237612299	0.6486251034635979
0.6447435163396151	0.0279503809959823	0.5409071553193058
0.5605635449107732	0.7933657686689843	0.7975753199621731
0.8430501534885695	0.3031174621973085	0.6438917750357780
0.6739851678349665	0.3119558437619727	0.7002301636358510
0.3226482042525031	0.6648956473244615	0.6719319967126025
0.0587409001169089	0.4901713806915723	0.7014054984242070
0.3204399339085537	0.3334810363749537	0.6511421603455989
0.4233487615158114	0.4763808433858239	0.7636822229024489
0.6403937528012035	0.0947538182042808	0.7594192956431962
0.1082663559441047	0.0187508281314421	0.5569185284421913
0.4275806871369230	0.9851234728945976	0.5265215531050428
0.4478305472375250	0.0533201730360491	0.4858282972339267
0.1642600970304163	0.0798015333900621	0.5139414922857822
0.8777388020589226	0.1869628682159606	0.5225957172533228
0.8458130256349378	0.8548805453568802	0.5880192005260865
0.2529764247955857	0.2033865547705133	0.5206156940760905
0.3157858567664215	0.7050150779172956	0.6039779604288213
0.6322078841696696	0.2893610567667003	0.6259982588261498
0.9578527063037358	0.2589011849243836	0.6094855497522687
0.4208700793546974	0.7511349271753185	0.8008947284231863
0.6382541800408417	0.7227599532196591	0.7157779281401435
0.5234917041404301	0.6930183126764942	0.6721519257659265
0.6886672980300708	0.4160472357386964	0.6715314246349665
0.3636195268978633	0.5596078171780507	0.7100426708733173
0.7484557694887265	0.4520519823371961	0.7344221188196443
0.0170119841396647	0.3642891882003264	0.6732417569172080
0.1553736868499531	0.3838254986834648	0.7056610361128340
0.2089479229589919	0.5769153625687617	0.7115929817357559
0.1068969502441688	0.8691141105766120	0.5114721410519280
0.7840037929458524	0.8858032247173755	0.4824053132184661
0.7322761117618071	0.1150179625555866	0.5073083209588329
0.8509710155968703	0.7874964269780405	0.5436005348626922
0.2509533883617553	0.1544292910656740	0.4556820309052244
0.0392816425824053	0.1828828962217233	0.5265801311967345
0.9522290194258656	0.5988603119033065	0.6971421504489020
0.8124701636752301	0.6340785996807009	0.6790405783073918
0.3982391385576445	0.2524877503839080	0.5789192441718432

0.2652042962065888	0.2311333277450676	0.6173565471897127
0.2991526699484374	0.8855937685373292	0.5278518089720998
0.0188573287477277	0.0636470272306520	0.4327588496997790
0.7253749076463385	0.1484948053701115	0.4386349053223493
0.1411760173669120	0.8414495557559748	0.5873498265762632
0.7802297760637223	0.9643892983277270	0.4842423118487780
0.2221112246196494	0.7738736910435025	0.6731436115285535
0.4539766330345479	0.8473266872570886	0.7240171856645566
0.0654630154486809	0.7842418211138779	0.6811720894536177
0.6189490521402237	0.2050993485862022	0.5406506018679164
0.8358968296367394	0.0960851036268998	0.3697973542234994
0.6883263221374522	0.0641277908554629	0.3705422540107650
0.4902860915647100	0.8606748851536009	0.6477914180599856
0.5400515309988114	0.1813412392723278	0.6048099321262287
0.0957109272430351	0.0923311312334125	0.3720146887754823
0.4575118009430891	0.8760968826600138	0.5267284475362000
0.9639781945336406	0.9228794901753020	0.4386901253841631
0.3521942615620212	0.9381992964060210	0.4370298553798461
0.2348351223140850	0.9444579290387588	0.3854384466155726
0.0878318434902433	0.9647718900186434	0.4646065188969065
0.5113340622861057	0.7717961748643056	0.5796244766616875
0.6431416930245066	0.8169683439210697	0.5682948282032346
0.3088192212492620	0.0442352128430995	0.3922948204372703
0.3636719675180268	0.1078330668805009	0.3542749103836809
0.6601577093167632	0.9569307113099480	0.3981372033136779
0.5131327407257922	0.9736138955091604	0.3698989351784999
0.2479034125321269	0.4941841807468475	0.4156802210758822
0.0743370835908260	0.5434099079022351	0.2789209790017372
0.9840159903641156	0.7572670392734854	0.1451756020810641
0.1274403608655834	0.6184867168911291	0.2752728406477233
0.3054356820815482	0.4668615258456589	0.2243848769215724
0.8532686264377817	0.7963192081422922	0.1212358603187875
0.3511592380645459	0.5866634651216421	0.1947822405803584
0.2186453268457189	0.5398937051041149	0.3511770090564169
0.7653308775821593	0.6728335585695233	0.1769821768575740
0.5595591952542210	0.6087691995877208	0.3382570669773559
0.7647714326823438	0.5351219235558009	0.0601345178023570
0.4403096954821951	0.6082465246600781	0.2589440282886544
0.8018472778863588	0.6548401053969329	0.2524204560387644
0.7811473151596676	0.6074833857132393	0.3533557990354319
0.0355991320153117	0.4341607777109522	0.2173764204430135
0.1780074629034337	0.7117696944493084	0.0568283326122463
0.3593715960311361	0.7647055169128664	0.1101826109322910
0.8403629727147195	0.2861896216759778	0.1076631831991404

0.3081451069474143	0.4538380734825643	0.3021973766833229
0.7282782260845537	0.6313748723077627	0.9912700782754980
0.5528664774005269	0.6470427793373278	0.1581681080641378
0.6400033065183077	0.6370608548868083	0.0920800621657213
0.1300459097827072	0.7313244066065088	0.2431524898671900
0.6354752554124442	0.4987519590182010	0.1369965210580112
0.8635439584412775	0.4886817212604873	0.2559824897546251
0.0056017698558350	0.6892489424432101	0.2167321796364332
0.7106324846261574	0.4668829029896017	0.2543769383030524
0.5124715126210710	0.5328751298064791	0.1769785315340564
0.6116039715934014	0.7874913220240169	0.1038067967421806
0.0787435863781873	0.1518262650123207	0.9378679158877236
0.0038780097050532	0.1495124203087475	0.7648321968564986
0.3089700974258875	0.0791539465574986	0.0031127996942325
0.0355502104054427	0.4245223341264090	0.2960351894002212
0.8867749570025544	0.2135796252607922	0.0846095126507478
0.5269266575570273	0.9292987604661116	0.0490482692544974
0.1925513575560754	0.6957406973029283	0.1353055635396578
0.4341071642440539	0.7925596358619894	0.1731420721165542
0.3015674776427126	0.3637150242671381	0.2112395544004387
0.6610960958563794	0.5582841562016293	0.9828879608465984
0.7402372749323487	0.5673143202096520	0.2885641179052683
0.7881056942208361	0.4572510337497402	0.0694248448129923
0.4223777658572972	0.5707285276936539	0.3599959560153722
0.7110998237418313	0.9015994740438120	0.9845741837221890
0.1262499601474261	0.1891548801339523	0.0032903037764631
0.9287258186769752	0.1419376629149709	0.0198375057164523
0.4266614489158647	0.0528696338246162	0.9556375791973430
0.6974869439299092	0.8460714347602506	0.0683859355240364
0.1195611345184121	0.9071135357496636	0.0574700360230262
0.0395960694442465	0.8379537106722494	0.0779490439806289
0.4298661035546539	0.8824107505046395	0.0920705288595122
0.7759935436311995	0.9481696127701004	0.0399986629832755
0.6497308171231015	0.1473933573207799	0.0597394568826678
0.3083200678002808	0.5810665426090121	0.9589190913366892
0.1365577551332763	0.3690299150701961	0.1495776680973148
0.0129818016641552	0.5852913300074069	0.0726759124252976
0.4784640133093039	0.2994172225881323	0.0532528160231113
0.5268446522942424	0.3895835886097558	0.2680506994578342
0.9114417935169136	0.5321821768877582	0.1422103018644187
0.7235681207430680	0.4579570300150162	0.9416779012265322
0.5010743891816891	0.1329068895643385	0.0299160764685363
0.3467459227493181	0.3958345541683869	0.0089974786376170
0.2375884008213204	0.3352447681969008	0.0038647789472821

0.9297436493355437	0.6011751409159960	0.1789884576563324
0.0382624252565058	0.4346434580550169	0.0642673874827999
0.2800101312599607	0.5345055463362837	0.1150259139659376
0.5814707754369972	0.4859949080784072	0.9118440767289178
0.5299856872898449	0.5350840182771971	0.8269529529083296
0.4013207076706507	0.5208063488950765	0.8722097595297199
0.9029279862467782	0.0158747354916785	0.9870055512659588
0.0009722969957387	0.9991358110432960	0.0477494657804534
0.2159987603316023	0.0204921220937494	0.8761976078490329
0.0698073390093872	0.0287982168303183	0.9086622502714908
0.4951715211026905	0.7159691288582619	0.0183711199107695
0.0881957945625228	0.2659818598373346	0.0687215821530795
0.3504892186724054	0.3179150137954916	0.1499427050947341
0.9774932125845960	0.3580601078831817	0.1456177423924324
0.3168069039497280	0.6194430030345985	0.8880780883810392
0.5013979307737553	0.2330960672669389	0.0914214804914760
0.0442919541183803	0.5375067832964555	0.0131649327430148
0.6996409726510638	0.3470803619751345	0.1835336117868235
0.7440602902134501	0.1502765164212645	0.9661611934654832
0.5393203526010323	0.7055363106096140	0.9115012469943322
0.3198977562121480	0.6290162293254800	0.0595321271642426
0.4516123066033370	0.5901915289402228	0.0382970462859532
0.0050040198816059	0.0683000315746351	0.7646390674295737
0.8498889945986453	0.0869762572812875	0.0577103193137791
0.2439904391563148	0.2491580394217147	0.0788618367644010
0.5704027364604526	0.4304324108487678	0.2064674805189589
0.1475088812647139	0.5398201168970508	0.1599662301361685
0.4800232039310980	0.4199792873095900	0.0418556046590431
0.7964190324802298	0.7276643479642199	0.9811727730212196
0.4412541779531107	0.7157869186076388	0.9776172431560740
0.1759495380314188	0.2593959758927706	0.0449356387645109
0.3165575872342940	0.2060692999787234	0.8977145981221157
0.4980210090002786	0.2809453829492436	0.9632256280455876
0.3200360463996936	0.8341948345860923	0.9730724990864592
0.0628940984342038	0.7077348033773093	0.9931497374275228
0.7220816393884657	0.3290964173862735	0.1388659424033030
0.7714416806851706	0.8497763371908914	0.9164224307868964
0.5121829349302467	0.9924279866280946	0.9162097569838844
0.0318782738092938	0.8761667090927856	0.9156958306394049
0.0745809179421136	0.5229864992184721	0.8508396150285726
0.6138893257572201	0.7010754458549299	0.8775906684882554
0.8618863101432827	0.4156003970507748	0.9281536144858048
0.5071352850413313	0.3531365196050906	0.8567569181381467
0.2974178562041176	0.0664277137677749	0.8165990266070016

0.8688361755908590	0.2813480101234844	0.9456394873040764
0.9273781761029360	0.9905160903015556	0.7886810452974784
0.3486479113608184	0.8971214455164731	0.8551828625843753
0.0818888674327553	0.9916429711610252	0.6345411389314303
0.1977391582449243	0.9922940777614334	0.0411009273073276
0.3535573595191712	0.5848048989969755	0.0433073232254356
0.6179618638930178	0.5965344954706174	0.8013901093077802
0.1839651543638336	0.3971448890964976	0.8567834442017758
0.0838852437069516	0.2770056293418705	0.8663553867809058
0.8551884230279434	0.5938542733666635	0.8754165107968120
0.8930067523072640	0.0467985833414052	0.9066524695390714
0.3299570004035463	0.6831287206585683	0.8257138545812258
0.1164029442014261	0.7532896925691085	0.8685423525542193
0.3781353195966808	0.2337591676275610	0.7654377154654128
0.8657496969160348	0.4150825118779899	0.7928010991930806
0.5698228489671315	0.0998777633458272	0.8341310559470643
0.1213214306103415	0.9027283159432312	0.7762029136943618
0.7349550913917648	0.1663003487605335	0.9196107325946292
0.7328150245751386	0.8141616389798672	0.7925045307472153
0.9051398830634956	0.7094347328669480	0.8033329788077510
0.9357551061315776	0.2245402304274534	0.7671541928643172
0.7154270235747847	0.3063231271113305	0.7833434366829019
0.8872618228075698	0.0848196958518479	0.6327080475089908
0.6989600358774982	0.9516086798976452	0.7155219022689706
0.8987655212660159	0.8925314214165161	0.6474962027696374
0.2408378216049174	0.1695198846694921	0.6767686060345670
0.3172790662612298	0.3528932203241064	0.6964466555517269
0.0626043669608838	0.5365925619340735	0.7191093235002413
0.4285198009226150	0.4776606494413329	0.7141533283576048
0.3226629572828454	0.9973084257448482	0.7005800603364051
0.7437826796067238	0.0313264975153669	0.5331321094525637
0.4525542627224259	0.1053192101536845	0.6356744040181569
0.6697066727705038	0.0842437406801144	0.7124284266975485
0.4620520761497185	0.7949990641709092	0.7876810302301215
0.6798546978343075	0.3247177651967663	0.6518774935673177
0.3453058490868873	0.7097699455337451	0.6503359127676208
0.9411724911944854	0.2900118191346016	0.6475086730341398
0.1189792657506818	0.0359205500118263	0.5101985627374944
0.4674812545159006	0.0317752662955035	0.5284781204741081
0.0598363561097356	0.3999218723254600	0.7009306442957562
0.6158039957387833	0.6834822999117037	0.6890583013974749
0.6906351524965202	0.4596705534109778	0.6946707337038227
0.3009217940630515	0.5986887135593941	0.7145219023092417
0.2062027777243662	0.1653881664611196	0.4983271502344350

0.7993915585297504	0.8285140486505204	0.5496689280631705
0.9568466499181574	0.1917182144569864	0.5535582454614013
0.3000638217878502	0.2630398598395992	0.5832159412627295
0.9073247472830812	0.6430758067610605	0.6896117516972770
0.7257737609834408	0.1609789945948787	0.4881210358908289
0.1700318699080788	0.8427195306113749	0.5401798898584199
0.7865307274857474	0.9255557803562814	0.4527437561402948
0.1499102414807735	0.8084930386887310	0.6744799714618067
0.5651410500921594	0.2221208170468779	0.5795321940678437
0.7380659040318638	0.1073580293458493	0.3703430181676120
0.4681147512157987	0.8839697319691583	0.6905100027848080
0.0052468361524906	0.0779012880614535	0.3861556745921313
0.3869705657332633	0.9086852855964380	0.5131290974080249
0.0640490566656552	0.9209037537729088	0.4445250401127513
0.5424957964468795	0.8179326682076686	0.5783758600427436
0.3263551582874316	0.9614055990342794	0.3943660904201368
0.2993007135456975	0.0941589793478911	0.3884460239495552
0.6073875476172784	0.9888231987589012	0.3705459583017307
0.4393151422386865	0.6067589756983222	0.2083983384905841
0.1101523390716898	0.5808232184548510	0.3060457339445451
0.2854778480132757	0.5081718380951417	0.3729230031927830
0.8437935113633729	0.6700933058148014	0.2093463079285891
0.7817644545723388	0.4993089591180450	0.0936727192734864
0.7253434258155474	0.6122820993171011	0.3134054323697421
0.4587667668350734	0.6115265083965926	0.3392391479747501
0.3344798933552555	0.4341918648885215	0.2587595353504499
0.9517470832175214	0.8012436123517310	0.1320691590333239
0.1073329993489816	0.6919003941994509	0.2165889513547439
0.7705833327281907	0.5059632152240437	0.2468640817222642
0.5413198201974171	0.4911226508084091	0.1537418766406594
0.6254423171301101	0.6675821266063603	0.1301924107850096
0.0211165867866237	0.4576762339703223	0.2612287490410519
0.9131013134219448	0.2612699919528970	0.0818074745799500
0.2449219965829794	0.7031668286665222	0.0937103952316492
0.4291368323145676	0.7982612530561133	0.1248802912970354
0.6729181992017110	0.5975522095284526	0.0143192149822884
0.8413605309839555	0.1349320604414796	0.0456565008793496
0.2700815751177270	0.3328127669713257	0.1757301225346705
0.7017194958536032	0.7986336742179285	0.0852718587470477
0.0775916856065250	0.1487161559289454	0.9863534703150880
0.0631671329923464	0.1086682895937658	0.7616142540071156
0.3876270724233014	0.0943243884524577	0.9773784969942368
0.9818358999264686	0.5647079299293999	0.1565708083431246
0.6076586410872301	0.3985580224258260	0.2413474360624471

```
  0.6389194041441539  0.4839034918759308  0.9531780843775302
  0.0959408119029507  0.8615844433855938  0.0426598036672980
  0.4300422956477413  0.9195773987734012  0.0591941060926574
  0.5548774081777235  0.1613984574345106  0.0610310213018006
  0.6975108739467009  0.9180200799928868  0.0310765891886587
  0.2611290514197432  0.3796720342345075  0.9854031018697376
  0.4764041922296152  0.4970248774035407  0.8480971018454398
  0.2447961722266808  0.5266959809689669  0.1606605761806225
  0.0511311251082853  0.4508110316038024  0.0177295110867407
  0.2944500694282409  0.5759501457661642  0.9097009316099725
  0.0237409192392889  0.5850394402713345  0.0234585090192085
  0.4773236504396372  0.2813111667443972  0.0994300175231708
  0.0460085493718363  0.3931416858167366  0.1440911515553439
  0.9049242925391436  0.0042840863846267  0.0356424667070035
  0.1517982601370570  0.0002040314685987  0.9095087733028586

Adsorption of C2H2 on C model
relax
   1.00000000000000
     9.8709000000000007    0.0000000000000000    0.0000000000000000
     0.0000000000000000   17.0969000000000015    0.0000000000000000
     0.0000000000000000    0.0000000000000000   20.0000000000000000
   C
    64
Direct
  0.7499989512044466  0.0001088985635440  0.2499999974562424
  0.5000013820157330  0.0001403956447164  0.2500000051286347
  0.2500010551576434  0.0001003839049797  0.2500000009418950
 -0.0000013745501476  0.0001204802004805  0.2499999928932520
  0.8750559749205601  0.0415731431231060  0.2499999892346353
  0.8750625751378330  0.2915898198753586  0.2499999919674222
  0.7500021708013553  0.1665450461312343  0.2499999963412259
  0.6249421025707482  0.0415755655882879  0.2500000014419251
  0.8750684915383340  0.5416066072255818  0.2499999986359753
  0.7499987948601836  0.4165207317779543  0.2500000047290154
  0.6249413523867953  0.2915911342645600  0.2499999963235928
  0.5000000281749235  0.1665193055523660  0.2499999980558778
  0.3750606681359456  0.0415701395793224  0.2500000042196879
  0.8750601597618363  0.7915929295426908  0.2500000024770622
  0.7500024868898524  0.6665703549021119  0.2500000004099039
  0.6249315637954641  0.5416093804780658  0.2500000055691329
  0.5000011043326812  0.4165092173324810  0.2500000041972950
  0.3750600226007482  0.2916041251507479  0.2500000017071564
  0.2499978546518334  0.1665546915158900  0.2500000073649934
```

0.1249412612294882	0.0415676992009589	0.2500000000670564
0.7499979399002139	0.9165457911419452	0.2500000065473494
0.6249434130716683	0.7915957482561821	0.2500000008132905
0.5000020821818939	0.6665507124731354	0.2499999939228543
0.3750697415439240	0.5415974260272327	0.2499999983603671
0.2500012241282946	0.4165736111824636	0.2500000008094344
0.1249361032928352	0.2916028283548526	0.2500000033868646
0.0000000162329872	0.1665210986862351	0.2500000042655977
0.5000034641287923	0.9165237670379266	0.2500000000217864
0.3750574625394046	0.7915971849520732	0.2499999914127898
0.2499975179474937	0.6665667736942468	0.2499999960826611
0.1249303166387075	0.5415946450056121	0.2500000010262095
-0.0000010906308085	0.4165053115362170	0.2499999961691245
0.2500020149946470	0.9165465757242344	0.2499999935465849
0.1249387506969472	0.7915943709092154	0.2499999981437881
-0.0000020658279724	0.6665497785186453	0.2499999970975293
-0.0000035024712760	0.9165447639294606	0.2500000013362415
0.8750634914319685	0.1250633381528638	0.2499999968632693
0.8750629667138266	0.3750790576377634	0.2499999963876783
0.7500025996979647	0.2501057330826949	0.2499999934382922
0.6249401206789126	0.1250616343548054	0.2499999994596837
0.8750641407871004	0.6250957710123135	0.2499999993968481
0.7499982124975325	0.5001639581647136	0.2500000066665005
0.6249376703781655	0.3750765816211014	0.2500000052251045
0.4999999503178891	0.2501369440442160	0.2499999984043010
0.3750622872032835	0.1250529265494996	0.2500000044145770
0.8750671442379481	0.8750784649819913	0.2500000083091090
0.7500026994628268	0.7501203352990327	0.2500000017415598
0.6249400947072082	0.6250924965837524	0.2500000010436533
0.5000015906007478	0.5001656217246073	0.2500000046237053
0.3750631882595443	0.3750931159697136	0.2500000023798967
0.2499973907156228	0.2500965084156402	0.2500000067219252
0.1249340891563495	0.1250546363324405	0.2500000089692520
0.6249323230529689	0.8750758609873870	0.2500000059700324
0.5000007692831907	0.7501328806948421	0.2499999949693631
0.3750618442060641	0.6250866408249789	0.2499999946682554
0.2500018649720671	0.5001210074667823	0.2500000015604864
0.1249361846794511	0.3750955934329538	0.2500000008173370
0.0000000152889072	0.2501346347278319	0.2500000008452322
0.3750651797707331	0.8750777860271032	0.2499999921722452
0.2499972232803573	0.7501235503446009	0.2499999956033361
0.1249340258221163	0.6250899070098664	0.2500000012436661
-0.0000015081690543	0.5001691045079277	0.2499999976980910
0.1249352996931398	0.8750803924044427	0.2500000003821921

-0.0000008727108437 0.7501350806601265 0.2499999979199512

adsorption:
1.00000000000000
9.8709000000000007 0.0000000000000000 0.0000000000000000
0.0000000000000000 17.0969000000000015 0.0000000000000000
0.0000000000000000 0.0000000000000000 20.0000000000000000
C H
66 2
Direct
0.4596204494407827 0.5469845544295502 0.4042882225578475
0.5591592585266317 0.5058863909046972 0.4014448538499634
0.7507192979753254 0.0000331133286524 0.2520858226285568
0.5007555206446441 0.0000124705156837 0.2520465025047323
0.2509031952193575 0.0000294061352404 0.2526276214297565
0.0007863087500364 0.0000289528408005 0.2531444881406458
0.8757822062390679 0.0415948165249732 0.2527381844969691
0.8757041569456530 0.2915786754374108 0.2459665823031409
0.7507123814027944 0.1666068895123694 0.2500087456977944
0.6257169232435981 0.0415648910971506 0.2520379424889805
0.8756412482691527 0.5415179538455257 0.2405402469958227
0.7505530026152228 0.4165478187367240 0.2409165611803435
0.6257939063200006 0.2915799984899510 0.2445713261545344
0.5007598259720004 0.1665869516148887 0.2490612052392306
0.3758736286251882 0.0415928800568033 0.2522947392421716
0.8755633753642634 0.7915460075460247 0.2467911923269727
0.7504962883086953 0.6665802056009812 0.2416076640140705
0.6256250854429446 0.5415440953725144 0.2377278892921915
0.5005986636477078 0.4165403651621568 0.2391039162489197
0.3757643139304241 0.2915804694310399 0.2447727500136580
0.2507926637912803 0.1666195026413212 0.2501564078048295
0.1258574785749917 0.0415980664449955 0.2531012772801285
0.7505898463903848 0.9165263466111933 0.2506135092957368
0.6256062836084020 0.7915199479130769 0.2460017818439422
0.5005302914284969 0.6665395002914519 0.2405242943648003
0.3756378889916865 0.5415238558611337 0.2379077988867075
0.2506394823783281 0.4165256922342501 0.2412377128901420
0.1257427077657436 0.2915554929544584 0.2461813331447129
0.0007572008024710 0.1666728599431036 0.2509352740031966
0.5006109915029986 0.9164752320294877 0.2504364394870197
0.3755736923613932 0.7914675728642203 0.2462664393115282
0.2505162218739615 0.6665470185303818 0.2415991036951933
0.1255350660336970 0.5415166463527896 0.2408163851889379
0.0006016832462210 0.4165166542435774 0.2428962869470423

```
0.2506793670953553  0.9164710344039853  0.2511003439617629
0.1255572489071711  0.7915254314099841  0.2467932813427171
0.0004953092778903  0.6665668780273400  0.2428581586560136
0.0006106484666644  0.9164906970113521  0.2517644811548307
0.8758122630514927  0.1251182550742068  0.2517137377343017
0.8756065633893229  0.3750278219499394  0.2433213158550576
0.7507591680265518  0.2500651899606268  0.2469208593502900
0.6257228479558228  0.1250767797715947  0.2506781641535528
0.8755930380275718  0.6250678780730300  0.2414479145643693
0.7506242753941229  0.5000396034443731  0.2393152997047628
0.6255541914230620  0.3750333481487260  0.2411735231069048
0.5008287871236677  0.2500631756869393  0.2460356657478964
0.3758225306960182  0.1251003003695864  0.2506845546435390
0.8756169773101932  0.8749897293098453  0.2499781894349870
0.7506305478280807  0.7500434418468566  0.2447621091415570
0.6255263826326631  0.6250484673906187  0.2393871783424126
0.5006241331056098  0.5000277908658760  0.2371016838161898
0.3757198692887084  0.3750396498564300  0.2414134319577364
0.2507913606611512  0.2500883415972077  0.2471620497168785
0.1257826778210349  0.1251476963070332  0.2519273351122693
0.6255490095108891  0.8750034259513653  0.2489849533448421
0.5005992048852513  0.7499827917414039  0.2442066928672087
0.3755641158400402  0.6250231201566806  0.2394471106650124
0.2506379186373357  0.5000237536257877  0.2396909905951203
0.1256725322720064  0.3750288569569998  0.2435295143767169
0.0007315512320187  0.2501057921675630  0.2478744527309140
0.3756775162074640  0.8749447654535586  0.2494467577252432
0.2505448463168974  0.7500029800510968  0.2448184694216926
0.1254995738237940  0.6250259118007895  0.2414923616947759
0.0005923327905219  0.5000203667607389  0.2415292940150714
0.1255772047909641  0.8749687393112829  0.2501592739730067
0.0005671793532119  0.7500466598999329  0.2453342147053533
0.3709801437937050  0.5832275206126571  0.4060676826614432
0.6476981474301937  0.4696725095061182  0.3978284527753480
```

```
Adsorption of C2H2 on PCC model
relax:
   1.00000000000000
     9.8709000000000007     0.0000000000000000     0.0000000000000000
     0.0000000000000000    20.0000000000000000     0.0000000000000000
     0.0000000000000000     0.0000000000000000    20.0000000000000000
   C
    96
Direct
```

0.8749959391136006	0.5384753327731215	0.5449168737945419
0.1249981327839920	0.5384769149515114	0.5449177387784316
0.6250064071472632	0.5384769920951131	0.5449177368411725
0.3750019414689315	0.5384771014290723	0.5449188169561894
0.5000047286019269	0.5028260282223495	0.5461272487611218
0.7499961070420396	0.5027868329894687	0.5460324489649344
0.2499994018506228	0.5027862307955865	0.5460357102881702
-0.0000024667608849	0.5028300337677140	0.5461110566583161
0.8750322056064066	0.6065655283810286	0.5250466453202611
0.3750338929344368	0.6065643390091161	0.5250419099957254
0.6249610501083562	0.6065639538811867	0.5250409084485009
0.1249591584901111	0.6065664128914314	0.5250466131525839
-0.0000000343909113	0.4334596916634728	0.5309791743950257
0.5000005920315093	0.4334636333438198	0.5310013991293836
0.7499981352121428	0.4334064023042740	0.5310362290594098
0.2499995456914809	0.4334082484820808	0.5310373458354943
-0.0000048938884531	0.6374611923725799	0.5072724685983763
0.7499967074468441	0.6373636605984906	0.5072358081084374
0.4999983179585029	0.6374564290814025	0.5072651548937768
0.2499959964280837	0.6373638357649095	0.5072374545446873
0.6249870043116043	0.4010546744978754	0.5159881840736984
0.1249872735856013	0.4010603866159543	0.5159779783332580
0.8750110486673767	0.4010605183646726	0.5159758115804265
0.3750097753145327	0.4010559856226769	0.5159890535867669
0.1249931201436926	0.3430914473701712	0.4751276619815164
0.6249911115546557	0.3430885890866715	0.4751323534117660
0.3750124351663788	0.3430879430619165	0.4751340685851008
0.8750065232634214	0.3430924439448757	0.4751273829010512
0.4999987061518064	0.6904184316573181	0.4601509467981632
0.7499941768361252	0.6903101143372635	0.4601182697080262
-0.0000020372957575	0.6904292985054384	0.4601564594348372
0.2500016061323315	0.6903095145066045	0.4601196646015753
-0.0000017998509790	0.3172275243858288	0.4506477727477121
0.2500036236885039	0.3171939995933679	0.4506792990554326
0.7499960211790385	0.3171960593455430	0.4506765877535485
0.5000024631742043	0.3172262479672656	0.4506504717712866
0.3749666581815443	0.7127892172656410	0.4325132856827458
0.1250352905566318	0.7127954679917877	0.4325141078554900
0.6250321900738482	0.7127880448634077	0.4325108818610802
0.8749602332488701	0.7127908057288997	0.4325125274504750
0.2500002598398295	0.2704217732190302	0.3973646322735162
0.5000042429594380	0.2704088574598476	0.3973974300067523
0.0000044013123280	0.2704092052044386	0.3973966593701841
0.7500078267263054	0.2704193951636517	0.3973663644462831

0.6250087731909483	0.7525687989442313	0.3738919573145718
0.1250028545453442	0.7525801260005750	0.3738965504777680
0.8749899337200554	0.7525760835121925	0.3738934676744087
0.3749858361712610	0.7525704582155571	0.3738938034435657
0.8750246734637322	0.2484578901226520	0.3693853746362564
0.6249851748924800	0.2484514828099973	0.3693925944860483
0.3750244263914885	0.2484541038055870	0.3693912138845775
0.1249834165038094	0.2484589222713514	0.3693827255350738
0.2499926287008312	0.7714288892126820	0.3437545211144524
0.4999957137449594	0.7715398065082890	0.3438510195528044
-0.0000035502381539	0.7715469912769927	0.3438491587622960
0.7500051525024246	0.7714264788533206	0.3437543574385243
0.3750597763762796	0.2043064009716926	0.3138912798646885
0.6249442944605431	0.2043047988021391	0.3138942425596804
0.8750590895799341	0.2043119648867519	0.3138875140116372
0.1249484908257068	0.2043153006564167	0.3138823730035498
0.7500095998633670	0.1807602399440033	0.2871648491539845
0.2499974651723230	0.1807651921314185	0.2871628915253099
0.0000042891428377	0.1808904172434346	0.2870269047644212
0.5000067886135870	0.1808816107814808	0.2870354508868397
-0.0000010416852251	0.8126956437018484	0.2862654371995488
0.2500047868335722	0.8126107401536781	0.2861027663919413
0.4999959548470925	0.8126951441107687	0.2862683059131743
0.7500001167522446	0.8126102524392742	0.2861022626596090
0.8750208975217230	0.8365302035120453	0.2598107526028945
0.1249775090355333	0.8365316607621139	0.2598116567307753
0.6249789616285761	0.8365299719065058	0.2598173545825769
0.3750185767495395	0.8365324056816621	0.2598204349767771
0.7500000330156404	0.1280950578466671	0.2396364444411932
-0.0000013644807535	0.1282186097316267	0.2394956236964498
0.5000021430331166	0.1282164706370805	0.2395002309084369
0.2499970402948985	0.1280926412396065	0.2396386988350205
0.1250034034818096	0.0981194302673099	0.2203209041404327
0.3750001569842756	0.0981207497093537	0.2203209968331035
0.3750453162675310	0.8932042465329546	0.2171971561048902
0.8750487533346553	0.8931984989059190	0.2171874328644302
0.8749892599265993	0.0981180419327802	0.2203187567390654
0.6249472713451601	0.8932040789775820	0.2171953031891631
0.6250065265457495	0.0981206042219895	0.2203200097760172
0.1249521387337356	0.8931991563676493	0.2171897446104792
0.7499992629665651	0.9258922462897422	0.2028856239029046
0.8749653647782164	0.0314979129995781	0.1958165439184009
0.6250374393627522	0.0314956500887927	0.1958266501971490
0.2500004174225196	0.9258939856523712	0.2028881921711606

```
  0.3749665398724267  0.0314960851616429  0.1958283481825811
  0.0000013546496288  0.9258804795705718  0.2027599545794538
  0.1250389564241005  0.0314970153752257  0.1958218493478498
  0.5000010882813906  0.9258750785760766  0.2027803678419605
  0.5000036518816003 -0.0039923123122966  0.1919041274072015
  0.2499986296813444 -0.0039976201038615  0.1920371392007401
  0.7500011788615847 -0.0040003612778862  0.1920319810111975
  0.0000018282313064 -0.0039891329670693  0.1918901011577702

adsorption:
   1.00000000000000
     9.8709000000000007     0.0000000000000000     0.0000000000000000
     0.0000000000000000    20.0000000000000000     0.0000000000000000
     0.0000000000000000     0.0000000000000000    20.0000000000000000
   C    H
    98     2
Direct
  0.6242580828931307  0.5361031547373761  0.5473538572420829
  0.8742672030258114  0.5360671284049491  0.5475073708484288
  0.1244209880786366  0.5360641180557600  0.5474218116971545
  0.3744038739972644  0.5360885446387776  0.5473606846529238
  0.2494223386352601  0.5004137083229466  0.5491824103564015
  0.7492457518077503  0.5003932883766100  0.5492330174044349
 -0.0006884680170877  0.5004281895102013  0.5493764045340706
  0.4993089472402985  0.5004387791524734  0.5492019358656440
  0.8742959634898361  0.6037853885548239  0.5260226403318573
  0.6243532391273701  0.6038601898419703  0.5260849027582720
  0.3743848951310776  0.6038137893485596  0.5260117270265925
  0.1244100664187574  0.6037799026613105  0.5260411044805187
  0.7492505005516695  0.4307845475747470  0.5351170516806213
 -0.0007029288624009  0.4308795283009649  0.5351197214410683
  0.2493469081136397  0.4308010530036810  0.5350905108252825
  0.4993490887868250  0.4308663982026427  0.5351961601248224
  0.2493780560443207  0.6344552049808513  0.5077330390679550
 -0.0006427680820616  0.6344203170957111  0.5076915198975966
  0.7493736941412474  0.6344625736694147  0.5077472025597660
  0.4993739486326146  0.6346189220624805  0.5078493195580586
  0.3743597506651889  0.3984303338029311  0.5201182192023059
  0.6242881592239594  0.3984722196202252  0.5200653139881565
  0.8743090478131810  0.3984709119259025  0.5200486083774496
  0.1243074495756097  0.3984940112837620  0.5199787211167656
  0.8742136375697295  0.3412678285045033  0.4780547012252857
  0.1242274347381899  0.3412603262745841  0.4780499843846153
  0.3741836910876923  0.3412969704627154  0.4780490681822108
```

0.6242339611916683	0.3413492125774703	0.4780075811174846
0.2493671134653067	0.6874353741268950	0.4605185837726079
0.4993508666047525	0.6876261390031655	0.4606854565152393
0.7493373387264565	0.6874332870630834	0.4605226020156277
-0.0006776398181771	0.6873686916821917	0.4604665698807573
0.2491492033312769	0.3161294087968252	0.4528621314637952
-0.0008208109140783	0.3162097823439931	0.4527587066744856
0.7491447093647648	0.3161912968761385	0.4528161619876696
0.4991848852439552	0.3163933532118200	0.4526154076123650
0.4741618288909951	0.5226696593784772	0.3585616473279439
0.5966049763760980	0.5224585759606318	0.3559889313642729
0.1244065390899497	0.7103502896733445	0.4332765231472528
0.8742673463104905	0.7102700771291816	0.4332050605441475
0.6244131766243496	0.7104500610478475	0.4333634591970471
0.3743272968134825	0.7103925608513577	0.4332749708137487
0.4992128326167887	0.2714405747445649	0.3978247480684511
0.2491112508848904	0.2711204599463621	0.3981160469363654
0.7491911087226577	0.2711486058445051	0.3980876634666347
-0.0007588582774540	0.2712533564624373	0.3979856006638938
0.1243552498735703	0.7519018124963670	0.3758567691400843
0.3743339339323626	0.7518500218668083	0.3758015096466020
0.8743871316476083	0.7519608751167988	0.3758592142389769
0.6243684000131651	0.7517471038593905	0.3757708420167994
0.1241816008775578	0.2501163542585096	0.3693491380030179
0.8741952604006090	0.2500996252507624	0.3694104711111398
0.3741106284453523	0.2502099373566842	0.3693116316793750
0.6242429916608137	0.2501910344160033	0.3693054323158118
0.2493349630156587	0.7719568774366353	0.3464929756534998
0.4993871141230315	0.7717727220292369	0.3463477763894198
0.7493304545256425	0.7719031379215125	0.3464354264104217
-0.0006172229311147	0.7720426607049404	0.3465040586527286
0.1241311903470382	0.2072006631500511	0.3128794695555237
0.6241166382346789	0.2071613905232476	0.3129693822072385
0.3741900914818139	0.2072122910312365	0.3129406539888421
0.8741604151775944	0.2071870712020177	0.3129622440188898
0.7491352634614877	0.1838714863108152	0.2860235623405527
0.2491837722117169	0.1839256555412226	0.2859581024909998
-0.0008859795195278	0.1839516167126679	0.2859063317371237
0.4991549448372086	0.1838791862987565	0.2859917061523546
-0.0006409546774839	0.8150229000849045	0.2901583833270360
0.2493568716864171	0.8149653206240078	0.2901407507454329
0.4994192990942333	0.8148601170691244	0.2900550416442454
0.7493399776635864	0.8149229077749797	0.2901260072989978
0.8743017414729349	0.8393396417605357	0.2641690519209288

```
  0.1244034445678945  0.8393390243015812  0.2641389029373876
  0.6244060608831346  0.8392915298628759  0.2641173636744553
  0.3743284513228489  0.8393038727303456  0.2641715514348255
  0.7492172578680125  0.1310864446395843  0.2386617091772433
 -0.0007345679439116  0.1310959451563171  0.2385825666065118
  0.4992229987223853  0.1310518364493275  0.2386425961751807
  0.2492268268172580  0.1310803494700151  0.2386523459344855
  0.1242691376603726  0.1007830799387484  0.2198506506384463
  0.3742410116423840  0.1007954752914794  0.2197900727830642
  0.3744001910734393  0.8959940126812466  0.2215549054203440
  0.8743794962558834  0.8959709598301835  0.2214431650344545
  0.8742642118574021  0.1007969728563505  0.2198032767034296
  0.6244212820093974  0.8959826916319530  0.2215475541903834
  0.6242597473337705  0.1008116963076420  0.2198326170979621
  0.1244341028585056  0.8959413139141589  0.2213892977105257
  0.7493846374775064  0.9283505482201454  0.2066186283934739
  0.8744019294403708  0.0336725869901543  0.1967592937587158
  0.6243175112612662  0.0337427925788759  0.1967169113952026
  0.2494017801067703  0.9283429684509458  0.2065648178766321
  0.3743918231176591  0.0336866840113649  0.1967147041871181
 -0.0005673850155738  0.9282948846371963  0.2065571272590451
  0.1242946549205643  0.0337075893868312  0.1967826575856928
  0.4994321529775671  0.9283450508150880  0.2066744180510666
  0.4993985992248028 -0.0018477222853525  0.1937331710441689
  0.2493374124708286 -0.0018401134750189  0.1939386289736678
  0.7493319906411784 -0.0018370697191761  0.1939425858638769
 -0.0005961828109469 -0.0018950321720133  0.1938248610321430
  0.3655672042903487  0.5228182679827648  0.3608636098206992
  0.7052147648372393  0.5224261868279487  0.3535992151536605

Adsorption of C2H2 on Cu model
relax:
   1.00000000000000
     10.1184999999999992    0.0000000000000000    0.0000000000000000
     -5.0592499999999996    8.7628780481999993    0.0000000000000000
      0.0000000000000000    0.0000000000000000   21.1963000000000008
   Cu
    64
Selective dynamics
Direct
  0.9986150081721609  0.5020385226276399  0.3827538146958953   T   T   T
  0.4985115068569946  0.5018986101731231  0.3827606450964764   T   T   T
  0.4985685357316535  0.0021265052180372  0.3826809360077824   T   T   T
 -0.0012322518247260  0.0021479097092335  0.3827639823934555   T   T   T
```

0.4984934643367508	0.7520373424740284	0.3828502278547791	T	T	T
0.7485986035232951	0.7520871739599354	0.3823971525091355	T	T	T
-0.0012273784301620	0.7520669895885722	0.3828319196251581	T	T	T
0.7486799236455058	0.2521019627158317	0.3829912747706288	T	T	T
0.2485962113874497	0.7520016028878050	0.3829972927985399	T	T	T
0.7486347768550566	0.5019331262552970	0.3828355258433951	T	T	T
0.9986576408650728	0.2521748641238508	0.3825753152487182	T	T	T
0.7486503729466367	0.0022024428127187	0.3828566272057782	T	T	T
0.2485726677398991	0.2520898138116817	0.3829969401079873	T	T	T
0.2485058842288372	0.5020209631843671	0.3825774903524417	T	T	T
0.4985485152146201	0.2519436481853894	0.3825723868548143	T	T	T
0.2487199155494581	0.0021195259663792	0.3825830704718394	T	T	T
0.4153132212619144	0.3346853108986935	0.2857288332734167	T	T	T
0.4153861964203011	0.8345721053099385	0.2857755352871264	T	T	T
0.9153964926859067	0.3346919654737299	0.2856512791098469	T	T	T
0.1655598051268532	0.5848656329853933	0.2856535409389805	T	T	T
0.9156990303586893	0.8345887714563757	0.2855527413994803	T	T	T
0.1657887459280732	0.8345467551362504	0.2857457700085331	T	T	T
0.6657109188475527	0.3344785752884385	0.2857434858834514	T	T	T
0.6655541123998706	0.8347615860906760	0.2856278216170448	T	T	T
0.6657026963835037	0.0848791410647299	0.2857728898020345	T	T	T
0.1656023678643046	0.0849462838004997	0.2857316688895182	T	T	T
0.1656067287242034	0.3346779255501544	0.2856020432022805	T	T	T
0.9155299585162978	0.5847364678759562	0.2855708169766317	T	T	T
0.9154205595203787	0.0844917153143949	0.2855314287174983	T	T	T
0.6656747589390603	0.5845770717679650	0.2855576273397969	T	T	T
0.4155995738377317	0.0846839033765983	0.2854603729096646	T	T	T
0.4157978405437587	0.5848873377969213	0.2855391406100460	T	T	T
0.5827171602663083	0.9175468970292870	0.1914872691307202	T	T	T
0.0828082561481664	0.6674697319386094	0.1913411560683410	T	T	T
0.5826749674821473	0.4173028300943933	0.1914962050125854	T	T	T
0.0827793282882055	0.1674697616080419	0.1913536602304088	T	T	T
0.5829157568522818	0.1675054638338910	0.1913001500694230	T	T	T
0.0829677759686903	0.9176027906105445	0.1914874442433756	T	T	T
0.0827363333912876	0.4175097342330010	0.1914312831899432	T	T	T
0.5829531707407959	0.6675696583877989	0.1913252179585846	T	T	T
0.3326946075261186	0.1675790881410837	0.1913528709377215	T	T	T
0.3327489990700443	0.6675266608489999	0.1913724350207938	T	T	T
0.8327304490637533	0.1674961980129346	0.1913716735639192	T	T	T
0.8327747140068914	0.6674859967564339	0.1912728144354222	T	T	T
0.8326786503854288	0.9173050129830030	0.1913192969850382	T	T	T
0.3327821509997286	0.4174862446281907	0.1913560596188988	T	T	T
0.8327986926465810	0.4174541311918348	0.1913387575294516	T	T	T
0.3327698447325442	0.9173734906929949	0.1912995894729733	T	T	T

```
  0.2500000000000000  0.5000000000000000  0.0943600000000018   F   F   F
  0.7500000000000000  0.0000000000000000  0.0943600000000018   F   F   F
  0.2500000000000000  0.0000000000000000  0.0943600000000018   F   F   F
  0.7500000000000000  0.5000000000000000  0.0943600000000018   F   F   F
  0.7500000000000000  0.7500000000000000  0.0943600000000018   F   F   F
  0.5000000000000000  0.7500000000000000  0.0943600000000018   F   F   F
  0.5000000000000000  0.2500000000000000  0.0943600000000018   F   F   F
  0.0000000000000000  0.7500000000000000  0.0943600000000018   F   F   F
  0.0000000000000000  0.2500000000000000  0.0943600000000018   F   F   F
  0.7500000000000000  0.2500000000000000  0.0943600000000018   F   F   F
  0.2500000000000000  0.7500000000000000  0.0943600000000018   F   F   F
  0.2500000000000000  0.2500000000000000  0.0943600000000018   F   F   F
  0.5000000000000000  0.5000000000000000  0.0943600000000018   F   F   F
  0.5000000000000000  0.0000000000000000  0.0943600000000018   F   F   F
  0.0000000000000000  0.5000000000000000  0.0943600000000018   F   F   F
  0.0000000000000000  0.0000000000000000  0.0943600000000018   F   F   F

adsorption:
   1.00000000000000
    10.1184999999999992     0.0000000000000000     0.0000000000000000
    -5.0592499999999996     8.7628780481999993     0.0000000000000000
     0.0000000000000000     0.0000000000000000    21.1963000000000008
   Cu   C    H
    64     2     2
Selective dynamics
Direct
  0.9991935658779624  0.5014257334389877  0.3825797309798399   T   T   T
  0.4956225449080767  0.4991781279232990  0.3825732190752358   T   T   T
  0.4984684078114601  0.0021144573909836  0.3819685212904881   T   T   T
  0.0014859053278122  0.0050441912490062  0.3825886545595720   T   T   T
  0.4961720807485911  0.7513242386317068  0.3817288896113847   T   T   T
  0.7490081992156569  0.7516316781678161  0.3919422750956872   T   T   T
  0.0011407640973587  0.7515013485125349  0.3820830221214458   T   T   T
  0.7487605809288965  0.2518033181399763  0.3827063522410350   T   T   T
  0.2487985646557709  0.7518567102354275  0.3827195661529432   T   T   T
  0.7491050624235007  0.4994517279876358  0.3820605219890843   T   T   T
  0.9990743278586930  0.2524206635104561  0.3826114578619791   T   T   T
  0.7492769751076979  0.0043569769759854  0.3817341135454019   T   T   T
  0.2487245223253143  0.2519382228447953  0.3826093176613280   T   T   T
  0.2481423066630118  0.5014848919954120  0.3826203057434847   T   T   T
  0.4986327959704516  0.2519130357971918  0.3821107850944573   T   T   T
  0.2486807790716790  0.0020129256154426  0.3821369070006689   T   T   T
  0.4154635810101929  0.3346985591592969  0.2853751595493426   T   T   T
  0.4152889566039687  0.8348357687423947  0.2852880297151451   T   T   T
```

0.9156378998888686	0.3346967573950042	0.2854579300135756	T	T	T
0.1655679475376198	0.5846094373063377	0.2854678222384695	T	T	T
0.9135506904438114	0.8339807225483246	0.2877878443683117	T	T	T
0.1657898843535473	0.8347280638147502	0.2853843586486374	T	T	T
0.6655245917669097	0.3344406711098126	0.2853573194362742	T	T	T
0.6665111182713784	0.8337550704213347	0.2878127038231691	T	T	T
0.6654062231132594	0.0849499665443695	0.2853242789145471	T	T	T
0.1655768921796657	0.0847943987150971	0.2853927612691466	T	T	T
0.1654726038120378	0.3347540671733546	0.2853858468598424	T	T	T
0.9155535508766555	0.5846689183972537	0.2857527447750977	T	T	T
0.9155424348016400	0.0848492374597677	0.2854995959806179	T	T	T
0.6663724390746912	0.5868236314392589	0.2877746345801096	T	T	T
0.4152444535409444	0.0850423565374968	0.2851569779312234	T	T	T
0.4153916224462347	0.5846938825161658	0.2855030986701138	T	T	T
0.5834046766031430	0.9168322106411199	0.1918469030114933	T	T	T
0.0826690135808026	0.6676021403362881	0.1912338309843168	T	T	T
0.5834231108432703	0.4186451584442142	0.1917996836745196	T	T	T
0.0824913630666699	0.1672083034675223	0.1910988326999868	T	T	T
0.5825111097400236	0.1672806980330039	0.1909698991015603	T	T	T
0.0815958623355766	0.9167898403467369	0.1918039032543525	T	T	T
0.0827698389782460	0.4175180104000480	0.1909804558242509	T	T	T
0.5837463395580813	0.6679902214335270	0.1919843803034294	T	T	T
0.3326735801070157	0.1675342212767306	0.1910540561063140	T	T	T
0.3327268103804457	0.6673666438236365	0.1911415238969453	T	T	T
0.8328435645612594	0.1674993061064743	0.1911525421240900	T	T	T
0.8324965099405823	0.6677350784681009	0.1920713135398771	T	T	T
0.8322662525781565	0.9165061280409270	0.1919847650931417	T	T	T
0.3329953709235978	0.4177313393172505	0.1910972419626415	T	T	T
0.8326003572160295	0.4175584405535499	0.1912247130658397	T	T	T
0.3329076103487867	0.9177123774527105	0.1909711767284610	T	T	T
0.2500000000000000	0.5000000000000000	0.0943600000000018	F	F	F
0.7500000000000000	0.0000000000000000	0.0943600000000018	F	F	F
0.2500000000000000	0.0000000000000000	0.0943600000000018	F	F	F
0.7500000000000000	0.5000000000000000	0.0943600000000018	F	F	F
0.7500000000000000	0.7500000000000000	0.0943600000000018	F	F	F
0.5000000000000000	0.7500000000000000	0.0943600000000018	F	F	F
0.5000000000000000	0.2500000000000000	0.0943600000000018	F	F	F
0.0000000000000000	0.7500000000000000	0.0943600000000018	F	F	F
0.0000000000000000	0.2500000000000000	0.0943600000000018	F	F	F
0.7500000000000000	0.2500000000000000	0.0943600000000018	F	F	F
0.2500000000000000	0.7500000000000000	0.0943600000000018	F	F	F
0.2500000000000000	0.2500000000000000	0.0943600000000018	F	F	F
0.5000000000000000	0.5000000000000000	0.0943600000000018	F	F	F
0.5000000000000000	0.0000000000000000	0.0943600000000018	F	F	F

0.0000000000000000 0.5000000000000000 0.0943600000000018 F F F
0.0000000000000000 0.0000000000000000 0.0943600000000018 F F F
0.7834767483526816 0.7178303686485242 0.4972204864667958 T T T
0.7139875426908532 0.7883460227689416 0.4976561747886500 T T T
0.8433197794089490 0.6570921799521792 0.5062921688584212 T T T
0.6542132572751304 0.8494936142928863 0.5062414535293673 T T T

Hydrogenation path of C2H2 with low coverage on Cu model
CHCH:
1.00000000000000
10.1184999999999992 0.0000000000000000 0.0000000000000000
-5.0592499999999996 8.7628780481999993 0.0000000000000000
0.0000000000000000 0.0000000000000000 21.1963000000000008
Cu C H
64 2 10
Selective dynamics
Direct
0.9999432740926020 0.5007612789676167 0.3843813006481949 T T T
0.4956842510433753 0.5006644540751917 0.3853145904528261 T T T
0.4964753368380434 0.0042910023437072 0.3837395243823276 T T T
0.0005192009409204 0.0054176237046813 0.3852694343977082 T T T
0.4955252023439356 0.7510811373405042 0.3838098285848434 T T T
0.7489211976396030 0.7521096758140988 0.3914070910631957 T T T
0.0006660807668649 0.7511808700613131 0.3838146212351720 T T T
0.7479117127076824 0.2519330071690605 0.3779527940682974 T T T
0.2487553463111963 0.7527847818485639 0.3779177504284810 T T T
0.7495779876697459 0.5001535163790726 0.3837828737893740 T T T
0.9986993286632908 0.2529020841537158 0.3860291074492965 T T T
0.7498988282775061 0.0053488596705774 0.3837404646029124 T T T
0.2483912848573793 0.2525361352037973 0.3782724912866301 T T T
0.2480463516915509 0.5022992947002239 0.3859934758473528 T T T
0.4976211487244755 0.2519880178881448 0.3849559862035840 T T T
0.2489037282094445 0.0031901324399528 0.3848972959002899 T T T
0.4155048382679394 0.3356666206721412 0.2858626590451008 T T T
0.4154414649263397 0.8352957650149576 0.2856005369522236 T T T
0.9155693442613976 0.3345271253551295 0.2857499832491167 T T T
0.1657711949881454 0.5846785223672631 0.2857216975449700 T T T
0.9117637705473028 0.8338801387079555 0.2894487447202654 T T T
0.1653368300624073 0.8353471308558830 0.2855892201431540 T T T
0.6649666528250607 0.3351361387290446 0.2855839219998257 T T T
0.6671556799917935 0.8333511742114078 0.2892002895593879 T T T
0.6649573687148256 0.0848616753689486 0.2855597157557380 T T T
0.1648497153311770 0.0849474944746739 0.2858467862460360 T T T
0.1649279301331470 0.3354967709909488 0.2859064582174647 T T T

0.9155185794187980	0.5848693280542538	0.2867082043812463	T	T	T
0.9157421415562328	0.0855287016599661	0.2867578881470124	T	T	T
0.6667361734690912	0.5889105106148178	0.2894474987186226	T	T	T
0.4148001277777391	0.0855595596387586	0.2858239041246022	T	T	T
0.4149367967914076	0.5847416252953358	0.2867713034657884	T	T	T
0.5834340738856283	0.9167911631853006	0.1924285736047080	T	T	T
0.0831603200553078	0.6678000827321124	0.1912233999636505	T	T	T
0.5834721781489712	0.4192843385939653	0.1925222957469906	T	T	T
0.0823985971588363	0.1673982179788183	0.1912181988494401	T	T	T
0.5821810015099469	0.1673083874808993	0.1908855142254061	T	T	T
0.0810515644990762	0.9167644202998316	0.1925184749073528	T	T	T
0.0825845385473433	0.4175467365563008	0.1915173580965624	T	T	T
0.5836089555057109	0.6680316982294131	0.1922709216277278	T	T	T
0.3325516309503676	0.1677928005706848	0.1910800270002127	T	T	T
0.3324613633813136	0.6672932601434337	0.1911257344705178	T	T	T
0.8329873523350444	0.1678063734443967	0.1911366467805803	T	T	T
0.8324188085783818	0.6679638567225376	0.1923457904123507	T	T	T
0.8322704321375781	0.9166439372523972	0.1922686159862048	T	T	T
0.3328613068021248	0.4178815105499785	0.1912326808249848	T	T	T
0.8324528511198306	0.4171157293066360	0.1912124038807966	T	T	T
0.3330070040332864	0.9180739011881616	0.1908882907606996	T	T	T
0.2500000000000000	0.5000000000000000	0.0943600000000018	F	F	F
0.7500000000000000	0.0000000000000000	0.0943600000000018	F	F	F
0.2500000000000000	0.0000000000000000	0.0943600000000018	F	F	F
0.7500000000000000	0.5000000000000000	0.0943600000000018	F	F	F
0.7500000000000000	0.7500000000000000	0.0943600000000018	F	F	F
0.5000000000000000	0.7500000000000000	0.0943600000000018	F	F	F
0.5000000000000000	0.2500000000000000	0.0943600000000018	F	F	F
0.0000000000000000	0.7500000000000000	0.0943600000000018	F	F	F
0.0000000000000000	0.2500000000000000	0.0943600000000018	F	F	F
0.7500000000000000	0.2500000000000000	0.0943600000000018	F	F	F
0.2500000000000000	0.7500000000000000	0.0943600000000018	F	F	F
0.2500000000000000	0.2500000000000000	0.0943600000000018	F	F	F
0.5000000000000000	0.5000000000000000	0.0943600000000018	F	F	F
0.5000000000000000	0.0000000000000000	0.0943600000000018	F	F	F
0.0000000000000000	0.5000000000000000	0.0943600000000018	F	F	F
0.0000000000000000	0.0000000000000000	0.0943600000000018	F	F	F
0.7826745977606098	0.7190384441270624	0.4978047776979302	T	T	T
0.7132850135842136	0.7893148721407701	0.4982272421773197	T	T	T
0.8421920182503597	0.6584135501263900	0.5072261174966850	T	T	T
0.6540083233212327	0.8506426578853968	0.5070091647271506	T	T	T
0.0837109352639030	0.9192558158026244	0.4266243567765840	T	T	T
0.0820458294844728	0.4190851393665381	0.4294759464750255	T	T	T
0.5818671525940294	0.4174108358648672	0.4266457680216003	T	T	T

```
  0.5787436013455453  0.9223549543772100  0.4235869563673834 T T T
  0.4128256904380786  0.5856070517058022  0.4282288611999914 T T T
  0.9185179877662292  0.5823219792598771  0.4252357095104606 T T T
  0.4146261184540619  0.0861523501940745  0.4284307992914376 T T T
  0.9154007352274410  0.0880994058532671  0.4281411725614445 T T T
```

```
CHCH2:
   1.00000000000000
  10.1184999999999992    0.0000000000000000    0.0000000000000000
  -5.0592499999999996    8.7628780481999993    0.0000000000000000
   0.0000000000000000    0.0000000000000000   21.1963000000000008
Cu   C    H
64  2  10
Selective dynamics
Direct
  0.0004170776789963  0.4983221862395170  0.3857634879615166 T T T
  0.4980274649693852  0.4926440057526907  0.3814193272633935 T T T
  0.5020301492759741  0.0003175237022111  0.3854050304589544 T T T
  0.0054321599585102  0.0028965641602459  0.3837474466655189 T T T
  0.4984312742803891  0.7452971189536517  0.3842510798932309 T T T
  0.7449993304688743  0.7420701682892853  0.3870744852801146 T T T
  0.0010989473837650  0.7471416359448464  0.3842334632061750 T T T
  0.7496502400187296  0.2456671592841936  0.3823607893725516 T T T
  0.2529856047763953  0.7496788039525495  0.3782261674116933 T T T
  0.7526680533826112  0.4972556299343527  0.3832733515680180 T T T
  0.0035677333795331  0.2490369057065786  0.3860503640105207 T T T
  0.7513473804524979  0.9949845830607150  0.4003550803917401 T T T
  0.2524055163637441  0.2490103969855147  0.3783106705549797 T T T
  0.2469360474174514  0.4956146320305032  0.3843212721724675 T T T
  0.4992499624344125  0.2485879493620267  0.3825205948508031 T T T
  0.2514616439703932  0.9974682697184760  0.3845321074454953 T T T
  0.4188786001881012  0.3323135279770146  0.2857527076642379 T T T
  0.4220924700371882  0.8353511766597986  0.2878422412830287 T T T
  0.9162251340029002  0.3320284849560368  0.2877821585945151 T T T
  0.1660001698973356  0.5825594159744736  0.2854286390634533 T T T
  0.9146440399234932  0.8317858180187586  0.2894078496285852 T T T
  0.1669973352743689  0.8322095547806031  0.2855274710107779 T T T
  0.6686917516456716  0.3332619286307962  0.2880254937623619 T T T
  0.6696219417689553  0.8347817142137405  0.2902359630998558 T T T
  0.6693782042634707  0.0813236473178710  0.2877678809214357 T T T
  0.1671432451169595  0.0824828914529147  0.2852102834336842 T T T
  0.1672223791042953  0.3333621325796737  0.2853568451100370 T T T
  0.9212850497181306  0.5846314753790431  0.2855800769740934 T T T
  0.9168488169118439  0.0831275287029723  0.2877165734610775 T T T
```

0.6701661676843558	0.5853552975197475	0.2886614238456767	T	T	T
0.4165643459442077	0.0807594849629582	0.2850716321771442	T	T	T
0.4167427356864156	0.5837437658070836	0.2853837088649779	T	T	T
0.5849302728426832	0.9156062875852836	0.1927181861009943	T	T	T
0.0842995083517526	0.6667314352845387	0.1899976188764497	T	T	T
0.5855538079931621	0.4174822554594451	0.1926270467752475	T	T	T
0.0841294434288793	0.1679467666744986	0.1912680601657011	T	T	T
0.5841866929161342	0.1666160844040964	0.1921879518479334	T	T	T
0.0816786768213845	0.9157557784549324	0.1919455659313270	T	T	T
0.0835623100477720	0.4168984692499690	0.1914636152296634	T	T	T
0.5848648861244997	0.6669006536589239	0.1924949262460411	T	T	T
0.3344859924328998	0.1668151103019925	0.1908750371758139	T	T	T
0.3338912415064269	0.6671517104896851	0.1914813855201047	T	T	T
0.8336285018327244	0.1655536111588394	0.1921743667390294	T	T	T
0.8331920847055715	0.6659600226983973	0.1919704090279814	T	T	T
0.8338258998800825	0.9162834147678132	0.1930185888846067	T	T	T
0.3347376486218188	0.4157518391195890	0.1912085762521444	T	T	T
0.8341812256510215	0.4159692046071407	0.1917371457646283	T	T	T
0.3343165968232520	0.9162921427177776	0.1912981467382631	T	T	T
0.2500000000000000	0.5000000000000000	0.0943600000000018	F	F	F
0.7500000000000000	0.0000000000000000	0.0943600000000018	F	F	F
0.2500000000000000	0.0000000000000000	0.0943600000000018	F	F	F
0.7500000000000000	0.5000000000000000	0.0943600000000018	F	F	F
0.7500000000000000	0.7500000000000000	0.0943600000000018	F	F	F
0.5000000000000000	0.7500000000000000	0.0943600000000018	F	F	F
0.5000000000000000	0.2500000000000000	0.0943600000000018	F	F	F
0.0000000000000000	0.7500000000000000	0.0943600000000018	F	F	F
0.0000000000000000	0.2500000000000000	0.0943600000000018	F	F	F
0.7500000000000000	0.2500000000000000	0.0943600000000018	F	F	F
0.2500000000000000	0.7500000000000000	0.0943600000000018	F	F	F
0.2500000000000000	0.2500000000000000	0.0943600000000018	F	F	F
0.5000000000000000	0.5000000000000000	0.0943600000000018	F	F	F
0.5000000000000000	0.0000000000000000	0.0943600000000018	F	F	F
0.0000000000000000	0.5000000000000000	0.0943600000000018	F	F	F
0.0000000000000000	0.0000000000000000	0.0943600000000018	F	F	F
0.7666888040473203	0.7890823584588949	0.5011523676084038	T	T	T
0.6760507005359064	0.8228998281676158	0.4646279280528021	T	T	T
0.8894137667872677	0.8395762608053168	0.4930600092522684	T	T	T
0.5587817479629806	0.7729872745673966	0.4835561184383863	T	T	T
0.0859367211475766	0.9151739371525748	0.4260117532963404	T	T	T
0.0815535619174642	0.4142559128377648	0.4291215795149059	T	T	T
0.7214933800022538	0.7137410369397085	0.5421731662848558	T	T	T
0.6661233960336702	0.0886483387866447	0.4300166287892382	T	T	T
0.4117677647031197	0.5700350005726866	0.4230878558989044	T	T	T

```
  0.9132239332411576  0.5795050585481344  0.4285118601199844 T T T
  0.4167514974439430  0.0836921777642482  0.4264597999742488 T T T
  0.9353497707728892  0.0907527349978452  0.4310711216914181 T T T

CH2CH2:
   1.00000000000000
  10.1184999999999992   0.0000000000000000   0.0000000000000000
  -5.0592499999999996   8.7628780481999993   0.0000000000000000
   0.0000000000000000   0.0000000000000000  21.1963000000000008
Cu   C    H
64  2  10
Selective dynamics
Direct
  0.0023151865959603  0.5007897871116627  0.3872977442043885 T T T
  0.4992163480052181  0.4997935470597492  0.3828252038196759 T T T
  0.5015677755455573  0.0019236391345177  0.3829894725710652 T T T
  0.0031071966628769  0.0038780638912791  0.3867095581256971 T T T
  0.4990595801791722  0.7516394093752907  0.3827864255592072 T T T
  0.7478670499873176  0.7488543740564522  0.3917357392396361 T T T
  0.0042635895936988  0.7515712799647684  0.3867583893296635 T T T
  0.7493689403221172  0.2503494285488655  0.3802950615261901 T T T
  0.2538704328564466  0.7532359466310770  0.3791424234672692 T T T
  0.7544065852356234  0.5011863265041473  0.3829484098085483 T T T
  0.0022560191051447  0.2535242620979366  0.3873928016037496 T T T
  0.7542700403459790  0.0044055562916693  0.3830787997891936 T T T
  0.2535325887754912  0.2525441780328300  0.3792430230542717 T T T
  0.2502777340549308  0.5013058300116232  0.3870954261272170 T T T
  0.5015860016357372  0.2515217822374795  0.3829993899772617 T T T
  0.2512892433986685  0.0018787265250806  0.3868186459908855 T T T
  0.4194215595154309  0.3352359719500110  0.2866519756782418 T T T
  0.4196285472464543  0.8353706671847725  0.2864789028348688 T T T
  0.9180806982886904  0.3344841196285951  0.2873608100907399 T T T
  0.1674034016720703  0.5838027587258543  0.2866734619314972 T T T
  0.9146106565315278  0.8326870941359168  0.2904966575111614 T T T
  0.1672468966867652  0.8345877559535091  0.2865221940962188 T T T
  0.6678638133185265  0.3353078467061431  0.2872529493992191 T T T
  0.6694329712708914  0.8335279626770755  0.2900951446345069 T T T
  0.6679189028189133  0.0833789117895658  0.2872526374491129 T T T
  0.1671182863279689  0.0837028593364220  0.2866028755834584 T T T
  0.1672152885570406  0.3346236740681033  0.2867892614300122 T T T
  0.9213281126550568  0.5861005782815640  0.2860337148008696 T T T
  0.9213168079922492  0.0860693889898462  0.2860496420580175 T T T
  0.6691124068575672  0.5864018536592237  0.2899372258931892 T T T
  0.4145375490734792  0.0827929455316028  0.2845315145850256 T T T
```

0.4151623504889021	0.5830943052396216	0.2850938908294547	T	T	T
0.5856861765106112	0.9170077343445762	0.1925371484723170	T	T	T
0.0846686490998400	0.6677780103390134	0.1912437167833421	T	T	T
0.5855017851072880	0.4192378426725877	0.1925404239789133	T	T	T
0.0848259686821392	0.1676073481857272	0.1912940061269647	T	T	T
0.5841670075794438	0.1674291628428642	0.1918033491690647	T	T	T
0.0816980170745986	0.9162189135696180	0.1927157095266019	T	T	T
0.0832342181193717	0.4170127341145183	0.1918994503469984	T	T	T
0.5852038115325756	0.6678578846716985	0.1926593999785687	T	T	T
0.3341108736117004	0.1678968117119607	0.1910751276759351	T	T	T
0.3341500304306671	0.6677723341287420	0.1912590499511118	T	T	T
0.8336024544446439	0.1673569889132907	0.1920345756326178	T	T	T
0.8332734372934135	0.6675964431777931	0.1929817202752457	T	T	T
0.8334466301715889	0.9163021277720906	0.1929623891284619	T	T	T
0.3339167317174062	0.4169745818648484	0.1912641981412233	T	T	T
0.8337162298145663	0.4168496785258731	0.1920081785395881	T	T	T
0.3340858364665892	0.9168081250829490	0.1910412057901899	T	T	T
0.2500000000000000	0.5000000000000000	0.0943600000000018	F	F	F
0.7500000000000000	0.0000000000000000	0.0943600000000018	F	F	F
0.2500000000000000	0.0000000000000000	0.0943600000000018	F	F	F
0.7500000000000000	0.5000000000000000	0.0943600000000018	F	F	F
0.7500000000000000	0.7500000000000000	0.0943600000000018	F	F	F
0.5000000000000000	0.7500000000000000	0.0943600000000018	F	F	F
0.5000000000000000	0.2500000000000000	0.0943600000000018	F	F	F
0.0000000000000000	0.7500000000000000	0.0943600000000018	F	F	F
0.0000000000000000	0.2500000000000000	0.0943600000000018	F	F	F
0.7500000000000000	0.2500000000000000	0.0943600000000018	F	F	F
0.2500000000000000	0.7500000000000000	0.0943600000000018	F	F	F
0.2500000000000000	0.2500000000000000	0.0943600000000018	F	F	F
0.5000000000000000	0.5000000000000000	0.0943600000000018	F	F	F
0.5000000000000000	0.0000000000000000	0.0943600000000018	F	F	F
0.0000000000000000	0.5000000000000000	0.0943600000000018	F	F	F
0.0000000000000000	0.0000000000000000	0.0943600000000018	F	F	F
0.7102260603072322	0.6617725272221522	0.5008053397765228	T	T	T
0.7543251740369281	0.8125755855491562	0.5005280191245834	T	T	T
0.7932941845746185	0.6239024953876648	0.5057756403779791	T	T	T
0.6714122538570730	0.8514959516322202	0.5018495417933112	T	T	T
0.0861063142785439	0.9190397393391696	0.4302757963800634	T	T	T
0.0863820082795849	0.4194020498726279	0.4318332801621669	T	T	T
0.5900219563099938	0.5739364195826403	0.5023644965104852	T	T	T
0.8739391096791643	0.8998577153820719	0.5056553034983344	T	T	T
0.4206053634025780	0.5875377158419242	0.4267847151890288	T	T	T
0.9198177976586512	0.5835742578303335	0.4292438553188963	T	T	T
0.4229225099197306	0.0875506771627480	0.4270579248873256	T	T	T

0.9187064782583716 0.0891630553996365 0.4293685777620495 T T T

Hydrogenation path of C2H2 with high coverage on Cu model
CHCH:
1.00000000000000
10.1184999999999992   0.0000000000000000   0.0000000000000000
-5.0592499999999996   8.7628780481999993   0.0000000000000000
0.0000000000000000   0.0000000000000000 21.1963000000000008
Cu C H
64 8 16
Selective dynamics
Direct
0.9993609357026808 0.5021201602489489 0.3831196638800258 T T T
0.4995142151216143 0.5020891438443692 0.3832064332654059 T T T
0.4995276715211467 0.0020956887344185 0.3831758653320214 T T T
0.9993622318448160 0.0021866766938810 0.3830832631882470 T T T
0.4994339730536011 0.7504043386610845 0.3829492048266435 T T T
0.7501152522705855 0.7516392282031250 0.3879912202372610 T T T
0.9993726153655494 0.7503755511435442 0.3828414488395947 T T T
0.7500940920735099 0.2515974090495230 0.3879185122716549 T T T
0.2500003644759537 0.7516522717182481 0.3882107967839468 T T T
0.7507591774149808 0.5018797962705679 0.3828944543152714 T T T
0.9994000432658866 0.2503696200007243 0.3829020531802741 T T T
0.7507824616986248 0.0019089926858033 0.3829174997401055 T T T
0.2500563873874336 0.2516366004423377 0.3883132117854338 T T T
0.2508931852330070 0.5019809845477093 0.3829116281786563 T T T
0.4995161029709154 0.2504650728661624 0.3829318819024823 T T T
0.2509175798737456 0.0019818913032854 0.3828488819387743 T T T
0.4130944213808532 0.3334605065348328 0.2878229183863593 T T T
0.4131113963265761 0.8334522919791478 0.2878114490722834 T T T
0.9131749789082320 0.3335066753672967 0.2877149671903648 T T T
0.1674258688457016 0.5878664895401313 0.2877533054131128 T T T
0.9131639364710272 0.8335104114803293 0.2876938232936019 T T T
0.1672998538747671 0.8336114364665065 0.2876258374054470 T T T
0.6672554526279971 0.3336551313870170 0.2876103100287675 T T T
0.6672462186985876 0.8336227249615111 0.2876508265559212 T T T
0.6674023623308624 0.0878096428510843 0.2876793863297417 T T T
0.1674072645175036 0.0878642955271857 0.2877289864034456 T T T
0.1673203572471852 0.3336039093237270 0.2876954912622705 T T T
0.9159171445490022 0.5848025495130917 0.2859298345206177 T T T
0.9159094148494216 0.0848038185634595 0.2859198992458927 T T T
0.6673745287232437 0.5877976931886824 0.2877017336872578 T T T
0.4159482151318966 0.0847952661722793 0.2859458564487523 T T T
0.4159394832042601 0.5847530784040060 0.2859703765355472 T T T

0.5830274085991438	0.9173453420568152	0.1936285129189513	T	T	T
0.0833774198381890	0.6676512949332631	0.1915092337050176	T	T	T
0.5829815973421597	0.4173537834842062	0.1936256015064409	T	T	T
0.0833783112437577	0.1676920445300591	0.1915328037029702	T	T	T
0.5833469121673047	0.1676700450068053	0.1915063841052804	T	T	T
0.0830299642187140	0.9173802241387050	0.1936050364123816	T	T	T
0.0830447238336299	0.4173496566900421	0.1936275139917742	T	T	T
0.5833443559499878	0.6676773969059772	0.1915213712514020	T	T	T
0.3326951983600339	0.1678033529077534	0.1915184694474095	T	T	T
0.3326831154972375	0.6678314705398757	0.1915293377818906	T	T	T
0.8326929893628082	0.1678147754969185	0.1914814608005271	T	T	T
0.8326777812926988	0.6677891281578174	0.1914745628025052	T	T	T
0.8327775132160760	0.9171486777385373	0.1915010432314283	T	T	T
0.3328017400039766	0.4171014546499255	0.1915259394637063	T	T	T
0.8327944337149441	0.4171125941102716	0.1914906470152215	T	T	T
0.3328375006617466	0.9171058129689906	0.1915095571675940	T	T	T
0.2500000000000000	0.5000000000000000	0.0943600000000018	F	F	F
0.7500000000000000	0.0000000000000000	0.0943600000000018	F	F	F
0.2500000000000000	0.0000000000000000	0.0943600000000018	F	F	F
0.7500000000000000	0.5000000000000000	0.0943600000000018	F	F	F
0.7500000000000000	0.7500000000000000	0.0943600000000018	F	F	F
0.5000000000000000	0.7500000000000000	0.0943600000000018	F	F	F
0.5000000000000000	0.2500000000000000	0.0943600000000018	F	F	F
0.0000000000000000	0.7500000000000000	0.0943600000000018	F	F	F
0.0000000000000000	0.2500000000000000	0.0943600000000018	F	F	F
0.7500000000000000	0.2500000000000000	0.0943600000000018	F	F	F
0.2500000000000000	0.7500000000000000	0.0943600000000018	F	F	F
0.2500000000000000	0.2500000000000000	0.0943600000000018	F	F	F
0.5000000000000000	0.5000000000000000	0.0943600000000018	F	F	F
0.5000000000000000	0.0000000000000000	0.0943600000000018	F	F	F
0.0000000000000000	0.5000000000000000	0.0943600000000018	F	F	F
0.0000000000000000	0.0000000000000000	0.0943600000000018	F	F	F
0.7846465867691785	0.2172707359969719	0.5016024481283378	T	T	T
0.7151129724098100	0.2868567380476931	0.5022124150948191	T	T	T
0.2842008256911213	0.2170033215448973	0.5011881037138239	T	T	T
0.7842095314061400	0.7168588544302623	0.5013213109560577	T	T	T
0.2152711301762699	0.2872637079072818	0.5018369721679630	T	T	T
0.7154282610484971	0.7872345279598881	0.5019434931835257	T	T	T
0.2839861601099365	0.7175857769872283	0.5011968128354316	T	T	T
0.2139052262136393	0.7867070378531964	0.5019179708128970	T	T	T
0.8459832113794696	0.1569766388004800	0.5066939495304568	T	T	T
0.3444053819297379	0.1556380128905181	0.5066553588063463	T	T	T
0.8447000495465794	0.6557548855017018	0.5066042713351063	T	T	T
0.3453969114288628	0.6574695073814476	0.5066823325263834	T	T	T

```
  0.6536078784997208  0.3468638988166535  0.5077723732258460 T T T
  0.1546851455807400  0.3481169286207905  0.5076411589911389 T T T
  0.6545037806917617  0.8478407748221314  0.5076439981428577 T T T
  0.0816577197957854  0.9198031730377104  0.4244858520597630 T T T
  0.0816405842189903  0.4197524342603683  0.4245943226153018 T T T
  0.5818510758779832  0.4198583229838891  0.4247858313781037 T T T
  0.5818535331346745  0.9199479653837932  0.4247559899681317 T T T
  0.1523030077093618  0.8465661960582287  0.5076393658560816 T T T
  0.4180927230390156  0.5831784756220917  0.4263803835901861 T T T
  0.9179869523354820  0.5832405082527369  0.4262521872892454 T T T
  0.4181454104705910  0.0832897588626861  0.4262870622594273 T T T
  0.9180048404326334  0.0831569721891582  0.4263425817769098 T T T

CHCH2:
   1.00000000000000
  10.1184999999999992   0.0000000000000000   0.0000000000000000
  -5.0592499999999996   8.7628780481999993   0.0000000000000000
   0.0000000000000000   0.0000000000000000  21.1963000000000008
Cu   C    H
64  8  16
Selective dynamics
Direct
  0.9937681220464104  0.4966935391424291  0.3837948805364519 T T T
  0.4987807611953552  0.4920708351118836  0.3801889621506258 T T T
  0.5007765927725331  0.0000097643603765  0.3840822682541670 T T T
  0.9988855557318752  0.0032567821405567  0.3824242550613841 T T T
  0.4983713226187086  0.7423175891103557  0.3834084856816287 T T T
  0.7436083809328877  0.7424312087114558  0.3872792735636980 T T T
  0.9965642566220504  0.7478563913598459  0.3829155450209231 T T T
  0.7466622569496049  0.2447635935305616  0.3840278974784285 T T T
  0.2515890490191465  0.7486864177862230  0.3921287473823585 T T T
  0.7495667833170648  0.4968915366644093  0.3826299165549301 T T T
  0.9983097830457862  0.2482449146209323  0.3834519002195741 T T T
  0.7475500843771073  0.9934534726368480  0.4000508577840497 T T T
  0.2512350700742499  0.2485984154539412  0.3895983822409729 T T T
  0.2453952975432005  0.4949985605453137  0.3820325376829537 T T T
  0.4977210922172483  0.2465980414231342  0.3810357635345078 T T T
  0.2488377123730347  0.9974660692434431  0.3824015380456769 T T T
  0.4132388866299690  0.3294381422103775  0.2877154737065419 T T T
  0.4149350712302546  0.8316209453828564  0.2907759195065902 T T T
  0.9141667835723982  0.3323799740233637  0.2872003082635765 T T T
  0.1659081939431349  0.5857932417747387  0.2879855516344469 T T T
  0.9132407624422588  0.8327146603971975  0.2882059389065610 T T T
  0.1673559210649316  0.8296369528165368  0.2882848453560492 T T T
```

0.6669435877894547	0.3325814626353978	0.2878095162621787	T	T	T
0.6683210522276645	0.8350803227620797	0.2898600329863210	T	T	T
0.6676005610728387	0.0815602529338205	0.2883043412359157	T	T	T
0.1667452388950616	0.0850752424523137	0.2871679264278283	T	T	T
0.1671056695987206	0.3310726429633452	0.2869973670474643	T	T	T
0.9187503031991427	0.5846685921391902	0.2852675900224447	T	T	T
0.9148527011169104	0.0834459552596773	0.2866975435206068	T	T	T
0.6684824779562050	0.5857041578940940	0.2882325384464123	T	T	T
0.4149916789090020	0.0797081447189664	0.2848580821614450	T	T	T
0.4154265521556436	0.5837970341983355	0.2852057347861184	T	T	T
0.5824799645848470	0.9149374639602230	0.1938965135468134	T	T	T
0.0838209431303726	0.6666847091219394	0.1908014643741696	T	T	T
0.5834101082239888	0.4166267144903472	0.1934972513990720	T	T	T
0.0835697386433565	0.1678248891761456	0.1914904082600429	T	T	T
0.5834494408111179	0.1661691065442182	0.1920730649488706	T	T	T
0.0826862241692473	0.9164041226890848	0.1932887616793146	T	T	T
0.0839520546869340	0.4175211023118035	0.1928496039725071	T	T	T
0.5842774781136184	0.6676956971489871	0.1922978846211539	T	T	T
0.3337246144633549	0.1671237739400574	0.1914238730193389	T	T	T
0.3328883946808787	0.6670649809218584	0.1919880856136724	T	T	T
0.8323398147231676	0.1657447055876101	0.1918638815002595	T	T	T
0.8320570408480399	0.6658983202243026	0.1916492781002118	T	T	T
0.8337531658322874	0.9173121031119472	0.1922809149160641	T	T	T
0.3336094176783735	0.4149901257123438	0.1916707039041862	T	T	T
0.8336167630996486	0.4169417075436691	0.1914837913723694	T	T	T
0.3327356530854681	0.9152176246812848	0.1918318751591082	T	T	T
0.2500000000000000	0.5000000000000000	0.0943600000000018	F	F	F
0.7500000000000000	0.0000000000000000	0.0943600000000018	F	F	F
0.2500000000000000	0.0000000000000000	0.0943600000000018	F	F	F
0.7500000000000000	0.5000000000000000	0.0943600000000018	F	F	F
0.7500000000000000	0.7500000000000000	0.0943600000000018	F	F	F
0.5000000000000000	0.7500000000000000	0.0943600000000018	F	F	F
0.5000000000000000	0.2500000000000000	0.0943600000000018	F	F	F
0.0000000000000000	0.7500000000000000	0.0943600000000018	F	F	F
0.0000000000000000	0.2500000000000000	0.0943600000000018	F	F	F
0.7500000000000000	0.2500000000000000	0.0943600000000018	F	F	F
0.2500000000000000	0.7500000000000000	0.0943600000000018	F	F	F
0.2500000000000000	0.2500000000000000	0.0943600000000018	F	F	F
0.5000000000000000	0.5000000000000000	0.0943600000000018	F	F	F
0.5000000000000000	0.0000000000000000	0.0943600000000018	F	F	F
0.0000000000000000	0.5000000000000000	0.0943600000000018	F	F	F
0.0000000000000000	0.0000000000000000	0.0943600000000018	F	F	F
0.7691357506744124	0.2748882507313590	0.5256153838944621	T	T	T
0.7185176887764563	0.3604866841994528	0.5201099059963599	T	T	T

```
  0.3083660366088447  0.2348431069578265  0.5023143302983643 T T T
  0.7694034209343666  0.7955377364635401  0.5030957412413475 T T T
  0.2148644158713488  0.2773238024330550  0.5036261597921690 T T T
  0.6769873808428345  0.8239978833458056  0.4653269374834269 T T T
  0.2689428733681211  0.6997977108475589  0.4914968334516909 T T T
  0.2198680686177997  0.7895383553526254  0.4920052754700218 T T T
  0.8136701208488291  0.1988521211489191  0.5297423798088301 T T T
  0.3876388337681241  0.1947377900083244  0.5084051481294624 T T T
  0.8918537128564078  0.8459829580045656  0.4942963325868707 T T T
  0.3081276213905555  0.6232389673428674  0.5056825423896611 T T T
  0.6742230551967839  0.4365513890597033  0.5155725765496330 T T T
  0.1337775266999166  0.3148758409834665  0.5095431085783971 T T T
  0.5605871782969328  0.7744052797900856  0.4846441293373935 T T T
  0.0782729085695017  0.9188860146474340  0.4231379942594677 T T T
  0.0734541491788796  0.4118668776377840  0.4259569528158153 T T T
  0.7264843211957639  0.7249217575262829  0.5454785785816907 T T T
  0.6583184773383222  0.0806708323010042  0.4301846435341425 T T T
  0.1767228074665112  0.8631772643238339  0.5051846352592397 T T T
  0.4116633304461202  0.5670710316029844  0.4210087934856201 T T T
  0.9104607081481152  0.5835779070402188  0.4272434422200612 T T T
  0.4150925371831840  0.0819022699132866  0.4241684044816457 T T T
  0.9317683356129680  0.0935055589609220  0.4307308359315111 T T T

CH2CH2:
   1.00000000000000
  10.1184999999999992    0.0000000000000000    0.0000000000000000
  -5.0592499999999996    8.7628780481999993    0.0000000000000000
   0.0000000000000000    0.0000000000000000   21.1963000000000008
Cu   C    H
64  8  16
Selective dynamics
Direct
  0.0014578270261626  0.5043019689226691  0.3842233294625920 T T T
  0.5015500851888463  0.5004680189327045  0.3817337929283830 T T T
  0.4999818441169359  0.9975573402106310  0.3822453958174704 T T T
  0.9998139243135480  0.0009760987332198  0.3838218686122805 T T T
  0.5002386275097737  0.7491018030267903  0.3809931366607541 T T T
  0.7466568613257009  0.7486691550531808  0.3920217182699264 T T T
  0.0002630659810490  0.7509452350025324  0.3842005359855278 T T T
  0.7461846151664965  0.2485575430029301  0.3932065078867889 T T T
  0.2544350200630858  0.7523051044823399  0.3912678199532553 T T T
  0.7536140941938729  0.5024167809145865  0.3820428539499735 T T T
  0.0007127353013293  0.2518375577918561  0.3837711220383064 T T T
  0.7542570772868586  0.0026340570417656  0.3824026076481479 T T T
```

0.2552547658393901	0.2527593487415796	0.3938695816547335	T	T	T
0.2500783081880041	0.5005884878593450	0.3830972882336217	T	T	T
0.4993729653786209	0.2473502765555274	0.3813992854821366	T	T	T
0.2484531403418303	0.9995915738308589	0.3832242108271121	T	T	T
0.4144255996039012	0.3331282025309544	0.2894123737395477	T	T	T
0.4146816988813721	0.8328589041080700	0.2886684366288206	T	T	T
0.9134659977652612	0.3337253326638186	0.2894389296436112	T	T	T
0.1679148927255362	0.5871587166962285	0.2879742945457655	T	T	T
0.9143016890106894	0.8332646000758652	0.2888739326344071	T	T	T
0.1673660527928606	0.8324912907952088	0.2881033901995838	T	T	T
0.6679083975739508	0.3337256756837893	0.2894970166029766	T	T	T
0.6689792685267090	0.8342893037307970	0.2893315692845825	T	T	T
0.6684712486160034	0.0871986154746901	0.2898538317283255	T	T	T
0.1682308126967612	0.0876925438977035	0.2884339758098585	T	T	T
0.1679432885245076	0.3324145681010867	0.2885498757829695	T	T	T
0.9195034674687566	0.5858102186312515	0.2854062560491860	T	T	T
0.9199623106531558	0.0863974625867061	0.2854313466156567	T	T	T
0.6684037808873616	0.5867352525330396	0.2893992340350556	T	T	T
0.4144964484707286	0.0827147173529005	0.2847320204090474	T	T	T
0.4141750346260519	0.5827295156347497	0.2842610430935315	T	T	T
0.5844853561832223	0.9176645745963103	0.1939941968819217	T	T	T
0.0833829208110687	0.6669919415107692	0.1912811491473985	T	T	T
0.5839319908704452	0.4171605841157012	0.1940095846983561	T	T	T
0.0850290302647534	0.1681401582216528	0.1914993958543727	T	T	T
0.5847434564972344	0.1678097755237047	0.1924592944227143	T	T	T
0.0830953787495197	0.9170946250785764	0.1937244039484311	T	T	T
0.0828370592596910	0.4167977068057183	0.1938400642904672	T	T	T
0.5846037693035284	0.6676200680449770	0.1923002931732785	T	T	T
0.3337209036007125	0.1678039907852272	0.1917366667247024	T	T	T
0.3337607979266147	0.6682545501839052	0.1914789080316523	T	T	T
0.8327141066284863	0.1675069477938419	0.1924515384222824	T	T	T
0.8327812121845904	0.6677875192629578	0.1922783031124687	T	T	T
0.8330914163995280	0.9171164414323936	0.1922668025604300	T	T	T
0.3333846675397551	0.4161108223764138	0.1916479210600082	T	T	T
0.8327737456744955	0.4166932916156525	0.1923586799921555	T	T	T
0.3333368919670718	0.9160890472340364	0.1917093049487494	T	T	T
0.2500000000000000	0.5000000000000000	0.0943600000000018	F	F	F
0.7500000000000000	0.0000000000000000	0.0943600000000018	F	F	F
0.2500000000000000	0.0000000000000000	0.0943600000000018	F	F	F
0.7500000000000000	0.5000000000000000	0.0943600000000018	F	F	F
0.7500000000000000	0.7500000000000000	0.0943600000000018	F	F	F
0.5000000000000000	0.7500000000000000	0.0943600000000018	F	F	F
0.5000000000000000	0.2500000000000000	0.0943600000000018	F	F	F
0.0000000000000000	0.7500000000000000	0.0943600000000018	F	F	F

0.0000000000000000	0.2500000000000000	0.0943600000000018	F	F	F
0.7500000000000000	0.2500000000000000	0.0943600000000018	F	F	F
0.2500000000000000	0.7500000000000000	0.0943600000000018	F	F	F
0.2500000000000000	0.2500000000000000	0.0943600000000018	F	F	F
0.5000000000000000	0.5000000000000000	0.0943600000000018	F	F	F
0.5000000000000000	0.0000000000000000	0.0943600000000018	F	F	F
0.0000000000000000	0.5000000000000000	0.0943600000000018	F	F	F
0.0000000000000000	0.0000000000000000	0.0943600000000018	F	F	F
0.7648476359675812	0.2153460653188483	0.4919036976154526	T	T	T
0.6966871634216734	0.2883605505038779	0.4879898500234430	T	T	T
0.2913471752547432	0.2200780357089198	0.4963881592542903	T	T	T
0.7089929838578404	0.6629556236918878	0.5015041581461381	T	T	T
0.2237073308671742	0.2926291762952456	0.4969467861727565	T	T	T
0.7524595071716546	0.8135413440099984	0.5015421402567956	T	T	T
0.3060704922728919	0.7333770711644760	0.5002821193037920	T	T	T
0.2224954699761327	0.7882749759475749	0.5011785239137365	T	T	T
0.8231595226485789	0.1582897999278498	0.5089540784897377	T	T	T
0.3473337033950414	0.1567600647540451	0.5075275783031584	T	T	T
0.7930200202054457	0.6262376608595747	0.5061471824557346	T	T	T
0.3749348801439371	0.6816258556666079	0.5072722759366926	T	T	T
0.6390888728378595	0.3487703603065518	0.5017363986173918	T	T	T
0.1647723689871322	0.3536999962113169	0.5067520337754223	T	T	T
0.6692065211268547	0.8521162009988589	0.5024110419283301	T	T	T
0.0777364834297637	0.9178398997839196	0.4282400589094995	T	T	T
0.0809692377290026	0.4213595356165505	0.4271660093538801	T	T	T
0.5889047764529427	0.5746302762098613	0.5026841256386929	T	T	T
0.8720087434871377	0.9012300652272672	0.5058653696527212	T	T	T
0.1504265612946907	0.8366168744829602	0.5079185196740000	T	T	T
0.4199563258961433	0.5833636554016365	0.4242712526873401	T	T	T
0.9216584683842520	0.5877030587052208	0.4288807275825685	T	T	T
0.4162504485186856	0.0789201424352851	0.4239265999913215	T	T	T
0.9228931279071416	0.0867273822193361	0.4272922244761343	T	T	T
